# Exploring the role of meaning in non-Māori speakers’ ‘proto-lexicon’

**DOI:** 10.1371/journal.pone.0339325

**Published:** 2026-01-29

**Authors:** Wakayo Mattingley, Forrest Panther, Simon Todd, Jennifer Hay, Jeanette King, Peter J. Keegan

**Affiliations:** 1 New Zealand Institute of Language, Brain and Behaviour, University of Canterbury, Christchurch, New Zealand; 2 Department of Linguistics, University of Canterbury, Christchurch, New Zealand; 3 Department of Linguistics, University of California, Santa Barbara, California, United States of America; 4 Aotahi: School of Māori and Indigenous Studies, University of Canterbury, Christchurch, New Zealand; 5 Te Puna Wānanga, Faculty of Arts and Education, University of Auckland, Auckland, New Zealand; Universiteit van Amsterdam, NETHERLANDS, KINGDOM OF THE

## Abstract

Previous work has demonstrated that New Zealanders who do not speak Māori but are regularly exposed to the language develop implicit knowledge of it. The core of this knowledge, it has been argued, is the ‘proto-lexicon’—a set of stored word-forms, without associated meaning, which yields subsequent Māori phonotactic and morphological knowledge. Previous research shows that having a proto-lexicon gives learners a head start in learning Māori word meanings in formal education. We investigate experimentally whether the proto-lexicon confers an advantage for attaching meanings to words. In Experiment 1, non-Māori-speaking New Zealanders were tested on their ability to identify meanings of Māori words in a forced-choice definition task, and they did this relatively well. Then, words with low accuracy were selected for Experiment 2, where non-Māori-speaking New Zealanders and non-New Zealanders were asked to learn meanings for Māori words and nonwords. New Zealanders performed better, indicating that familiarity with Māori word shapes confers an advantage. However, they showed no greater advantage for real words over nonwords. If these words are definitely in the proto-lexicon, then this would suggest that knowledge of individual word-forms does not, in fact, confer an advantage. In Experiment 3, we therefore explore whether the words in Experiment 2 are actually robustly in the participants’ proto-lexicon, by running a word identification task with the same participants. These words were not robustly distinguished from nonwords. By selecting words for their lack of semantic knowledge, we also inadvertently selected words that do not appear to be in the proto-lexicon. Together, our results indicate that different levels of semantic knowledge exist for different words, even when we consider only words that cannot confidently be said to be in a full lexicon. The results suggest that the claim of previous studies that the proto-lexicon is ‘without semantics’ may be oversimplified.

## Introduction

Word learning is a fundamental component of language learning and happens across life [1–4]. Lexical knowledge is a complex construct comprising different levels of linguistic representation [5,6]. For example, in our mental lexicon, word-forms (sequences of phonemes) have associated meanings (semantics), orthographic representations, and both morphological and syntactic features. In this study, we focus on a ‘proto-lexicon’—-a memory store of word-forms without accompanying meanings [7–10]—which is an important part of the language learning process. We are interested in how the proto-lexicon assists learning meanings of words in an experimental setting. While past work has examined the learning of new meanings for words that are already stored in a mental lexicon with different meanings (e.g., [11–13] for children, [14,15] for adults), there has been little discussion of the learning of meanings for words in a proto-lexicon.

Previous studies show that non-Māori-speaking New Zealanders and non-Spanish-speaking Californians and Texans have impressive implicit proto-lexical knowledge of their respective ambient languages, acquired through regular passive exposure [16–18]. For example, incidental exposure to Māori allows non-Māori-speaking New Zealanders (NMS) to develop a proto-lexicon containing over a thousand words or word parts [16], while their active vocabulary consists of only around 70 words [19]. This proto-lexicon provides phonotactic and morphological knowledge [17,20]. Furthermore, Mattingley et al. [21] shows that having a proto-lexicon gives learners a head-start in explicit learning of word meanings in an introductory language course. However, it is not yet clear how this proto-lexicon can be beneficial for learning word meanings in an experimental setting without any contextual cues, nor what kind of role is played by knowledge contained in the adult proto-lexicon in learning the meaning of words. We expect that learning the form-meaning pairing of a word would be easier for someone who already has knowledge of the form (via a proto-lexicon) and needs only attach meaning to it than for someone who has knowledge of neither form nor meaning and needs to learn them both.

This study primarily focuses on proto-lexical knowledge attained in a natural language situation. It tests the extent to which a Māori proto-lexicon facilitates learning Māori words, by teaching New Zealanders and non-New Zealanders Māori words paired with pseudo-meanings in short-term word learning tasks. Studying the role of proto-lexical knowledge provides important insights for theories of word learning and for the very earliest stages of language acquisition in adults.

## Background

### Te reo Māori

Te reo Māori is a Polynesian language and is the language of the indigenous people of New Zealand. Although the language has become endangered as the result of colonization, there are substantial revitalization initiatives. Māori is an official language and New Zealanders encounter Māori words and phrases regularly in media and public ceremonies. However, Māori is not a compulsory subject at school and only 4.3% of the population are able to hold a conversation about everyday things in Māori [22]. English is the most common language, but most New Zealanders are familiar with a small number of basic Māori vocabulary items that have been increasingly integrated into New Zealand English over time [19,23]. The most recent and second wave of borrowing from Māori into English brought in more words from the domains of Māori society and culture [24,25]. Although the Māori vocabulary knowledge of NMS varies depending on the different ways of performing a word definition task, the average NMS is likely to have an active Māori vocabulary of fewer than 100 words [19,23].

Māori has a relatively small phonology and a transparent spelling system [26]. It has 10 consonantal phonemes /p, t, k, m, n, ŋ, w, f, r, h/, which are written <p, t, k, m, n, ng, w, wh, r, h>. The five vowels /i, e, a, o, u/ correspond to the letters <i, e, a, o, u>. These five vowels also have long forms, which are usually indicated orthographically with a macron over the vowel. With regard to categorical Māori phonotactics, all syllables are open and onsets are empty or consist of any one of the consonants [27].

Māori, like other Polynesian languages, has limited inflectional morphology aside from the passive construction [26]. Derivation is a key morphological process, particularly through reduplication, compounding, and affixation, with several regular affixes used to modify word meaning [26].

### Early stages of word learning

Lexical knowledge generally involves sound structure, word-form and word meaning [5,6]. The proto-lexicon can be understood as an initial, form-based repository of word-like units that infants acquire before these forms are meaningfully linked to semantics, representing a foundational stage within the continuum of lexical knowledge [28,29]. This proto-lexical stage supports later mapping between word-form and meaning, acting as a crucial bridge from pre-lexical representations to fully specified lexical entries.

By around 9 months, infants demonstrate sensitivity to permissible sound sequences in their native languages and the frequency of occurrence of statistically recurrent sound sequences in speech [30–35]. This pre-lexical knowledge enables infants to segment the speech stream and gradually build a proto-lexicon of remembered word-forms without detailed semantic content [33,36,37]. By 11 months, infants have mental representations of familiar word-forms acquired from everyday experience, allowing recognition of these forms even when pronunciation varies (e.g., misstressing) [38]. Importantly, this proto-lexicon develops implicitly and receptively, prior to explicit learning of word meanings or orthographic knowledge.

Research suggests that statistical learning of sound sequences is crucial in early word learning. Exposure to sound sequences with strong internal structures (i.e., high transitional probabilities) facilitates word learning by mapping meanings to segmented words [39,40]. Adults are also sensitive to such conditional statistics in languages [41], and this sensitivity is stable across the lifespan [42–44].

Phonotactic probability also influences lexical acquisition, with studies showing that school-aged children learn nonword and picture pairings better when the pairs include nonwords with high phonotactic probability [45]. Taken together, these findings emphasize the importance of statistical learning and phonotactic regularity in early language acquisition.

### Previous work on building a Māori proto-lexicon

People who grow up in New Zealand are exposed to Māori throughout their lives. Computational modeling of a previous study has demonstrated that NMS have a Māori proto-lexicon of more than 1500 words or word-parts [16]. This proto-lexicon enables NMS to distinguish real Māori words from phonotactically matched Māori nonwords, and to accurately rate the gradient wellformedness of Māori nonwords [16,17]. In addition, NMS utilize their phonotactic knowledge as a proxy for rating how likely an item is to be a real Māori word if they do not already know it, with the effect being pronounced in nonwords and lower-frequency real words.

Proto-lexical representations refer to stored word-forms without associated meanings, while phonotactic knowledge consists of gradient phonotactic probabilities in permissible sound sequences rather than categorical phonotactic variations (i.e., phonology). The proto-lexicon and phonotactic knowledge represent distinct types of linguistic information, yet they are best understood as interconnected. Phonotactic knowledge is often assumed to arise from generalizations over stored words in the mental lexicon [46], and, similarly, early phonotactic knowledge in language acquisition is thought to emerge from generalizations over forms in the proto-lexicon [8,10].

Panther et al. [17] demonstrated that phonotactic generalizations emerge from the structure and distribution of stored forms in the proto-lexicon. Participants with a larger proto-lexicon—as indicated by their ability to distinguish real Māori words from similar nonwords—exhibited greater sensitivity to phonotactic patterns when judging the wellformedness of Māori nonwords. Together, these findings support the view that proto-lexical representations and phonotactic knowledge are not completely separate systems, but rather two interrelated aspects. Phonotactic knowledge is a generalization over (proto)lexical forms.

Crucially, this construct differs from a general familiarity with a language’s phonological grammar, which is typically conceptualized as an abstract rule-based system acquired through broad linguistic exposure (e.g., [47–49]). In contrast, proto-lexically derived phonotactic knowledge is statistical knowledge about the probabilities of sound sequences, emerging from exposure to specific word-forms, even in the absence of semantic content. This aligns with statistical learning accounts, which propose that linguistic knowledge arises from tracking the frequency and distribution of patterns in the input, rather than from abstract rule induction alone.

Subsequent studies have demonstrated that NMS have further well-developed implicit knowledge of Māori; NMS have some syntactic knowledge [50] and they are also able to morphologically segment Māori words in a similar way to fluent Māori language speakers [20,51]. Further studies have revealed that incidental exposure to Māori continues to lead to implicit learning and growing the proto-lexicon throughout the adult lifespan; NMS who have more exposure during adulthood have more extensive knowledge [52].

Although NMS have more than 1500 words or word-parts stored in their proto-lexicon [16], they can explicitly define the meanings of only around 70 words, indicating that most of their knowledge is limited to form without accessible semantic content words [19]. Nonetheless, they demonstrate sensitivity to various aspects of Māori knowledge such as morphological segmentation [20,51], which may reflect proto-lexical knowledge, and syntactic regularities [50], which likely emerge from broader implicit exposure. It is very likely that NMS are unaware of the meaning of most words in their proto-lexicon. However, a proto-lexicon may aid in the learning of form-meaning pairs, through providing forms to which meanings can be attached. Mattingley et al. [21] investigated the role of the proto-lexicon in a real life language learning study, showing that the proto-lexicon can be activated to facilitate overt language learning. Adult students with larger proto-lexicons have a learning advantage when formally learning the meanings of Māori words in a formal education environment.

While proto-lexical representations are primarily form-based, emerging evidence suggests that they may nonetheless engage in graded or partial semantic associations. This aligns with research showing that sublexical or distributional features can influence semantic processing, even for unfamiliar word-forms or nonwords (e.g., [53,54]). Such findings suggest that the boundary between form-based proto-lexical knowledge and semantic knowledge may be more permeable than previously assumed—–implying a continuum, rather than a strict dichotomy.

### The present study

Taken together, the above studies show that a proto-lexicon can be built through both childhood and adulthood exposure to a target language in a natural language situation. This knowledge remains largely implicit, as NMS typically possess only a small explicit Māori vocabulary. Nevertheless, having a proto-lexicon appears benefit learners when acquiring Māori word meanings in formal education settings.

However, we do not yet know whether there is a relationship between a proto-lexicon and word learning in controlled experimental settings. In Mattingley et al. [21], students’ word learning ability in Māori was assessed using words from their course materials. Some words were explicitly learned as required words in the course, while others were encountered incidentally through course exposure. Therefore, we do not know how robust a proto-lexicon is for word learning in a controlled environment.

Additionally, it is possible that the Māori proto-lexicon comprises multiple knowledge phases or stages of lexical knowledge. For example, Dale [55] proposes four developmental stages of word knowledge. Each stage is characterized by learners’ comprehension and use of words. The four stages are: (1) having never seen the word before, (2) knowing there is such a word but not knowing what it means, (3) having a vague contextual placing of the word, (4) knowing the word and remembering it. In this framework, the second and third stages are most relevant to the present study. Specifically, the second stage can be seen as equivalent to the concept of a proto-lexicon.

Building on this framework, the present study asks: to what degree is the Māori word learning ability of NMS, who have a Māori proto-lexicon (i.e., the second stage), greater than that of people who do not have a Māori proto-lexicon (i.e., the first stage)? When NMS are compared to people who do not have a Māori proto-lexicon, we would expect that implicit knowledge of NMS gives them an advantage in attaching meaning to words. We hypothesize based on the developmental stages of word knowledge [55] that most words in the Māori proto-lexicon are at the second stage and therefore would show an advantage over nonwords at the first stage in the learning of meaning for NMS. On the other hand, non-New Zealanders would show no differences in the way they learn the meanings of real words and nonwords. Accordingly, the present study set out to address the following research question:

**Primary RQ:**

Is there a relationship between NMS’ proto-lexical knowledge and word learning in experimental settings? That is, does a proto-lexicon facilitate the attachment of meanings to word-forms?

In order to explore this question, we explore a range of related questions. One of them is the degree to which a forced-choice definition task gives the same answer about the degree of semantic knowledge as a free-response definition task. Another is the degree to which words that are not well-defined in a forced-choice task are actually robustly present in the proto-lexicon. We refer to both the forced-choice and free-response formats as word definition tasks throughout.

To address our primary research question, we conducted two separate web-based experiments. First, in Experiment 1, we ran a forced-choice definition task to identify Māori words with no robust associated meanings among NMS. These words served as stimuli for Experiment 2, where we directly answered our primary research question. In Experiment 2, we conducted short-term word learning tasks to test whether a non-semantic Māori proto-lexicon benefits learning word meanings. We taught pseudo-meanings for the selected words to NMS and non-New Zealanders living in the USA. By comparing performance across these groups, we explored how a proto-lexicon influences word learning in an experimental context.

Our findings show that NMS perform better at the task than non-New Zealanders, but that there is no difference in their performance with words and real words. To explore a potential explanation for this, in Experiment 3, we conducted web-based word identification and wellformedness rating tasks with the same NMS participants from Experiment 2. This experiment assesses the extent to which these participants definitely have a Māori proto-lexicon and related implicit knowledge, while also exploring whether this proto-lexicon includes the specific Māori words examined in Experiment 2.

## General methods

This study is based on three web-based experiments that we conducted, as described in the previous section, alongside distinct datasets from previous studies used for comparison. All our experiments in this article were conducted individually via the web, at a time convenient for participants and in a location of their choice. Participants were first presented with instructions and consent forms. All experiments were carried out with full ethical clearance from the Human Research Ethics Committee at the University of Canterbury (2017/90, 2022/10/LR-PS). Participation was entirely voluntary. Participants could choose to receive a $10 online gift voucher in Experiment 1. In Experiments 2 and 3, compensation was provided according to Prolific regulations.

Although the stimuli in Experiment 3 were presented in written form, the transparent orthographic-to-phonological mapping in Māori ensures that the written stimuli reflect the language’s phonological structure. This allowed to us to assess participants’ sensitivity to gradient phonotactic probabilities—probabilistic patterns of permissible sound sequences in Māori. These patterns reflect implicit statistical learning rather than categorical phonological rules. Osborne et al. [Unpublished] demonstrated that NMS exhibit phonotactic sensitivity across both spoken and written modalities, supporting the cross-modal nature of this gradient phonotactic knowledge.

Participant exclusion criteria, including non-qualifying demographics, and statistical models were determined prior to data analysis based on established procedures from previous studies (see Technical Supplement for details S1 File)

Table 1 summarizes our experiments and the datasets used in this study. In the case of Panther et al. (2023), stimuli from both the wellformedness rating and word identification tasks were used in our experiments; however, only the word identification data were analysed in relation to Experiment 2.

**Table 1:** Datasets and task methods summary.

Study/Experiment	Year	Task/Method
Oh et al.	2023	Free-response definition task
Experiment 1	This study	Forced-choice definition task
Experiment 2	This study	Word learning task
Panther et al.	2023	Word identification task
Experiment 3	This study	Wellformedness rating task; Word identification task

## Experiment 1: Definition task

In Experiment 1, we conducted a two-task, web-based experiment through a custom browser interface between January 22-26, 2022. The session consisted of a forced-choice definition task and a word splitting task. In this paper, we focus on the forced-choice definition task. Although participants also completed the word splitting task as part of the study design, it is not reported here because it addresses a different research question.

The definition task had two purposes. The primary purpose was to identify Māori words for which NMS cannot readily recognize the definitions, to use these words as stimuli in the word learning task of Experiment 2. In our word learning task, we wanted to use words that participants did not have robust existing meanings for. We were also interested in the fact that the different ways of performing a definition task might show different degrees of strength of knowledge in relation to various developmental stages. Thus, the secondary purpose was to compare our findings with a previous free-response definition task assessing the size of the active vocabulary [19].

### Participants

Participants, recruited via paid Facebook advertisements, were 68 adults. Among those people who completed the experiment, we excluded 13 participants with non-qualifying demographics (see Technical Supplement for details S1 File). After excluding these participants, 55 adult native speakers of New Zealand English remained (45 female). All participants were aged between 18 and 60 years and did not have sufficient proficiency in Māori to hold a basic conversation in Māori, in line with the inclusion criteria. According to the demographic survey, 87.3% were monolingual, while 12.7% reported being able to speak one additional language, such as Japanese, French, or Spanish.

### Materials

Stimuli in this task were real Māori words, drawn from the material in Panther et al. [17]. Panther et al.’s original material consisted of 521 Māori words and 521 Māori-like nonwords. All stimuli consisted of three to six phonemes and did not contain long vowels. Words were categorized according to frequency in spoken Māori (high, mid, low) based on the MAONZE corpus [56] and the Māori Broadcast Corpus [Boyce, Unpublished].

Oh et al. [16] demonstrated that the proto-lexicon of NMS is composed primarily of morphs, which are frequent phonological segments derived from ambient speech, rather than full words or semantically meaningful units. To measure participants’ phonotactic knowledge, we utilized the SRI Language Modeling Toolkit (SRILM) [57] to assign phonotactic scores to each word. These scores were based on morphs identified from fluent speakers’ segmentation of dictionary lemmas (see [16] and their Detailed Materials and Methods Supplement for further details). The morph dataset consists of unique types only, with each morph represented once. The phonotactic scores reflect length-normalized log-probabilities (base 10) calculated from the frequencies of phoneme trigrams. The scores ranged from –1.2 to –0.60, corresponding to average conditional probabilities of the third phoneme in a triphone (given the preceding two phonemes as context) that range from 0.063 ( = 10^−1.2^) to 0.251 ( = 10^−0.6^). A high phonotactic score indicates that the local sound sequences that make up the stimulus are, on average, quite typical or common in Māori words.

Each word was also assigned a neighborhood occupancy rate: the proportion of its potential phonological neighbors (i.e., phonotactically legal forms that are a Levenshtein edit distance of one from it) that correspond to a real Māori word. The neighborhood occupancy rate can be understood as a normalized version of phonological neighborhood density (i.e., the number of real words whose phonological forms are a Levenshtein edit distance of one from the stimulus), reflecting the probability that distortion in the perception of a single phoneme would result in a real word. In general, the longer a stimulus, the lower its neighborhood occupancy rate. A high neighborhood occupancy rate indicates that it globally resembles many Māori words. See Panther et al. [17] for further details.

Among 521 Māori words, we selected a fixed set of 146 words from the high- and mid-frequency categories (high; *n*  =  53, mid; *n*  =  93) and obtained a definition for each word from a dictionary. For each participant, all 146 words were paired up with all 146 definitions, such that a random subset of 73 words were paired with their correct (matched) definition and the remaining 73 words were paired with an incorrect (mismatched) definition corresponding to a different word. Stimulus characteristics were as follows: lengths (*M* = 4.35, *SD* = 0.92), phonotactic scores (*M* = −0.81, *SD* = 0.1), and neighborhood occupancy rates (*M* = 0.1, *SD* = 0.06).

### Procedure

In this experiment, we tested adult NMS’ Māori word knowledge by a web-based binary forced-choice task. For each trial, participants saw a stimulus word and a definition in the middle of the screen. The definition could be either a correct (matched) definition or an incorrect (mismatched) definition. Participants were asked to answer whether the presented word-definition pair was ‘good’ or ‘not good’. After the participant clicked one of the options and the ‘Next’ button, the next stimulus was presented.

Before starting the task, participants were instructed to provide their best guess without relying on a dictionary or help from others, in order to obtain each individual’s actual knowledge of Māori words. After the task, participants also completed a post–questionnaire which included 27 demographic questions. The experimental session lasted less than 30 minutes in total.

To evaluate whether participants relied on external aids, we examined their total task duration and median reaction times. If a participant’s median reaction time exceeded two standard deviations above the group median, we also analysed their accuracy. Two participants exceeded this threshold, and their accuracy was near chance level (51% and 56%), suggesting they were not using external aids.

### Results

#### The word list for Experiment 2.

To determine overall accuracy of definitions for each participant and each word, we calculate a d-prime [58] score which is a measure of participants’ response sensitivity for the correctness of pairings between words and definitions. Participants with higher values of d-prime more accurately discriminated between matched and mismatched word-definition pairs, and words with higher values of d-prime were more accurately identified by participants as matched or mismatched to their paired definitions.

In terms of stimulus words, the rate of correct responses varied among words, ranging between 33% for *kuhu* ‘to enter’ and 93% for *whenua* ‘land’. The participants selected correct options with more than 50% chance for 109 words out of 146 words (75%), which indicates that they can identify word meanings in Māori words for a substantial subset of the words.

A d-prime score for each stimulus ranged between –0.82 and 3.46. After initial data exploration, we selected stimuli by setting cutoff points for maximum d-prime and minimum phonotactic score. Stimuli were required to have a d-prime less than or equal to 0.3, indicating that participants were not consistently able to match them to their definitions, and a phonotactic score greater than or equal to –0.89 (against an overall range of –1.2 to –0.60), indicating that they were not composed of atypical phoneme sequences. Based on these criteria, we selected 48 words for the word learning task (Experiment 2). Because of their low d-prime scores, these words are less likely to be associated with semantic knowledge, and because their phonotactic scores are drawn from a high and narrow range (–0.868 to –0.645), their forms are all sufficiently typical for Māori in a similar way, meaning there should not be asymmetries in the effect of phonotactic regularity on attaching meanings to word-forms.

For creating a word list, we did not remove any participants based on participants’ d-prime score because the list would be based on how widely the words were known regardless of participants’ ability.

#### Comparing results in two different definition tasks (Descriptive statistics).

For the secondary phase of this study, we directly compare our findings with the previous definition task assessing the size of the active vocabulary [19]. Oh et al. [19] used a free-response definition task to evaluate the explicit knowledge of 132 Māori words by 123 NMS. The average New Zealander can define about 70 words. We focus on the comparison between the free-response definition task and the forced-choice definition task. Both definition tasks measure NMS’ receptive knowledge of form and meaning and the varying degrees at which each word is known, but determine different degrees of strength of knowledge [59].

To begin, five participants who responded to more than 95% of trials in the same way (e.g., “not good”) in our forced-choice definition task dataset were removed from the data analysis. After the removal process, analyses were carried out on 7300 observations of stimulus words with 50 participants. The mean accuracy was nearly identical when summarized by item and by participant (*M* = 0.59 for both). However, the variability differed: accuracy varied more across items (SD=0.12) than across participants (SD=0.06). This suggests that item difficulty contributed more to performance differences than individual differences among participants, who were relatively consistent in their accuracy.

For 12 words overlapping between the current study and Oh et al.’s [19], we report the extent to which the mean accuracy of each word is correlated with each other from different tasks. In order to compare directly between two different definition tasks, we adjusted the accuracy scores of each item in the free-response definition task by 0.5*free-response definition accuracy + 0.5. This is because participants would be expected to get it right 50% of the time just by random guessing in the forced-choice definition task.

**Fig 1:**
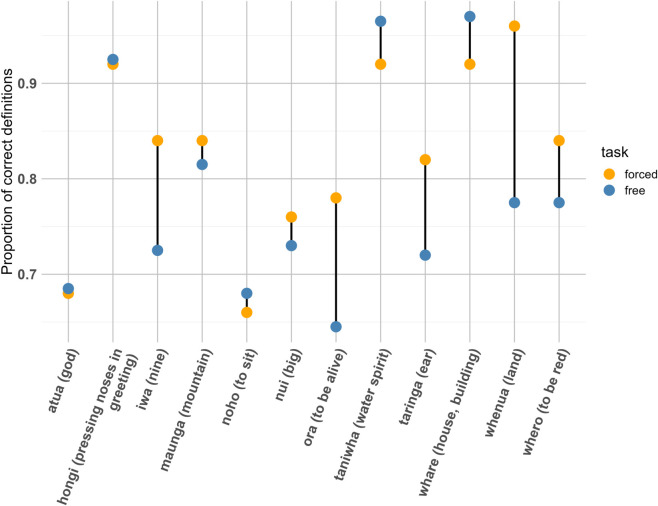
The relationship between two tasks for the proportion of correct responses for each word. Definitions of words are in the brackets.

Fig 1 shows the proportion of correct responses per word in both definition tasks. Half of words have higher accuracy in the forced-choice definition task, by up to 10% compared with free-response definition accuracy. In a few cases, the two situations give similar accuracy. The other three words (*hongi* ‘pressing noses in greeting’, *taniwha* ‘water spirit’, *whare* ‘house, building’) that were defined accurately more than 90% of the time in both the tasks are strongly related to the Māori culture. This might be attributed to a ‘second wave’ of borrowings from te reo Māori driven by Māori speakers in which more Māori words in the domains of Māori society and culture have been integrated into New Zealand English [24,25].

On the other hand, the word *whenua* ‘land’ which is one of the most frequent Māori words in New Zealand contexts shows the near perfect accuracy in the forced-task but about 15% lower in the free-response definition task. We can see the similar trend from the word *ora* ‘to be alive’. The word appears in *Kia ora* which is a Māori-language greeting which has been integrated into New Zealand English. These words are more likely at stage 3 (i.e., a vague contextual placing of the word) in Dale’s [55] four developmental stages of word knowledge. In the free-response definition task [19], even if participants provided the meanings of partially known words (for example, knowing that *iwa* was a number, but not that it meant ‘nine’), these responses were regarded as incorrect definitions. It seems that presenting a definition with a word could help participants retrieve the word from their memory or infer the definition with less mental effort. Without any cues, participants need to retrieve and recall their memory for knowing words with more mental effort.

The forced-choice accuracy is generally higher. However, there is not a huge difference in accuracy between the two definition tasks after we adjusted the accuracy scores of each item in the free-response definition task in order to account for random guessing in the forced-choice definition task.

### Summary of Experiment 1

In Experiment 1, the participants could identify word meanings in Māori words for a substantial subset of the words. Recalling Dale’s [55] development stages of word knowledge in the Present Study section, it is likely that the second stage and the third stage in this measurement are closely related to our findings. Although it has been assumed that most words in the proto-lexicon were at the second stage (i.e., knowing there is such a word but not knowing what it means), it is clear that for NMS, there are some words that are actually at the third stage (i.e., a vague contextual placing of the word).

In terms of definitions, most words had higher accuracy in the forced-choice case than in a previous study using a free-response definition task, whereas words that are related to Māori culture were associated with their meanings accurately 90% of the time for both definition tasks. The findings are consistent with previous studies using corpora, which shows that in the second wave of borrowing from Māori into English, words related to Māori society and culture have increased in token frequency [60] and Pākehā (European or white New Zealander) are more likely to use loanwords that refer to Māori culture [61].

## Experiment 2: Learning meanings of words

Experiment 2 is designed to address our primary research question. We examine whether there is a relationship between NMS’ proto-lexical knowledge and word learning in experimental settings. Our research question asks how a proto-lexicon facilitates the attachment of meanings to word-forms in Māori. We conducted a web-based experiment through a custom browser interface via Prolific (www.prolific.com) between September 18-19, 2022 for non-New Zealanders and from November 28, 2022 to January 9, 2023 for NMS. Experiment 2 was a word learning experiment which was divided into three phases. In the learning phase, participants were asked to try their best to learn new words. In the two test phases, participants were tested on their word learning. The participants completed a post-questionnaire after the tasks. The experimental session lasted less than 20 minutes in total. The details of each task are given below.

### Participants

Experiment 2 involved two groups of adult participants: (a) NMS and (b) non-New Zealanders living in the USA. Among people who completed the experiment, we excluded participants who did not pay attention to the tasks and did not fulfill our demographic conditions (see Technical Supplement for details S1 File). After removing unusable participants, there were 137 participants in total: 70 NMS (36 female) and 67 US participants (31 female). All participants were native speakers of English and aged between 18 and 60, and have never studied linguistics at a college, university, or community college. They were not able to hold a basic conversation in te reo Māori. They have not lived outside their countries for any period of longer than a year since they were aged seven. According to the demographic survey, among NMS participants, 81.4% were monolingual, while 2.9% reported speaking two additional languages and 15.7% one additional language. For US participants, 82.1% were monolingual, 4.5% reported speaking two additional languages, and 13.4% one additional language. Examples of additional languages include Japanese, French, and Spanish.

### Materials

#### Word stimuli.

As mentioned in the Materials section of Experiment 1, we selected 146 words from high and mid frequency categories from an original stimulus set [17] and conducted a definition experiment with participants. We filtered out words that participants were able to identify easily in the definition experiment. For the word learning experiment, we selected 48 words with low d-prime scores from the definition experiment that spanned a narrow range of phonotactic scores, along with their phonotactically matched nonwords (words/nonwords: phonotactic score range = -0.87 to -0.65). We focused on this narrow range of phonotactic scores because phonotactic regularity promotes attaching meanings to word-forms (e.g., [45]), as discussed earlier. This approach allowed us to disentangle effects of form-based (proto-lexical) knowledge on word learning from phonotactic knowledge. The word and nonword stimuli were closely matched on key lexical characteristics. Both stimulus types had identical average lengths (*M* = 4.54, *SD* = 0.74), phonotactic scores (*M* = −0.77, *SD* = 0.06), and neighborhood occupancy rates (*M* = 0.09, *SD* = 0.06). This ensured that any observed effects could not be attributed to differences in these properties. Table 2 presents example word/nonword pairs with their phonotactic scores.

**Table 2:** Example word–nonword pairs from Experiment 2 with phonotactic scores.

Word	Score	Matched Nonword	Score
tahaki	–0.645	tariki	–0.651
wehe	–0.868	mehe	–0.861

#### Picture stimuli.

In order to obtain a range of objects for which NMS are unlikely to know the true Māori term, we selected 16 objects from the online database of the International Picture Naming Project [62]. There were several criteria to control effects of picture stimuli. The English language was selected to search for picture items. All of the pictures were objects from a range of semantic categories including: animals, small and large artifacts, body parts, vehicles, clothing, objects in nature, people. All of the words consisted of one syllable in English (e.g., bat, bridge). The number of alternative names for the object (in English) was three or fewer. Pictures were black line-drawings.

#### Stimulus set.

In the experiment, there were three experimental tasks (learning phase, post-test phase 1, post-test phase 2) as well as three repetitions within each task. There were 16 picture stimuli, 48 real words and 48 nonwords as mentioned in the previous section. Each participant must add meaning to 16 word-form stimuli. Therefore, we prepared 36 experiment configurations containing stimuli paired with pictures. There were six random orders of the pictures in the experiment. We also created six random samples of eight words and eight nonwords. Each stimulus appears only once across the 6 word stimulus samples (i.e., there are no repeat stimuli across the samples). A total of 36 experimental configurations were used.

For the purpose of matching stimuli for the experimental tasks, a procedure was followed to identify: (i) another stimulus that has an ‘incorrect’ image for the learning phase (task 1); (ii) an image that is the incorrect ‘word’ for post-test phase 1 (task 2); (iii) an ‘incorrect’ ‘word’-image pairing for post-test phase 2 (task 3). For task 1, stimuli were randomly paired together, and were a unique match to each other, for each experimental configuration. For task 2, the same procedure was followed, although for each stimulus, its task 1 pair was excluded. For task 3, the stimuli were split in half: half of the stimuli retained their ‘correct’ ‘word’-image pairing, while the other half of the stimuli were matched with another stimulus within that group (which could not also be the task 1 or task 2 pairing), and their ‘word’-image pairing was swapped, and these formed the ‘incorrect’ matching group. Correct response would be shown on the left/right side.

### Procedure

Participants completed three tasks that were implemented and presented to participants using OpenSesame and OSweb [63,64]. Before starting the experiment, all participants were presented with a page containing information about the experiment, and in the study description we mentioned that we would be using an indigenous language of New Zealand, Māori, as the basis of this research.

The participants were presented with instructions on the computer screen prior to each task. At first, participants were instructed that “You are stranded on an island and you don’t know the language spoken on this island. Your task is to learn the names of objects in the language of the island.” Participants were told that if their decision took too long, a new item would be shown. They were provided with an instruction to not worry about making a lot of mistakes and just to try and learn as much as they could throughout the task. After reading each instruction, participants needed to press a specific key to start their task.

The experiment was divided into three tasks. Each task had three repetitions in different stimulus order. The purpose of task 1 (learning phase) was to learn the names of objects in the language of the experimental setting. Each trial started with a fixation dot presented in the center of the screen for 500 ms (the same applied to the following tasks). Participants then saw a picture in the middle of the screen. After a further 500 ms delay, two words appeared, one of which was the correct name of the pictured object. One word was presented on the left side of the picture and the other on the right side. Participants were instructed to press the ‘z’ key if they thought the picture matched the word on the left side of the screen and to press the ‘m’ key if they thought the picture matched the word on the right side of the screen. If a participant responded incorrectly, they would see a red cross in the middle of the screen; if they responded correctly, they would see a green tick. On each trial, the stimuli were presented together for (up to) 5000 ms; if a participant did not respond within this time limit, a new item would be shown without any feedback.

In task 2 (post-test phase 1), on each trial, participants saw a word in the middle of the screen, then after 500 ms of delay, two pictures appeared. One picture was presented on the left side of the word and the other on the right side. The task was to decide which picture matched the word. If participants thought the left picture matched the word, then they pressed the ‘z’ key. If they thought the right picture matched the word, then they pressed the ‘m’ key. No feedback was given for this phase. On each trial, the stimuli were presented together for 5000 ms or until the participant responded, at which point the fixation for the next trial began.

In task 3 (post-test phase 2), for each trial, participants saw a word above a picture. The word may or may not be the correct name for the picture. After 500 ms of delay, two words appeared: ‘correct’ and ‘incorrect’. The word “incorrect” was presented on the left side of the stimuli and the other word “correct” on the right side. The task was to decide whether the word and the picture was either a correct or incorrect pair. If participants thought the pair was correct, they pressed the ‘m’ key. If participants thought the pair was incorrect, they pressed the ‘z’ key. No feedback was given for this phase. On each trial, the stimuli were presented together for 5000 ms or until the participant responded, at which point the fixation for the next trial began.

After the third task, participants answered a demographic questionnaire using a customized open source survey software tool, Lime Surveys [65]. The experiment took less than 20 minutes to complete. At the end of the experiment, a debriefing statement appeared to explain that some of the words participants learnt were not real Māori words and the meanings they learnt for the real Māori words were not the real meanings.

### Results

In order to assess the ability of NMS and non-New Zealanders to learn the word meanings, their responses were coded as accurate or inaccurate. For tasks 1 and 2, responses were coded as accurate if the participant chose the correct word/picture; for task 3, responses were coded as accurate if the participant correctly indicated whether or not the word-picture pair were a match. Analyses were carried out on 19,728 observations of stimulus words (48 stimuli × 3 tasks × 137 participants) with mixed effects logistic regression, using the glmer function in the lme4 library of R [66,67] along with the bobyqa optimizer in R.

Table 3 presents the mean accuracy (in percentages) and standard deviations for each combination of participant group (NZ, US), stimulus type (word, nonword), and task (1–3). Sample sizes (N) indicate the total number of observations included in each condition. Accuracy rates were generally consistent across groups, with some variability observed across tasks and stimulus types. Notably, except for Task 1, the NZ group tended to perform better than the US group. This summary provides a descriptive context for interpreting subsequent statistical analyses.

**Table 3:** Mean accuracy (percentage) with standard deviation and sample size (N) by language group, stimulus type, and task.

Group	Stimulus Type	Task	Accuracy (Mean ± SD, %)	N
NZ	Nonword	1	59.0 ± 49.2	1680
US	Nonword	1	57.6 ± 49.4	1608
NZ	Nonword	2	76.5 ± 42.4	1680
US	Nonword	2	69.0 ± 46.3	1608
NZ	Nonword	3	75.7 ± 42.9	1680
US	Nonword	3	71.6 ± 45.1	1608
NZ	Word	1	60.5 ± 48.9	1680
US	Word	1	60.2 ± 49.0	1608
NZ	Word	2	79.0 ± 40.7	1680
US	Word	2	71.7 ± 45.1	1608
NZ	Word	3	78.5 ± 41.1	1680
US	Word	3	73.5 ± 44.1	1608

The dependent variable was accuracy, defined above. The fixed effects considered were participant group (sum-coded, US[1], NZ[–1]), word type (treatment-coded, reference: nonword), and experimental task (Helmert-coded) as test predictors. We added another factor, education (treatment-coded, reference: high school) as a control predictor, which expects that individuals with different education backgrounds might have different learning capabilities in general. Our primary interest is in the interaction between the test predictors. Our consideration of interactions with experimental task is guided by the two comparisons formed by the use of Helmert coding. The first comparison (task 1 vs. task 2-3) would indicate the difference between the learning phase and two test phases. The second comparison (task 2 vs. task 3) would indicate the difference between two tests.

We hypothesized that because the real words in the experiment are part of NMS’ proto-lexicon, NMS would learn the meanings of the real words more accurately than non-New Zealanders. We also expected that NMS would learn the meanings of the real words more accurately than the meanings of the nonwords but non-New Zealanders would show no differences in the way they learn the meanings of real words and nonwords. We started the stepwise model fitting process with interactions between all test predictors, as in the previous analysis(see [21]) described in detail in the supplementary materials. An initial model contained a three-way interaction between group, type and task and education is a simple effect; glmer (Accuracy ~ Group * Type * Task + Education + (1|PID) + (1|Word). The initial fitting procedure applies to fixed effects only, and later stages add intercepts and slopes. In the final model, stimulus type was added as a by-participant slope, and group and task were added as by-word slopes. For the interpretation of significant interactions, we conducted post-hoc estimated marginal means (EMM) tests using the R package emmeans [68]. The resulting model is shown in Table 4.

**Table 4:** Model results for Experiment 2 (learning accuracy).

Predictors	Estimate	Std. Error	*z*	*p*
(Intercept)	0.932	0.074	12.673	<0.001
Group [US]	–0.138	0.053	–2.576	0.010
Task [1vs.2-3]	–0.854	0.065	–13.038	<0.001
Task [2vs.3]	–0.177	0.097	–1.832	0.067
Type = word	0.109	0.067	1.634	0.102
Group [US] × Task [1vs.2-3]	0.170	0.034	4.952	<0.001
Group [US] × Task [2vs.3]	–0.075	0.043	–1.724	0.085

**Fig 2:**
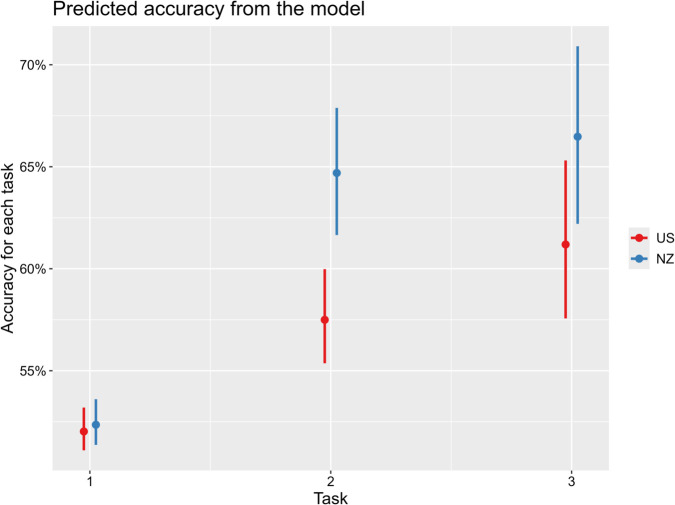
Interaction plot between group and task from the final model. Estimated 95% confidence intervals on predictors. Bias correction was applied for this plot. The x-axis shows each task in the experiment. The y-axis shows the predicted portion of accurate responses.

We did not observe a significant interaction between stimulus type and group. Results of the model showed a significant main effect of group, indicating that non-New Zealanders performance was lower than the grand mean of two groups. The effect of task for the first comparison between the learning phase and the test phases was also detected, which indicates that participants gave lower-accuracy responses in the learning task, when they were first presented with words and images and had to learn a mapping between them by making guesses and incorporating feedback, than in the test tasks, when they already had (partial) knowledge of the mapping based on feedback from the learning task. There was also a significant interaction between group and task for the first comparison (task 1 vs. task 2 and 3), as suggested by Fig 2. An EMM test confirms that the effect of group is significant across tasks and confirms that two groups performed similarly in task 1 (task  =  1: NZ - US; β= 0.049, z  =  0.428, *p* = .669), but NMS learned meanings of words better in task 2 and 3 (task  =  2: NZ - US; β= 0.464, z  =  3.938, *p* = < .001, task  =  3: NZ - US, β= 0.314, z  =  2.619, p=< .01).

### Summary of Experiment 2

Contrary to our prediction, we did not observe an interaction between stimulus type and group. That is, although NMS generally learned new meanings more accurately than non-New Zealanders, this advantage was not focused on their learning of meanings for words that were assumed to already exist in their proto-lexicon. Rather, it appears that each group learned the meanings for nonwords as well as they did for words, and NMS were better than non-New Zealanders at learning the meanings for both. Consequently, our prediction that NMS would learn the meanings of real words easier than nonwords was not supported by the experimental evidence.

It does, however, appear that NMS have an overall advantage in this task. Education level is controlled in the model, and so group differences for this variable would not be an explanation for why NMS have done better. The most likely explanation is that all of the words are very Māori-like, and so are more statistically likely for NZ-based participants than non-NZ participants. We know that phonotactic knowledge can facilitate word learning [45], and we know that New Zealanders have extremely good phonotactic knowledge of Māori [16,17]. The results are consistent with the interpretation that this phonotactic knowledge facilitates word learning for Māori-like words.

Based on these results, we considered whether there was detectable variation in the stimuli which might cause NMS’ performance to be better than that of non-New Zealanders. Therefore, phonotactics and neighborhood effects of stimuli were examined, however, exploratory analyses showed no differences in task performance between these groups (see Technical Supplement 3.7.1 for details S1 File). This is likely to be due to the narrow range of Māori words and phonotactic scores in the stimuli.

So while there is an advantage for New Zealanders in this task, there is no evidence that this advantage is lexical. There are three potential interpretations of this lack of a lexical effect. One of them is that having a form in the proto-lexicon does not, in fact, confer an advantage for learning its meaning. This would need to be reconciled with the classroom results reported by [21]. A second possibility is that there is something about our task which obscures this advantage. For example, we are not teaching the real meanings of the words, and so it is possible that there is latent conflicting semantic knowledge which interferes with the ability to assign our pseudo-meaning to the words. Additionally, we did not very explicitly orient people to the idea that they were being presented with Māori words. The information sheet contained the phrase “we will be using an indigenous language of New Zealand, Māori, as the basis of this research” in the research description, but we are not confident that all participants read the full information sheet carefully. The separately presented instructions (which they are more likely to have read, and which were presented later), said simply “You are stranded on an island and you don’t know the language spoken on this island. Your task is to learn the names of objects in the language of the island.” It is thus very likely that they were not particularly oriented to the Māori language. The belief that they are learning some other language may thus suppress any lexical advantage that the Māori proto-lexicon might otherwise have conferred.

A final possibility is that, while past work has shown that New Zealanders have a Māori proto-lexicon, that lexicon does not happen to contain the specific words that are in our learning experiment. This is a possibility, given the fact that we explicitly selected words in our experiment which have low accuracy in a definition task.

**Fig 3:**
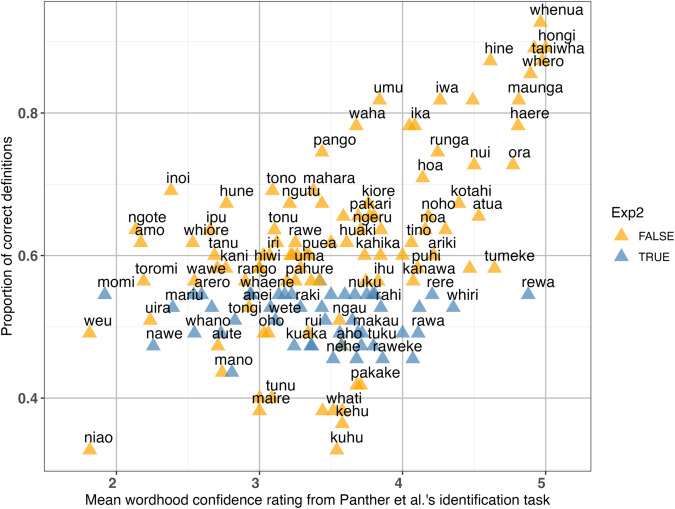
The relationship between proportion of correct definitions for each word and their mean wordhood confidence ratings from Panther et al.’s [17] identification task.

In order to explore this final possibility, we returned to our real word stimuli and compared them with Panther et al.’s [17] identification task. Fig 3 shows the proportion of correct definitions for each word in our definition task, and their relation to NMS participants’ mean wordhood confidence ratings using a 1-to-5 scale in the Panther et al.’s [17] identification task. The higher the wordhood confidence rating is, the more NMS in [17] were confident that the stimulus was a real word. We can see that there is a positive correlation. Words that were well-defined in our experiment were also well-identified in the word identification task.

The words that were ultimately selected as stimuli for Experiment 2 are highlighted in light blue. These words were accurately defined between 40 to 60% of the times in the definition task. While we selected words as stimuli for Experiment 2 in a narrow band of accuracy rate, these words spread across the identification ratings in the Panther et al.’s [17] data. Our stimuli contain some words which were not defined with greater than chance accuracy and were rated lower than three. Although we assumed all of our experimental words are in the proto-lexicon of NMS, this may not actually be the case.

In order to determine whether our experiment participants had a proto-lexicon and— particularly—whether they had these words in their proto-lexicon, we invited our Experiment 2 NMS participants back for a further experiment. We investigate the degree to which participants in Experiment 2 can accurately discriminate words from nonwords, using stimuli that contain words and nonwords used in this experiment.

## Experiment 3: Proto-lexicon in NMS

In order to assess NMS’ proto-lexical knowledge, we conducted a two-task web-based experiment through a custom in-browser interface via Prolific (www.prolific.com) from March 1, 2023 to August 2, 2023. The identification task asks participants to distinguish real words from phonotactically matched nonwords. The wellformedness rating task is designed to assess NMS’ sensitivity to gradient phonotactics. The details of each task are given below.

### Participants

Participants, recruited via Prolific, were 65 NMS who participated in Experiment 2. Among those people who completed the experiment, we excluded one participant who had little variation in responses. After removing the participant, there were 64 NMS.

### Materials

This experiment draws on a pre-existing set of stimulus materials used in [17,52], which consisted of Māori word and Māori-like nonword pairs, as described in the Materials section of Experiment 1.

For the wellformedness rating task, the pre-existing stimulus set includes 60 nonwords that were sampled from each of three phonotactic bins. We used precisely these stimuli, with no additions or removals. Stimulus characteristics were as follows: lengths (*M* = 4.67, *SD* = 0.99), phonotactic scores (*M* = −0.82, *SD* = 0.15), and neighborhood occupancy rates (*M* = 0.07, *SD* = 0.05).

For the word identification task, the pre-existing stimulus set includes 30 high-frequency and 30 mid-frequency words, each paired with a nonword of the same length and similar phonotactic score, for a total of 120 stimuli. To this set, we added the words used in Experiment 2 (if they were not already present), together with their phonotactically matched nonwords. This yielded a total of 182 stimuli (91 word-nonword pairs). For stimulus characteristics, both stimulus types had identical average lengths (*M* = 4.38, *SD* = 0.84), phonotactic scores (*M* = −0.79; nonwords: *SD* = 0.09, words: *SD* = 0.08), and neighborhood occupancy rates (*M* = 0.10, *SD* = 0.06).

For each task, each participant responded to all stimuli in random order. No stimuli were shared between the two tasks.

### Procedure

Participants completed the wellformedness task first, followed by the identification task. Task instructions were presented prior to each task. For both tasks, participants responded to orthographic stimuli, with stimuli presented one at a time. In the wellformedness task, on each trial, participants were presented with a nonword, and they were asked to rate how Māori-like it was using a scale ranging from 1 (*non Māori-like nonword*) to 5 (*highly Māori-like nonword*). After the participant chose a rating and clicked ‘Next’, the next stimulus was presented. No training was given to participants, but rating examples were given in the instructions for a highly Māori-like nonword and non-Māori-like nonword which were not actual stimulus items.

In the identification task, participants were presented with either a word or nonword from a set of real Māori words and phonotactically matched Māori nonwords. Participants were asked to rate how confident they were that the stimulus was a real word using a scale ranging from 1 (*confident that it is NOT a Māori word*) to 5 (*confident that it IS a Māori word*). Participants were instructed to give their best guess without relying on other tools (e.g., a dictionary) or help from others. After the participant chose a rating and clicked ‘Next’, the next stimulus was presented. The total experiment took less than 30 minutes.

We applied the same measure used in Experiment 1 to evaluate potential external aid usage, given the absence of time constraints in this task. Two participants exceeded the threshold for median reaction time. We compared their median reaction times between real-word and nonword trials. For one participant, the difference was negligible (0.023 seconds), and for the other, it was modest (0.509 seconds). Given the minimal differences and the robustness of the median to outliers, there is no evidence of systematic aid usage.

### Results

Consistent with previous studies [17,21], we analysed the data with mixed-effects ordinal regression using the ordinal package [69] in R [67]. For both tasks, the dependent variables were rated on a Likert scale (in the wellformedness task: wellformedness judgment ratings, in the identification task: wordhood confidence ratings). Categorical predictors were treatment-coded, and numerical predictors were centered. Random intercepts for participant and word were included in all models, with by-participant random slopes for linguistic predictors and by-stimulus random slopes for nonlinguistic predictors.

For the wellformedness rating task, the fixed effects considered were phonotactic score as a test predictor and neighborhood occupancy rate as a control predictor. High phonotactic score indicates that the stimulus contains local sound sequences found in many Māori words, and high neighborhood occupancy rate indicates that it globally resembles many Māori words. The initial model considered the interaction between the predictors. After using a stepwise model fitting procedure (see Technical Supplement for details S1 File), results of the final model in Table 5 show that ratings significantly positively correlate with the phonotactic score, indicating that participants have phonotactic intuitions consistent with previous studies. There was no effect of neighborhood occupancy rate, suggesting that stimuli with denser phonological neighborhood do not receive higher wellformedness rating.

**Table 5:** Model results for Experiment 3 (wellformedness judgment ratings).

	Parameter	Estimate	Std. Error	*z*	*p*
Effects	Phonotactic score	5.560	0.925	6.012	<0.001
	Neighborhood occupancy rate	–2.213	2.678	–0.826	0.409
Thresholds	1|2	–2.684	0.180		
	2|3	–1.138	0.175		
	3|4	0.014	0.174		
	4|5	1.776	0.177		

**Fig 4:**
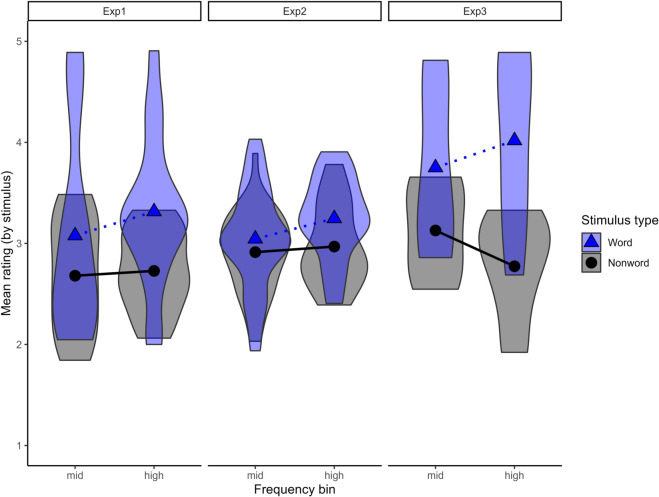
NMS’s mean wordhood confidence ratings for each stimulus type across experiments in the raw experimental results.

For the identification task, stimuli were clustered into three categories based on our experiments: real words that were defined more accurately in Exp1 (*n*  =  32 pairs) and were therefore not included in Exp2, words that were used in Exp2 (*n*  =  48 pairs), and words that appeared only in Exp3 (*n*  =  11 pairs). By comparing these categories, we sought to examine whether real words used in each experiment showed signs of being present in the proto-lexicon of the participants. The fixed effects considered were phonotactic score, word type (reference: nonwords), frequency category (reference: mid), experiment (reference: Exp1) as test predictors, and number of phonemes (continuous) as a control predictor. We analysed data beginning from an initial model that included all predictors as well as two three-way interactions. One is a three-way interaction between phonotactic score, word type and frequency category, as in the previous analysis [17,52]. The other is a three-way interaction between experiment, type and phonotactic based on results from Experiment 2. Results of the final model are in Table 6 and Fig 4 shows the relationship between mean wordhood confidence rating and stimulus-type (word and nonword) across all stimuli in the raw experimental results, for each of the different categories.

**Table 6:** Model results for Experiment 3 (wordhood confidence ratings).

	Parameter	Estimate	Std. Error	*z*	*p*
Effects	Phonotactic score	6.821	1.542	4.423	<0.001
	Type = word	1.087	0.266	4.080	<0.001
	Exp = Exp2	0.043	0.257	0.168	0.867
	Exp = Exp3	–0.004	0.380	–0.010	0.992
	Frequency category = high	0.190	0.168	1.127	0.260
	Number of phonemes	0.248	0.119	2.089	0.037
	Type = word × Exp = Exp2	–0.784	0.337	–2.329	0.020
	Type = word × Exp = Exp3	1.220	0.519	2.352	0.019
	Phonotactic score × Frequency category = high	–3.500	1.828	–1.915	0.056
Thresholds	1|2	–2.137	0.249		
	2|3	–0.345	0.248		
	3|4	1.277	0.248		
	4|5	2.816	0.249		

Across three categories, real words received higher ratings than nonwords. There is a significant effect of phonotactic score, which marginally interacts with word frequency. These results indicate that, in line with previous studies, participants gave higher ratings to stimuli with higher phonotactic scores and this effect is stronger in mid-frequency stimuli than in high-frequency stimuli. An EMM test confirms that the effect of phonotactic score is significant in each frequency category, mid: β= 6.821, z  =  4.423, *p* <.0001, high: β= 3.321, z  =  2.095, *p* = .036. The positive effect of the number of phonemes is also detected, indicating that participants are sensitive to length differences among stimuli, and longer words are more word-like. The results also show that participants gave significantly higher ratings to words than nonwords, which interacts with experiment. An EMM test confirms that the effect of word type is significant for stimulus in Experiment 1 and 3, but not significant for stimuli used in Experiment 2 (experiment  =  Exp1: word - nonword, β= 1.087, z  =  4.080, *p* <.001, experiment  =  Exp2, word - nonword, β= 0.303, z  =  1.385, *p* = 0.166, experiment  =  Exp3, word - nonword, β= 2.307, z  =  5.109, *p* <.001). These results show that while participants have proto-lexical knowledge of Māori, they cannot distinguish words from phonotactically matched nonwords in stimuli used in Experiment 2.

We also explored whether any differences between participants or between words in this experiment could predict differences in a potential lexical effect in Experiment 2, but found no evidence to support this (see Technical Supplement 4.5.6 for details S1 File). This is likely due to the fact that the variability across words and participants is not particularly high, nor is the total number of words investigated in Experiment 2.

### Summary of Experiment 3

It is clear that words that were used in Experiment 1 (the forced-choice definition task) but not in Experiment 2 (the word learning task) were adequately distinguished from phonotactically matched nonwords. Since these are the words for which participants were generally more able to discriminate between correct and incorrect definitions, it is possible that some participants had explicit knowledge of these words and others had at least a vague sense of their meaning, which likely helped them to be recognized as real words. By contrast, the words from Experiment 1 that were also used in Experiment 2 were not well distinguished from phonotactically matched nonwords. By design, these are the words that participants were less able to associate with a correct definition. Therefore, participants have little to no explicit knowledge of these words and only a limited sense of their meaning, and the results of Experiment 3 suggest that they may not have even had an implicit awareness that they are real words. Furthermore, these words were chosen from a narrow range of phonotactic scores by design, so participants could not even refer to phonotactic wellformedness as a proxy for wordhood. As a result, these specific words may not be present in the proto-lexicon for a number of NMS participants, in which case they would not be expected to facilitate the learning of meaning. Together, the findings suggest that it is plausible that NMS were not able to learn meanings of real words more accurately than nonwords in Experiment 2 because they do not have implicit form-based (i.e., proto-lexical) knowledge of those words. That is, there is no distinction between word types for those stimuli in Experiment 2 by NMS.

Previous studies have shown that NMS have implicit proto-lexical knowledge of Māori words but explicit semantic knowledge is minimal. However, our combined results suggest that their proto-lexical knowledge might be associated with vague semantic knowledge, which is different from explicit semantic knowledge. We discuss this idea in the next section.

## Discussion

We examined how a Māori proto-lexicon supports adult individuals in learning word meanings across three experiments. First, we used a forced-choice definition task to identify words for which participants had unclear semantic knowledge, to be tested further. According to our descriptive statistics, participants’ ability to define Māori words varied depending on the task format. Participants generally performed better on the forced-choice definition task—which likely engages both implicit and explicit knowledge—than on the free-response task, which relies solely on explicit recall. However, this difference may also reflect task demands: the free-response task applied strict scoring criteria that may have penalized partial semantic knowledge, while the forced-choice task’s accuracy could have been aided by the similarity of foil definitions, allowing participants with partial knowledge to select the correct option. Therefore, failure to produce an accurate definition in a free-response task does not necessarily indicate an absence of semantic knowledge within the Māori proto-lexicon.

Next, we examined whether a proto-lexicon facilitates learning meanings by comparing NMS and non-New Zealanders. NMS were significantly better at learning Māori-like word-forms, likely due to their strong phonotactic knowledge. Although all stimuli were phonotactically controlled, the word shapes were generally familiar to NMS but not to non-New Zealanders. This effect likely reflects their implicit sensitivity to the phonotactic probabilities of Māori word-forms. While other group differences could contribute, education level and initial task accuracy were similar, suggesting that NMS’s advantage stems primarily from their familiarity with these phonotactic patterns.

In terms of the predicted lexical effect, contrary to our prediction, NMS did not learn real words better than matched nonwords; their pattern mirrored that of non-New Zealanders. While previous work shows that the proto-lexicon acquired in adult NMS aids Māori word learning in educational settings [21], our results did not replicate this. We suggested three possible reasons: (1) the proto-lexicon may not facilitate learning in controlled experiments; (2) task design may have obscured the effect–for example, participants were not explicitly told the stimuli were Māori; or (3) the words used in the learning task were not represented in participants’ proto-lexicons. The second possibility was not directly tested in the current study, but could be examined in future work by manipulating how explicitly participants are cued to treat the input as Māori.

To investigate the third possibility, we conducted the word identification task (Experiment 3) using the same participants. We found that while words that were used in Experiment 2 (i.e., the word learning task) were not distinguished from phonotactically matched nonwords, words that were defined more accurately in Experiment 1 were more confidently distinguished from matched nonwords. There is thus no evidence that our participants had proto-lexical knowledge of the words that were used in Experiment 2. Additionally, the experiment confirmed that participants have robust phonotactic knowledge, consistent with previous studies [16,17,21,52], supporting the view that this phonotactic knowledge underlies their overall advantage in the learning task.

We can not rule out the possibility that if we gave participants instructions leading them to believe that they would be seeing Māori words, then this would more easily activate forms from their proto-lexicon, and a lexical effect could have emerged. However, given the results of Experiment 3, the most likely explanation for the lack of a lexical effect in our word learning experiment is that we inadvertently chose items that are not robustly represented in the proto-lexicon. In Mattingley et al. [21], participants attached real definitions to words that they had been exposed to. If we specifically selected items where we have good evidence of a proto-lexical form, it is possible that our results would be different. However, this becomes methodologically complex, because one potential interpretation of our result is that words in the proto-lexicon have some implicit semantic information associated with them, and by selecting for words with no semantics, we have inadvertently selected words that are not in the proto-lexicon. If this is the case, our design would be problematic for testing whether these words give an advantage for learning meaning, as participants may struggle to associate our fake definitions with words for which they have some competing semantic knowledge.

In general terms, building a proto-lexicon is a process of word learning before attaching meanings to word-forms [8]. The NZ Māori proto-lexicon studies cited in the background section propose the idea that while NMS have implicit form-based (proto-lexical) knowledge of many Māori words, they have explicit semantic (lexical) knowledge of very few words [19]. This rather simplified picture suggests words fall into one of three categories: not known, proto-lexical (without any meaning), or fully known.

However, based on our results from three experiments, it seems the Māori proto-lexicon might be more gradient, and consist of multiple knowledge phases. Some words in the proto-lexicon actually appear to be at the third stage of word knowledge development [55], in which individuals have vague knowledge of the word’s meaning—what Dale referred to a “twilight zone” [55] (p. 898). On the other hand, some reasonably frequent words seem not to reliably be in the proto-lexicon at all, therefore placing somewhere between the first (i.e., having never seen the word before) and second stages (knowing there is such a word but not knowing what it means). Previous work has argued that NMS possess a proto-lexicon of more than a thousand Māori words or word-parts [16]. It appears that some, or possibly even all, of these words may actually have some latent attached semantic knowledge.

This interpretation aligns with emerging evidence that proto-lexical representations, although primarily form-based, can nonetheless engage in subtle and graded semantic interactions. For example, Hendrix and Sun [54] demonstrated that participants’ responses to nonwords in lexical decision tasks were influenced by the semantic properties of their closest real-word neighbors. This suggests that sublexical or distributional features can activate partial semantic associations, even without explicit word knowledge. Such findings challenge a strict dichotomy between form and meaning, supporting the idea that proto-lexical knowledge exists along a continuum from purely phonological representations to those with partial semantic content. This may also explain the result in Experiment 2, as nonwords closely resembling real words could activate proto-lexical and semantic networks to some degree, potentially obscuring clear lexical advantages during learning.

Implicit knowledge of Māori in NMS continuously expands and is reinforced with continuous exposure to Māori across the lifespan [52]. This raises questions about the exact nature of the proto-lexicon underpinning the results found in the past work on NMS. It also raises a number of questions about the role of these forms in generalization. What degree of ‘embeddedness’ is required, for example, before the word-form can start to facilitate explicit acquisition of meaning? How can we design tasks to robustly distinguish these different degrees and types of knowledge? Do all stored word-forms feed phonotactic generalizations to the same degree, or do more robust and rich word-forms play a larger role? Future studies should explore such effects in learning the meaning of words.

## Conclusion

This study focuses on a ‘proto-lexicon’—a set of stored forms— and how the proto-lexicon assists learning meanings of words in an experimental setting. The results of three experiments indicate that there are different levels of semantic knowledge for different words even when we consider only words that cannot confidently be said to be in a full lexicon. These findings suggest that the claim of previous studies [16,17] that the proto-lexicon is ‘without semantics’ may be over-simplified.

## Supporting information

S1 File Technical Supplement: Exploring the role of meaning in non-Māori speakers’ ‘proto-lexicon’. (HTML)
