# Supplementary material for: Exploring the role of meaning in non-Māori speakers’ ‘proto-lexicon’
Source: PLoS One. 2026 Jan 29;21(1):e0339325. doi: 10.1371/journal.pone.0339325 (PMC12854419; doi:10.1371/journal.pone.0339325)
Supplement: S1 File — (HTML) [file pone.0339325.s001.html]

Exploring the role of meaning in non-Māori speakers’ ‘proto-lexicon’


Code 

- Show All Code
- Hide All Code

# Exploring the role of meaning in non-Māori speakers’ ‘proto-lexicon’

### Supplementary Materials

#### 

#### December 19, 2025

# 1 Introduction

This R Markdown file contains supplementary information and all the
code used for data exclusion, analysis, and plotting. This document is
split into six sections. Section 2, Section 3, and Section
4 describe the data preparation process, including the dataset
structure and the factors associated with the stimuli for each
experiment. They also describe the experimental methodology for each
task, provide the code for the data filtering process, and detail
participant demographics for the analysis of the data. Section 5 describes the post-experiment
questionnaires used in the experiments. Finally, Section 6 lists the stimuli used in
Experiment 1, Experiment 2, and Experiment 3.

```
# Setup -----
library(pacman)
p_load(knitr, tidyverse, ordinal, effects, MASS, kableExtra, egg, lme4, sjPlot, ggeffects, ggpubr, ggrepel,broom, parameters, MuMIn, emmeans, car)
#library(knitr)
#library(tidyverse)
#library(ordinal)
#library(effects)
#library(MASS)
#library(kableExtra)
#ibrary(egg)
opts_chunk$set(echo=TRUE, message=FALSE, warning=FALSE, fig.show='hold', results='hold')

# Local functions ----
# A function to calculate confidence intervals
CI <- function (x, ci = 0.95) 
{
    a <- mean(x)
    s <- sd(x)
    n <- length(x)
    error <- qt(ci + (1 - ci)/2, df = n - 1) * s/sqrt(n)
    return(c(upper = a + error, mean = a, lower = a - error))
}


# A function to center variables
c. <- function (x) scale(x, scale = FALSE)

# Custom function to format ordinal regression results in a nice table
clm_table <- function(mod, digits = 3, sep = " = ", interaction_sep = " : ", ...) {

  table <- summary(mod)$coefficients %>%
    data.frame() %>%
    rlang::set_names(c("beta", "se", "z", "p")) %>%
    rownames_to_column("parameter") %>%
    mutate(
      is_threshold = parameter %in% names(mod$alpha),
      parameter = ifelse(
        is_threshold,
        gsub("\\|", "\u00A6", parameter),
        parameter
      )
    )
  
  factor_names <- names(attr(mod$terms, "dataClasses"))[
    attr(mod$terms, "dataClasses") %in% c("factor", "logical", "character")
  ]
  table$parameter <- unname(sapply(table$parameter, function(param) {
    parts <- strsplit(param, ":", fixed = TRUE)[[1]]
    parts <- sapply(parts, function(p) {
      # Centered variables
      p <- str_replace_all(p, "(?:c\\.\\()([^\\)]+)\\)", "\\1 (centered)")
      # Factor-level replacement
      for (fn in factor_names) {
        pattern <- paste0("^", fn, "(\\w+)$")
        p <- str_replace(p, pattern, paste0(fn, sep, "\\1"))
      }
      return(p)
    })
    paste(parts, collapse = interaction_sep)
  }))
  
  table$significance <- case_when(
    !table$is_threshold & table$p < 0.001 ~ "***",
    !table$is_threshold & table$p < 0.01  ~ "**",
    !table$is_threshold & table$p < 0.05  ~ "*",
    TRUE ~ ""
  )
  
  table$z[table$is_threshold] <- NA
  table$p[table$is_threshold] <- NA
  table$significance[table$is_threshold] <- NA

  table$p <- ifelse(table$is_threshold, NA,
                    ifelse(table$p < 0.001, "<0.001",
                           format(round(table$p, digits), nsmall = digits)))
  
  effects <- table[!table$is_threshold, ]
  thresholds <- table[table$is_threshold, ]

  if (nrow(effects) > 0) effects$type <- c("Effects", rep("", nrow(effects) - 1))
  if (nrow(thresholds) > 0) thresholds$type <- c("Thresholds", rep("", nrow(thresholds) - 1))
  
  table <- rbind(effects, thresholds) %>%
    dplyr::select(type, parameter, beta, se, z, p, significance)
  
  kable(table, digits = digits, escape = TRUE,
        col.names = c("", "Parameter", "Estimate", "Std. Error", "z", "p", ""),
        align = "llrrrrl",
        row.names = FALSE,
        ...) %>%
    column_spec(1, bold = TRUE) %>%
    kable_styling()
}

# Prevent NA from printing in kable
options(knitr.kable.NA = '')

# Custom function to compute summary data for an ordinal regression model, to be plotted. The "type" argument specifies whether the data are for a latent variable plot, a distributional plot, or a mean rating plot. 
clm_plotdat = function(mod, focal.predictors, type="latent", response.coding=NULL, ...) {
  # For latent variable plot, draw straight from Effect
  if (type=="latent") {
    latentdat <-
      Effect(focal.predictors, mod, latent=TRUE, ...) %>%
      as.data.frame() %>%
      dplyr::select(-se) %>%
      rename("pred"=fit, "lci"=lower, "uci"=upper)
    return(latentdat)
  }
  
  # Get distributional data (used for both of other plots)
  distdat <-
    Effect(focal.predictors, mod, ...) %>%
    as_tibble() %>%
    dplyr::select(all_of(focal.predictors), matches("^(?:(?:L|U)\\.)?prob")) %>%
    pivot_longer(
      cols = -all_of(focal.predictors),
      names_to = c(".value", "response"),
      names_pattern = "^((?:(?:L|U)\\.)?prob)\\.(.+)$"      
    ) %>%
    rename("pred"=prob, "lci"=L.prob, "uci"=U.prob) %>%
    mutate(
      response = rep_len(mod$y.levels, n())
    )
  if (type=="dist") return(distdat)
  
  if (type=="mean") {
    # Get threshold values
    thresholds = c(-Inf, as.numeric(mod$alpha), Inf)
    
    # Get response coding
    if (is.null(response.coding)) {
      response.coding = 1:(length(mod$y.levels))
      names(response.coding) = mod$y.levels
    }
    
    # Build latent variable data based on distributional data
    # Note: this is a HACK since drawing latent variable data from Effect doesn't account for threshold variability. The confidence intervals assume fixed thresholds, which obscures patterns in variation of the thresholds; CIs are thus not correct (but are better than what would be obtained from direct transformation of the latent variable from Effect)
    
    # Get distribution-based latent variable data, assuming fixed thresholds
    latentdat <- distdat %>%
      group_by_at(focal.predictors) %>%
      summarise(
        pred = qlogis(1-pred[1]) + thresholds[2],
        lci = qlogis(1-uci[1]) + thresholds[2],
        uci = qlogis(uci[length(uci)]) + thresholds[length(thresholds)-1]
      ) %>%
      as.data.frame()    
    
    # Get probabilities for each response option
    meandat <- latentdat
    for (i in 1:length(mod$y.levels)) {
      meandat[, paste0("(", mod$y.levels[i], ").prob")] = plogis(latentdat[, "pred"] - thresholds[i]) - plogis(latentdat[, "pred"] - thresholds[i+1])
      meandat[, paste0("(", mod$y.levels[i], ").lci")] = plogis(latentdat[, "lci"] - thresholds[i]) - plogis(latentdat[, "lci"] - thresholds[i+1])
      meandat[, paste0("(", mod$y.levels[i], ").uci")] = plogis(latentdat[, "uci"] - thresholds[i]) - plogis(latentdat[, "uci"] - thresholds[i+1])
    }
    meandat <- meandat %>%
      dplyr::select(-pred, -uci, -lci) %>%
      pivot_longer(
        cols = -all_of(focal.predictors),
        names_to = c("response", ".value"),
        names_pattern = "^\\((.+)\\)\\.([^.]+)$"
      ) %>%
      left_join(
        data.frame(response=names(response.coding), code=response.coding, stringsAsFactors=FALSE),
        by = "response"
      ) %>%
      group_by_at(focal.predictors) %>%
      summarise(
        pred = sum(prob * code),
        lci = sum(lci * code),
        uci = sum(uci * code)
      ) %>%
      ungroup()
    return(meandat)
  }
}

# Custom function to get the information about thresholds, in a nice format for plotting
clm_thresholds = function(mod) {
  thresholds <- summary(mod)$coefficients %>%
  as.data.frame() %>%
  rownames_to_column() %>%
  dplyr::select(1:3) %>%
  set_names(c("label", "value", "se")) %>%
  filter(label %in% names(mod$alpha)) %>%
  mutate(
    lci = value - 1.96*se,
    uci = value + 1.96*se
  ) %>%
  dplyr::select(-se)
  return(thresholds)
}

# A function to add phonotactic scores from a file to the dataset
add_scores = function(data, scoreFile, scoreName) {
  scored_data <- data %>%
    left_join(
      read_csv(scoreFile, col_types=cols_only(item=col_character(),
                                              logprob=col_double())
               )%>%
        transmute(
          item = item,
          !!scoreName := logprob / (nchar(item) + 1)
        )
      , by="item"
    )
  return(scored_data)
}

# A function to run shell scripts on Windows or UNIX-based OS
run_shell = function(cmd) {
  if (Sys.info()[['sysname']] == "Windows") {
    return(shell(cmd, flag="", ignore.stdout=TRUE, ignore.stderr=TRUE))
  } else {
    return(system(cmd, ignore.stdout=TRUE, ignore.stderr=TRUE))
  }
}

# A function to highlight the minimum value in a column, for presentation in a kable
# Highlighting is accomplished by coloring red
highlight_min = function(col, digits=1) {
  rounded <- round(col, digits)
  i <- which(rounded==min(rounded))
  highlighted <- cell_spec(format(rounded, nsmall=digits), align="r")
  highlighted[i] <- cell_spec(format(rounded[i], nsmall=digits), align="r", color="red")
  return(highlighted)
}

# A function to display a table of numbers
display_table = function(dat, caption, digits=3, highlight=c()) {
  table <- dat %>%
    mutate_at(highlight, ~ highlight_min(., digits=digits)) %>%
    kable(caption=caption, digits=digits, escape=F) %>%
    kable_styling()
  return(table)
}

# Custom function to format logistic regression results in a nice table
logistic_table <- function(mod, digits = 3, interaction_sep = ":", ...) {
  coefs <- summary(mod)$coefficients
  coefs <- data.frame(coefs)
  colnames(coefs) <- c("beta", "se", "z", "p")
  coefs$parameter <- rownames(coefs)
  rownames(coefs) <- NULL
  
  # Fix indent
  coefs$parameter <- str_trim(coefs$parameter)
  coefs$parameter <- str_replace_all(
    coefs$parameter,
    "c\\.\\(([^)]+)\\)",
    "\\1 (centered)"
  )

  coefs$parameter <- sapply(coefs$parameter, function(param) {
    if (param == "(Intercept)") return(param)
    if (str_detect(param, "^type")) {
      level <- str_sub(param, start = 5)
      return(paste("type", level))
    }
    
    if (str_detect(param, "\\.")) {
      dots <- str_locate_all(param, fixed("."))[[1]][,1]
      last_dot <- tail(dots, 1)
      var <- substr(param, 1, last_dot)
      level <- substr(param, last_dot + 1, nchar(param))
      return(paste(var, level))
    }
    
    param
  })
  
  coefs$parameter <- str_replace_all(
    coefs$parameter,
    fixed(":"),
    paste0(" ", interaction_sep, " ")
  )
  
  coefs$significance <- ifelse(
    coefs$p < 0.001, "***",
    ifelse(coefs$p < 0.01, "**",
           ifelse(coefs$p < 0.05, "*",
                  ifelse(coefs$p < 0.1, ".", "")))
  )
  coefs$p <- ifelse(
    coefs$p < 0.001,
    "<0.001",
    format(round(coefs$p, digits), nsmall = digits)
  )
  coefs <- coefs[, c("parameter", "beta", "se", "z", "p", "significance")]   # Reorder columns
  kable(
    coefs,
    digits = digits,
    escape = FALSE,
    col.names = c("Parameter", "Estimate", "Std. Error", "z", "p", ""),
    align = "lrrrrl",
    ...
  ) %>%
    kable_styling()
}

## Variance Inflation Factor (VIF)
#Variance inflation factor (VIF) can measure how much the variance of a regression coefficient is inflated due to multicollinearity in the model. The code used for the VIF test was below. A VIF of 5 was used as a cutoff point.
vif.mer <- function (fit) {
  ## adapted from rms::vif
  
  v <- vcov(fit)
  nam <- names(fixef(fit))
  
  ## exclude intercepts
  ns <- sum(1 * (nam == "Intercept" | nam == "(Intercept)"))
  if (ns > 0) {
    v <- v[-(1:ns), -(1:ns), drop = FALSE]
    nam <- nam[-(1:ns)]
  }
  
  d <- diag(v)^0.5
  v <- diag(solve(v/(d %o% d)))
  names(v) <- nam
  v
}

# A table for emmeans results
em_table = function(mod, ...){
table <- summary(mod) %>% mutate(across(where(is.numeric), round, 3)) %>% mutate(
    significance = case_when(
      p.value < 0.001 ~ "***",
      p.value < 0.01 ~ "**",
      p.value < 0.05 ~ "\\*",
      p.value < 0.1 ~ ".",
      TRUE ~ ""),
    p.value = ifelse(p.value<0.001, "<0.001", format(round(p.value, 3), nsmall=3))) %>% 
    dplyr::select(-df)%>% 
 # kable(digits = 3,col.names=c("Parameter","Estimate","Std. Error","lower CL","upper CL","$z$", "$p$", ""),align="lrrrrrrl") %>% kable_styling() 
#return(table)
  return()
}

# tidy emm table output when p.value present
emm_tidy = function(table, ...){
  
  temp_table = summary(table) %>% mutate(
    significance = case_when(
      p.value < 0.001 ~ "***",
      p.value < 0.01 ~ "**",
      p.value < 0.05 ~ "\\*",
      p.value < 0.1 ~ ".",
      TRUE ~ ""),
    p.value = ifelse(p.value<0.001, "<0.001", format(round(p.value, 3), nsmall=3))) %>% 
    dplyr::select(-df) %>% return()
  
}
```

# 2 Experiment 1: Word definition Experiment

Experiment 1 consisted of a word definition task and a word splitting
task. Participants completed a post-experiment questionnaire which
included 27 demographic questions. While participants completed the word
splitting task, this is not reported on here. The primary purpose of the
word definition task was to select words that are not widely known by
NMS for Experiment 2. The experimental session lasted less than 30
minutes in total. The details of each task are given below.

## 2.1 Data Preparation and Method

### 2.1.1 Material

The set of stimulus materials used in this experiment was a subset of
the items of Panther et al. (2023)

There were 165 real Māori words from the high and mid frequency
categories in the sample of Panther et al. (2023). Among them, we
removed 14 words: six words that showed high accuracy in Oh et
al. (2023), nine transliterated words, four words whose definitions
overlap with other words. As a result, there were 146 words in
total.

- We presented these same stimuli to each participant in a different
  random order.

```
df <- read.table("exp1/WordHighMid146.tsv", sep = "\t", header = TRUE, quote = "")

summary_table_exp1 <- df %>%
   summarise(
    Mean_Length = mean(n.phonemes, na.rm = TRUE),
    SD_Length = sd(n.phonemes, na.rm = TRUE),
    Mean_Phonotactic_Score = mean(score.shortv, na.rm = TRUE),
    SD_Phonotactic_Score = sd(score.shortv, na.rm = TRUE),
    Mean_Neighborhood_Density = mean(norm.neighbors, na.rm = TRUE),
    SD_Neighborhood_Density = sd(norm.neighbors, na.rm = TRUE)
  )  %>%
  mutate(across(where(is.numeric), ~ round(.x, 2)))

#print(summary_table_exp1)

remove(df, summary_table_exp1)
```

### 2.1.2 Task Procedure

- For each trial, participants saw a stimulus word and a definition in
  the middle of the screen. The definition was either a real definition or
  an incorrect definition. Participants were asked to answer whether the
  presented word-definition pair is ‘good’ or ‘not good’. After the
  participant clicked one of the options and the ‘Next’ button, the next
  stimulus was presented.

### 2.1.3 Data Filtering

68 participants completed the entire experiment, of whom 13
participants were removed. We filtered out those who met any of the
following criteria based on answers from the post-experiment
questionnaire (see Section 5
Questionnaires for all questions).

- 6 participants with suspicious patterns of the participation into
  the experiment. (removed beforehand)
- 2 participants were removed for indicating that they could speak or
  understand Māori at least “fairly well”
- 3 participants were removed for indicating that they had lived
  outside NZ for longer than a year since they were 7
- 2 participants were removed for indicating that they learned their
  first language outside New Zealand and have lived in their current
  location for a short period of time.

To evaluate whether participants relied on external aids, we examined
their total task duration and median reaction times. If a participant’s
median reaction time exceeded two standard deviations above the group
median, we also analysed their accuracy. Two participants exceeded this
threshold, and their accuracy was near chance level (51% and 56%),
suggesting they were not using external aids.

Thus, the data consisted of 55 participants for Experiment 1.

```
defShort <- read.delim("exp1/defshort.tsv", sep ="\t", header = TRUE, encoding="UTF-8")
nb_res<- defShort %>% dplyr::select (PID, stimulus) %>% distinct_all() %>% group_by(PID)%>%  dplyr::summarise (responses = n()) #62 participants completed 146 

remove(nb_res)
```

```
# Word Definition Task
# data merge with stimulus info

word_defs <- read.table("exp1/WordHighMid146.tsv", sep = "\t", header = TRUE, quote = "")

defShort <- defShort %>%
  rowwise() %>%
  mutate(
    correct_def = info == word_defs$definition[word_defs$word == stimulus],
                       freq.cat = word_defs$freq.category[word_defs$word== stimulus],
                        p.scr = word_defs$score.shortv[word_defs$word == stimulus],
                        definition = word_defs$definition[word_defs$word == stimulus],
  )

remove(word_defs)

### nb_response items
## nb response for each item
## Each word should appear 55 times. 
nb_res<- defShort %>% dplyr::select (PID, word) %>% distinct_all() %>% group_by(word) %>%  dplyr::summarise (responses = n())

# nb response for two options
# Even number of options; 73 responses for each option

nb_opt<- defShort %>% dplyr::select (PID,stimulus, correct_def)  %>% group_by(PID, correct_def )%>%  dplyr::summarise (responses = n())

remove(nb_res, nb_opt)

### participant responses
## Check participant answers
## Some clicking the same option all the time during the session
prop_answers <- table(defShort$PID, defShort$enteredResponse) %>%
  data.frame() %>%
  group_by(Var1) %>%
  mutate(Prop = Freq/sum(Freq))

### accuracy creating a correct column
#length(unique(defShort$stimulus)) #146
#length(unique(defShort$PID)) #62

## accuracy, assign 1 for correct responses, 0 for incorrect reposes
mutate(defShort,
  correct = case_when(
    correct_def == "TRUE" & enteredResponse == "good" ~ 1,
    correct_def == "FALSE" & enteredResponse == "not good" ~ 1,
   TRUE ~ 0))->defShort
```

```
# 68 participants
# 6 participants with suspicious patterns of the participation into the experiment. (removed beforehand)
#length(unique(defShort$PID)) #62

# speaking and understanding of Māori (Q1 and Q2)
rmParticipantsMaoriProf <- unique(defShort[defShort$speakMaori >= 3 | defShort$compMaori >= 3,]$PID) # 2 participants
defShort <-defShort[!defShort$PID %in% rmParticipantsMaoriProf,]
remove(rmParticipantsMaoriProf)

#length(unique(defShort$PID)) #60

# lived outside NZ (Q10)
rmParticipantOutsideNZ <- unique(defShort[defShort$outsideNz=="Yes",]$PID) # 3
defShort  <- defShort [!defShort$PID %in% rmParticipantOutsideNZ,]
remove(rmParticipantOutsideNZ) #57

## non-English first language (Q19)
rmFirstLangMaori <- unique(defShort$PID[defShort $firstLang != "English"]) #0
defShort  <-defShort [!defShort$PID %in% rmFirstLangMaori,]
remove(rmFirstLangMaori) #57

# Remove participants who did not learn their English in NZ and have been living in their current location in NZ for less than ten years (duration == "short")
summaryPID <- unique(defShort[,c("PID","firstLangCountry","regionCurrentLive","duration")])
EngNotInNZ <- summaryPID[!summaryPID$firstLangCountry=="NZ",]# other 4
rmParticipantFirstLang <- unique(EngNotInNZ[EngNotInNZ$duration=="short",]$PID) # 3
defShort  <- defShort [!defShort$PID %in% rmParticipantFirstLang,]
remove(summaryPID, EngNotInNZ, rmParticipantFirstLang) #55

rmParticipantPoly <- unique(defShort[defShort$anyPolynesian=="Yes",]$PID) #0
remove(rmParticipantPoly) #55

# check duration
getTime <- function(x){
  firstTime <- min(x)
  lastTime <- max(x)
  return(difftime(lastTime, firstTime, units="mins"))
}

timings <- aggregate(defShort$dateTime, by=list(defShort$PID),  FUN=getTime)
names(timings)<-c("PID", "Duration")
timings <- timings[order(timings$Duration),]
timings$Duration<-as.numeric(timings$Duration)

# check reaction time
# fast participants
PIDmedians <- defShort %>% group_by(PID) %>% mutate(medianTime = median(reactionTime)) %>% subset(select=c(PID, medianTime)) %>% unique()
medianSd <- sd(PIDmedians$medianTime)
twoSdCutoff <- (mean(PIDmedians$medianTime) - medianSd) - medianSd
fastIDs <- PIDmedians$PID[PIDmedians$medianTime < twoSdCutoff] # 0 IDs

remove(twoSdCutoff, fastIDs)

twoSdCutoff <- (mean(PIDmedians$medianTime) + medianSd) + medianSd
slowIDs <- PIDmedians$PID[PIDmedians$medianTime > twoSdCutoff]

slowPat <- ggplot(defShort %>% filter(PID %in% c("9", "46","31")),  aes(x = trialNumber, y = reactionTime)) +
  geom_point(alpha = 0.6, size = 1, color = "steelblue") +
  facet_wrap(~ PID, scales = "free_x") +
  labs(
    title = "Reaction Times per Trial by Participant",
    x = "Trial Number",
    y = "Reaction Time (s)"
  ) +
  theme_minimal()

target_PIDs <- c("9","46", "31")
target_accuracy <-defShort %>%
  filter(PID %in% target_PIDs) %>%
  group_by(PID) %>%
  summarise(
    Accuracy = mean(correct, na.rm = TRUE),
    Trials = n()
  ) ## 9:0.51, 31:0.56, 46:0.56

#length(unique(defShort$PID)) # 55

remove(getTime, timings, PIDmedians, twoSdCutoff, slowIDs, slowPat, target_PIDs, target_accuracy, prop_answers)
```

After excluding these participants, 55 adult native speakers of New
Zealand English remained (45 female). All participants were aged between
18 and 60 years and did not have sufficient proficiency in Māori to hold
a basic conversation in Māori, in line with the inclusion criteria.

Participants rated their Māori speaking and understanding abilities
using 6 options ranging from “Not at all” to “Very well.” These ratings
were coded from 0 to 5 and combined to create a Māori proficiency score
ranging from 0 to 10. Most participants reported being able to speak or
understand “no more than a few words or phrases” (scoring 2 or
below).

Regarding other language experience, 87.3% were monolingual
(responding “none”), while 12.7% reported speaking one additional
language, such as Japanese, French, or Spanish.

```
# 55 participants
# Code for Figure S1: Plot participant demographics

data <- defShort %>% dplyr::rename("basic_knowledge" = "maoriList","exposure" = "maoriExpo", "proficiency" = "maoriProf")

# Population and Set Bar Graph
# Age 
fig.age <-data %>% ungroup() %>% dplyr::select(PID,age) %>% distinct() %>% 
  ggplot(aes(x=factor(age), fill=factor(age)))+
  geom_bar(aes(),size=.8,position="dodge",show.legend=F) + 
  geom_text(stat='count', aes(label= after_stat(count)), vjust=1) +
  scale_x_discrete("Age group", 
                   labels=c("1"="18-29","2"="30-39","3"="40-49","4"= "50-59","5" = "+60"))+
  ggtitle("")+ylab('No. of People')
#fig.age 

# Gender
# change levels
 data<- data %>%mutate(
   gender1 = fct_recode(as.factor(gender),
                     male = "man",
                     female = "woman",
                      NB = "NB",
                      NS = "NS"))
 
fig.gender<-data %>% ungroup() %>% dplyr::select(PID,gender1) %>%  mutate(gender1 = fct_relevel(gender1, c("female", "male", "NB", "NS"))) %>% distinct()  %>%
 ggplot(aes(x=factor(gender1), fill=factor(gender1)))+
  geom_bar(aes(),size=.8,position="dodge",show.legend=F) + 
  geom_text(stat='count', aes(label = after_stat(count)), vjust=0.5)+
  ggtitle("")+
  xlab('Gender')+
  ylab('No. of People')
#fig.gender

# Highest Education 
# change levels
data$education<- factor(data$education, levels=c("high school","certificate or diploma","undergraduate","graduate"))

fig.edu<-data %>% ungroup() %>% dplyr::select(PID,education) %>% distinct()  %>%
 ggplot(aes(x=factor(education), fill=factor(education)))+
  geom_bar(aes(),size=.8,position="dodge",show.legend=F) + 
  geom_text(stat='count', aes(label = after_stat(count)), vjust=0.7)+
  #theme(axis.text.x = element_text(angle =45, hjust = 1))+
  scale_x_discrete(guide = guide_axis(n.dodge=3))+
  ggtitle("")+
  xlab('Highest education')+
  ylab('No. of People')

#fig.edu

#Region Most Island
fig.region<-data %>% ungroup() %>% dplyr::select(PID,island) %>% distinct() %>%
  ggplot(aes(x=factor(island), fill=factor(island)))+
geom_bar(aes(),size=.8,position="dodge",show.legend=F) + 
  geom_text(stat='count', aes(label = after_stat(count)), vjust=0.6)+
  ggtitle("")+
  xlab('Region most spent in NZ (island)')+
  ylab('No. of People')

#fig.region

# Māori exposure
fig.expo<-data %>% ungroup() %>% dplyr::select(PID,exposure) %>% distinct() %>%
  ggplot(aes(x=factor(exposure), fill=factor(exposure)))+
  geom_bar(aes(), size =.1, show.legend = F) +
  #scale_x_discrete("Exposure to Māori",labels=as.character(c("2"=2,"3"=3,"4"=4,"5"=5, "6"=6,"7"=7,"8"=8,"9"=9,"10"=10)))+
  geom_text(stat='count', aes(label= after_stat(count)), vjust=1) +
   scale_y_continuous(limits = c(0, 12), breaks = seq(0, 12, 2)) +
  #scale_fill_manual(name ="PID", values = cbPalette, guide = guide_legend(reverse = TRUE))+
  ggtitle("")+
  xlab('Māori exposure')+
  ylab('No. of People')
#fig.expo

# Māori proficiency
fig.prof<-data %>% ungroup() %>% dplyr::select(PID,proficiency) %>% distinct() %>% 
  ggplot(aes(x=factor(proficiency), fill=factor(proficiency)))+
  geom_bar(aes(),size=.8,position="dodge",show.legend=F) + 
  geom_text(stat='count', aes(label = after_stat(count)), vjust=0.6)+
   ggtitle("")+
  xlab('Māori proficiency')+
  ylab('No. of People')
#fig.prof

# Basic Knowledge of Māori
fig.knowledge <- data %>% ungroup() %>% dplyr::select(PID,basic_knowledge) %>% distinct() %>% 
  ggplot(aes(x=factor(basic_knowledge), fill=factor(basic_knowledge)))+
  geom_bar(aes(),size=.8,position="dodge",show.legend=F) + 
    geom_text(stat='count', aes(label = after_stat(count)), vjust=0.6)+
    scale_y_continuous(limits = c(0, 10), breaks = seq(0, 10, 2)) +
  ggtitle("")+
   xlab('Basic knowledge of Māori')+
  ylab('No. of People')
#fig.knowledge

# Other languages
# create a new column 'numLanguages' that counts languages
data$numLangs <- sapply(data$otherLangs, function(x) {
  if (x == "None") {
    return("None")  # Monolingual (none) as "None"
  } else if (x == "") {
    return(NA)  # Handle any empty strings, if any exist
  } else {
    return(as.character(length(strsplit(x, ",")[[1]])))  # Convert count to character for consistency
  }
})

# convert numLanguages to a factor and make sure 'None' is included
data$numLangs <- factor(data$numLangs, levels = c("None", "1"))

# check for any NA values in numLanguages
#sum(is.na(data$numLangs))

#list(unique(data$numLangs))
#table(data$numLangs)

fig.otherLangs <- data %>% ungroup() %>% dplyr::select(PID,numLangs) %>% distinct() %>%
  ggplot(aes(x=factor(numLangs), fill=factor(numLangs)))+
  geom_bar(aes(),size=.8,position="dodge",show.legend=F) + 
    geom_text(stat='count', aes(label = after_stat(count)), vjust=0.6)+
    scale_y_continuous(limits = c(0, 50)) +
  ggtitle("")+
   xlab('Other languages')+
  ylab('No. of People')
#fig.otherLangs

ggarrange(fig.age, fig.gender,fig.edu,fig.region, fig.expo,fig.prof,fig.knowledge, fig.otherLangs)
remove(fig.age, fig.gender,fig.edu, fig.region, fig.expo,fig.prof,fig.knowledge, fig.otherLangs)

remove(data)
```

Overview of participants’ demographics (n = 55)

## 2.2 Results

### 2.2.1 Summary of Raw Data

The plots below show the mean response for each stimulus.

The rate of correct responses varied among words, ranging between 33%
for *kuhu* “to enter”, *niao* “rim, edge” and 93 % for
*whenua* ‘land’ (top and middle). The participants selected
correct options with more than 50% chance for 109 words out of 146 words
(75%), which indicates that they can identify word meanings in Māori
words for a substantial subset of the words.

There is no relationship between phonotactic scores of words and
definition accuracy in the raw data (bottom).

```
## Item 
## D Prime for items
defShort <- defShort %>%
  group_by(stimulus) %>%
  mutate(hit_item = length(PID[correct_def == TRUE & enteredResponse == "good"])/length(PID[correct_def == TRUE]),
         FA_item = length(PID[correct_def == FALSE & enteredResponse == "good"])/length(PID[correct_def == FALSE]),
         item_dp = round(qnorm(hit_item) - qnorm(FA_item),3)
         )

#  defShort %>%
#  dplyr::select(stimulus, hit_item) %>%
#  distinct()

## accuracy rate per item
defShort<- defShort %>% group_by(stimulus) %>% mutate(item_accuracy_mean = round(mean(correct),3),item_accuracy_sd = round(sd(correct), 3))

item.summary_55<-defShort  %>% group_by(stimulus) %>% dplyr::select(stimulus,item_dp, item_accuracy_mean, item_accuracy_sd, definition) %>% distinct() %>% ungroup()  

#high_score_words <- table(defShort$stimulus, defShort$enteredResponse) %>%
#  data.frame() %>%
#  group_by(Var1) %>%
#  mutate(Prop = Freq/sum(Freq))

# photostatic effect
p.scr_plot_rate = ggplot(defShort, aes(x=p.scr, y=item_accuracy_mean, label =stimulus)) + 
  geom_point(size=3, alpha=0.2, shape=17, color="#4682B4") + 
  geom_text(hjust=0.2, vjust=-0.7, check_overlap=TRUE, size=4, color="black") + 
  #geom_smooth(method="lm", color = "blue", fill = "red") +
   theme_minimal() +
  scale_x_continuous(expand=expansion(mult=0.1)) +
  #ylim(-1, 1.05)+
  theme_bw()  +
  xlab("Phonotactic Score") +
  ylab("Accuracy Rate")
#p.scr_plot_rate 

## accuracy for each word
word_accuracy_data = defShort %>% 
  dplyr::select(stimulus, item_accuracy_mean) %>% 
  ungroup %>%
  distinct() %>%
  arrange(item_accuracy_mean) %>%
  mutate(id= row_number()) 

plot1 <- word_accuracy_data %>% filter(id %in% 74:146) %>%
  ggplot(aes(x = reorder(stimulus, -item_accuracy_mean), y = item_accuracy_mean, label = stimulus)) +
  geom_bar(stat = "identity",  color = "#4682B4", width = 1) +
  theme(axis.text.x = element_text(size =9, angle = 90, hjust = 1, vjust = .5)) +
  #scale_x_discrete(breaks = NULL) +
  #theme_minimal() +
  scale_y_continuous(limits = c(0,1))+
  xlab("Words") +
  ylab("Accuracy Rate")

plot2 <- word_accuracy_data  %>% filter(id %in% 1:73) %>%
  ggplot(aes(x = reorder(stimulus, -item_accuracy_mean), y = item_accuracy_mean, label = stimulus)) +
  geom_bar(stat = "identity",  color = "#4682B4", width = 1) +
  theme(axis.text.x = element_text(size =9, angle = 90, hjust = 1, vjust = .5)) +
  #scale_x_discrete(breaks = NULL) +
  #theme_minimal() +
  scale_y_continuous(limits = c(0,1))+
  xlab("Words") +
  ylab("Accuracy Rate")

#ggsave(file="Accuracy Rate per item.png", width=10, height=6, dpi=300)
ggarrange(plot1, plot2, p.scr_plot_rate,ncol=1) 
remove(prop_answers, p.scr_plot_rate, word_accuracy_data, plot1, plot2)
```

Summary of raw data

### 2.2.2 The Word List for Experiment 2

The primary purpose was to identify Māori words whose definitions NMS
cannot easily recognize, to be used as stimuli in the world learning
task in Experiment 2.

To determine the overall accuracy of definitions for each participant
and each word, we calculate a d-prime score.

`D-prime score`: d’(d-prime) indicates each participants’
sensitivity to definitions (‘good’ vs. ‘not good’) in the experiment. d’
scores are calculated based on ‘good’ responses for both true and false
definitions for each stimulus word.

- Calculated based on the difference between the scored hit rate
  (proportion of true definition given a ‘good’ response) and their
  z−scored false alarm rate (proportion of false definitions given a
  response ‘good’).
- Participants with high d-prime scores give many true definitions
  and few false definitions a response ‘good’. Participants with low
  d-prime scores showed the opposite performance.

The formula for d-prime is as follows.

\[d' = z\left(\frac{\text{# true
definitions responded `good'}}{\text{total # true
definitions}}\right) - z\left(\frac{\text{# false definitions responded
`good'}}{\text{total # false definitions}}\right)\]

We also calculated a d-prime score for each stimulus, which
ranged between −0.82 and 3.46 as shown in the figure below. Words with
higher d-prime were more accurately identified by participants as
matched or mismatched to their paired definitions.

```
hp <- defShort %>%
  filter(freq.cat == "high") %>%
  dplyr::select(word, item_dp, p.scr) %>%
  distinct() %>%
  ggplot(aes(x = p.scr, y = item_dp)) +
  geom_point(stat="identity") +
  geom_label(aes(label=word), size = 3) +
  theme_minimal() +
  #scale_x_discrete(breaks = NULL) +
  ylab("D-Prime") +
  xlab("Phonotactic score") +
  ggtitle("High frequency Words")

mp <- defShort %>%
  filter(freq.cat == "mid") %>%
  dplyr::select(word, item_dp, p.scr) %>%
  distinct() %>%
  ggplot(aes(x = p.scr, y = item_dp)) +
  geom_point(stat="identity") +
  geom_label(aes(label=word), size = 3) +
  theme_minimal() +
  #scale_x_discrete(breaks = NULL) +
  ylab("D-Prime")  +
  xlab("Phonotactic score") +
  ggtitle("Mid frequency Words")

ggarrange(hp, mp, nrow = 2)

candidate<-defShort %>%
  dplyr::select(word, item_dp, freq.cat, p.scr) %>%
  distinct() %>%
  mutate(dp_abs = abs(item_dp)) %>%
  filter(dp_abs < 0.3) %>%
  ggplot(aes(x = p.scr, y = item_dp, color = freq.cat, label = word)) +
  geom_point(size = 5) +
  geom_label() +
  theme_minimal() +
  theme(
    legend.position = "none"
  )+ ggtitle("Items with d-prime lower than 0.3")
```

The relationship between d-prime score and phonotactic score per
stimulus item.

On the basis of d-prime and phonotactic score of each word, we
selected stimuli by cutoff at 0.3 d-prime and at −0.89 phonotactic
score, resulting in 48 words for the word learning task (Experiment 2).
This means that we chose words with low d-prime scores, which are less
likely to be associated with semantic knowledge. Those words also have a
narrow range of phonotactic scores in order to prevent the effect of
phonotactic regularity on attaching meanings to word forms.

For creating a word list, we did not remove any participants based on
participants’ d-prime score because the list would be based on how
widely the words were known regardless of participants’ ability.

```
#word_cats <-read.csv("data_2402.csv")

#use_set <- defShort %>%
#  dplyr::select(word, item_dp, freq.cat, p.scr) %>%
#  distinct() %>%
#  mutate(dp_abs = abs(item_dp)) %>%
#  filter(dp_abs < 0.3) %>%
#  filter(p.scr > -0.89)

#pair_nums <- word_defs$pair.number[word_defs$item %in% use_set$word] %>% unique()

#use_pairs <- word_cats[word_cats$pair.number %in% pair_nums,]
#use_words <- use_pairs %>%
#  filter(type == "word") %>%
#  rowwise() %>%
#  mutate(
#    item_dp = use_set$item_dp[item == use_set$word],
#  )  %>%
#  ungroup()

#use_words <- use_words[sample(nrow(use_words)),]

#groups = rep(seq(1,3), times = length(use_words$word.freq)/3)
##groups <- c(groups, 1) # add extra integer
#use_words$group <- groups

#write.table(item.summary,row.names=FALSE, sep ="\t",file = "DT_item.summary.tsv")
```

```
# D-prime score for each participant
# portion of correct and  incorrect for "good" responses
# nb_responses_good<- defShort %>% dplyr::select (PID, enteredResponse, correct_def) %>% filter (enteredResponse == "good") %>% group_by(PID, correct_def) %>%  summarise (responses = n()) 

# d' = z(Hit-rate)-z(FA rate)
defShort<- defShort %>%
  group_by(PID) %>%
  mutate(
    hit= length(PID[correct_def == TRUE & enteredResponse == "good"])/length(PID[correct_def == TRUE]),
    FA = length(PID[correct_def == FALSE & enteredResponse == "good"])/length(PID[correct_def == FALSE]),
    PID_dprime = round(qnorm(hit) - qnorm(FA),3)
  )
```

### 2.2.3 Comparing Results in Two Different Definition Tasks (Descriptive Statistics)

To begin with, five participants who responded to more than 95% of
trials in the same way (e.g., “not good”) in our dataset were removed
from the data analysis. After the removal process, analyses were carried
out on 7300 observations of stimulus words with 50 participants.

```
#length(unique(defShort$PID)) #55

## accuracy/d-prime 
worker.summary<-defShort %>% dplyr::select(PID, PID_dprime, correct_def, accuracy = correct) %>% group_by(PID, correct_def) %>% mutate(accuracy_mean = mean(accuracy),accuracy_sd =sd(accuracy)) %>%  dplyr::select(-accuracy)%>%distinct() %>%
  ungroup()  

prop_answers <- table(defShort$PID, defShort$enteredResponse) %>%
  data.frame() %>%
  group_by(Var1) %>%
  mutate(Prop = Freq/sum(Freq))

## remove people who responded to more than 95% of trials in the same way (e.g., “not good”)
rm <- prop_answers %>% filter (Prop > 0.95)  %>% rename(PID = Var1)

## remove 5 participants 
defShort2<- defShort %>% filter(!PID %in% rm$PID)


# accuracy rate per item
defShort2 <- defShort2 %>% group_by(stimulus) %>% mutate(item_accuracy_mean = round(mean(correct),2),item_accuracy_sd = round(sd(correct), 2))

item.summary_50 <-defShort2  %>%group_by(stimulus) %>% dplyr::select(stimulus,item_dp, item_accuracy_mean, item_accuracy_sd, definition) %>% distinct() %>% ungroup()  
#summary(item.summary)

remove(prop_answers, worker.summary,rm)
#length(unique(defShort2$PID)) #50
```

For 12 words overlapping between the current study and Oh et al.’s
(2020), we report the extent to which the mean accuracy of each word is
correlated with each other from different tasks. In order to compare
directly between two different definition tasks, we adjusted the
accuracy scores of each item in the free definition task by 0.5\*free
definition accuracy + 0.5. This is because participants would be
expected to get it right 50% of the time just by random guessing in the
forced choice definition task.

Half of words have higher accuracy in the forced choice task, by up
to 10% compared with free definition accuracy. In a few cases, the two
situations give similar accuracy

```
# data from Oh et al.(2023)
items <- read.delim("exp1/nb_correct_Oh_Exp2.txt", sep ="\t", header = TRUE, encoding="UTF-8") %>% filter(word%in% defShort2$stimulus)%>%dplyr::rename(stimulus = word)

item.summary_dup<- item.summary_50[item.summary_50$stimulus%in% items$stimulus,] 
item.summary_dup<-left_join (item.summary_dup,items)  %>% dplyr::rename(
  "forced_exp_acc_mean" = "item_accuracy_mean", "free_response_acc_mean" = "rate") %>%  mutate(free_response_acc_adjusted = (free_response_acc_mean*0.5)+0.5) %>% dplyr::select (-2,-6)

# data for plots
forced <-item.summary_dup %>% dplyr::select (stimulus,forced_exp_acc_mean, definition) %>% dplyr::rename(acc_mean = forced_exp_acc_mean)
forced$task <- "forced"
free <-item.summary_dup %>% dplyr::select (stimulus, free_response_acc_adjusted, definition)%>% dplyr::rename(acc_mean = free_response_acc_adjusted)
free$task <- "free"

dup<-rbind(forced, free);dup$word <- paste0(dup$stimulus, " (", dup$definition, ")")

# vertical plot for the paper
ggplot(dup, aes(x = word, y = acc_mean)) + 
geom_line(size = 1) + 
geom_point(size = 5, aes(color = task)) + 
scale_x_discrete("") +
   scale_color_manual(values = c("forced" = "#FFA500", "free" = "#4682B4"))+
theme_minimal() +
   theme(text = element_text(size = 16)) +
   ylab('Proportion of correct definitions') + xlab('') + 
  theme(axis.text.x=element_text(angle = 70, hjust = 1, face="bold",size = 15))+
  theme(axis.text.y=element_text(face="bold",size = 15))+
  theme(panel.grid.major = element_line(color = "grey"))+
  scale_x_discrete(labels = function(x) str_wrap(x, width = 24))

#ggsave("./Figure 1-comparison.tiff", device = "tiff",width=10, height=8, dpi=600)
#ggsave(file="Figure 1-comparison.png", width=10, height=8, dpi=600)
remove(dup, forced, free, item.summary_50, items, item.summary_dup, defShort2, mp, hp, candidate)
```

The relationship between two tasks for the proportion of correct
responses for each word.

# 3 Experiment 2: Word Learning Experiment

## 3.1 Data Preparation

### 3.1.1 Word Stimuli

Our original set of stimuli generated for this experiment consists of
1054 tokens, made up of 527 pairs of real word (henceforth “word”) and
non-word pairs. These pairs were hand-selected from larger candidate
sets in order to meet various criteria. Each stimulus was assigned to
one of four bins:

- High Frequency (100+ per million)
- Mid Frequency (6-99 per million)
- Low Frequency (1-5 per million)
- Unattested (0 per million)

We selected 146 words from High and Mid frequency bins and conducted
a definition experiment with participants. A key point of the definition
task is to identify stimuli that participants can easily identify, and
filter these out for a learning task. In this experiment, for each
trial, participants saw a Māori word in the middle of the screen along
with its definition and decided whether the definition is good for the
word. Based on d-prime scores for each stimulus, we extracted 48 pairs
that do not have a wide range of phonotactic scores. The total set of
stimuli consists of

- 48 word/nonword pairs (96 items)
- 48 words (phonotactic score (-0.868 to -0.645)
- 48 nonwords (phonotactic score (-0.865 to -0.651)

```
#stimuli <- read_csv("tables/stimuli.csv")
#nonword <- read_csv("tables/nonword_set.csv")
#word <- read_csv("tables/word_set.csv")

# merge neighbourhood occupany rate from stimuli
#word <- merge(word, stimuli[, c("item", "poss.neighbors", "norm.neighbors")], by = "item", all.x = TRUE)
#write.table(word,row.names=FALSE, sep ="\t",file = "exp2/word_set.tsv")

#nonword <- merge(nonword, stimuli[, c("item", "poss.neighbors", "norm.neighbors")], by = "item", all.x = TRUE)
#write.table(nonword,row.names=FALSE, sep ="\t",file = "exp2/nonword_set.tsv")

word_set <- read.table("exp2/word_set.tsv", sep = "\t", header = TRUE)

#names(word_set)
summary_table_exp2_word <- word_set %>%
  summarise(
    Mean_Length = mean(n.phonemes, na.rm = TRUE),
    SD_Length = sd(n.phonemes, na.rm = TRUE),
    Mean_Phonotactic_Score = mean(score.shortv, na.rm = TRUE),
    SD_Phonotactic_Score = sd(score.shortv, na.rm = TRUE),
    Mean_Neighborhood_Density = mean(norm.neighbors, na.rm = TRUE),
    SD_Neighborhood_Density = sd(norm.neighbors, na.rm = TRUE)
  )  %>%
  mutate(across(where(is.numeric), ~ round(.x, 4)))

#print(summary_table_exp2_word)

nonword_set <- read.table("exp2/nonword_set.tsv", sep = "\t", header = TRUE)

#names(nonword_set)
summary_table_exp2_nonword <- nonword_set %>%
  summarise(
    Mean_Length = mean(n.phonemes, na.rm = TRUE),
    SD_Length = sd(n.phonemes, na.rm = TRUE),
    Mean_Phonotactic_Score = mean(score.shortv, na.rm = TRUE),
    SD_Phonotactic_Score = sd(score.shortv, na.rm = TRUE),
    Mean_Neighborhood_Density = mean(norm.neighbors, na.rm = TRUE),
    SD_Neighborhood_Density = sd(norm.neighbors, na.rm = TRUE)
  )  %>%
  mutate(across(where(is.numeric), ~ round(.x, 4)))

#print(summary_table_exp2_nonword)

remove(word_set, nonword_set, summary_table_exp2_word, summary_table_exp2_nonword)
```

### 3.1.2 Picture Stimuli

16 object pictures were selected from https://crl.ucsd.edu/experiments/ipnp/1database.html.
Conditions for searching pictures

- Syntactic category = object
- Number of alternative names = max 3
- RT target mean max = 1.5 sec
- Syllable max = 1

## 3.2 Creation of Stimuli

In the experiment, there were three experimental tasks (learning
phase, post-test phase 1, and post-test phase 2) as well as three
repetitions within each task. There were 16 picture stimuli, 48 real
words and 48 nonwords as mentioned in the previous section. Each
participant must add meaning to 16 word-form stimuli. Therefore, we
prepared 36 experiment configurations containing stimuli paired with
pictures. There were six random orders of the pictures in the
experiment. We also created six random samples of eight words and eight
nonwords. Each stimulus appears only once across the 6 word stimulus
samples (i.e., there are no repeat stimuli across the samples). A total
of 36 experimental configurations were used.

## 3.3 Experimental Method

The three-task experiment was conducted remotely and online, via a
standard web browser. It was posted on Prolific.co and recruited
non-Māori-speaking NZers and American participants separately.

Participants completed three tasks in a single sitting. At the
beginning of the experiment, participants were presented with a consent
form, explaining the basic details of the experiment. Following this,
they began the experiment. Each task included brief instructions at the
beginning for what they were expected to do.

### 3.3.1 Task 1

**Learning Phase: Identify the correct stimulus for a
given picture**

This task is to learn the names of objects in the language of the
experimental setting. Participants see a picture in the middle of the
screen along with two words, one of which is the correct name of the
pictured object. Participants are instructed to press the z key, if they
think the picture matches the word on the left side of the screen. They
are asked to press the m key, if they think the picture matches the word
on the right side of the screen. If they made the wrong choice, they
would see a red cross x in the middle of screen and if you responded
correctly, you would see a green tick ✔. If their decision takes too
long, a new item will be shown.

### 3.3.2 Task 2

**Test 1: Identify the correct picture for a given
stimulus**

For each trial, participants see a word in the middle of the screen,
then two pictures appear. The task is to decide which picture matches
the word by looking at the word and select the correct picture. If
participants think the left picture matches the word, then they press
the z key. If they think the right picture matches the word, then they
press the m key. They cannot receive any feedback for this phase.

### 3.3.3 Task 3

**Test 2: Answer whether the presented word-picture
pair is correct or incorrect**

For each trial, participants see a word and a picture. The word may
or may not be the correct label for the picture. Their task is to decide
whether the word and the object is either a correct or incorrect pair.
If participants think the pair is correct, press the m key. If
participants think the pair is incorrect, press the z key. They cannot
receive any feedback for this phase.

## 3.4 Data Cleaning Process and Filtering

### 3.4.1 Filtering Based on Survey Responses (NZ participants)

We used answers in the post-experiment questionnaire (see Section 5) to filter participants. 100
participants completed the three tasks and survey. The following
exclusions were made:

- 2 participants were removed for indicating they did not pay their
  attention to the experiment: 98 (not pressing the response keys (z or m)
  more than 10% of times in the experiment)
- 2 participants were removed because their IP addresses were
  identical: 96
- 8 participants were removed for indicating that they could speak
  or understand Māori at least “fairly well”: 88
- 2 participants were removed for indicating that they had studied
  Linguistics: 86
- 15 participants were removed for indicating that they had lived
  outside NZ for longer than a year since they were 7 and spending most of
  their life in overseas: 71
- 1 participant was removed for indicating that they lived in
  Hawaii: 70

There are 70 participants remaining after this filtering process.

```
#experiment<-read.table(file = 'dataNZPF.tsv', sep = '\t', header = TRUE)
#length(unique(experiment$jatosId)) #100
#table(experiment$type)

#Get the total number of responses per participant
# count None
#nb_res<- experiment %>% dplyr::select (workerId, count_keyboard_response) %>% distinct_all() %>% group_by(workerId)%>%  summarise (responses = n()) # 144 

# count None
#table(experiment$response)
#summaryResponse<-experiment%>% 
# group_by(jatosId,workerId,response) %>% dplyr::select(jatosId, response, #workerId)%>% add_count() %>% unique() %>% drop_na()

# remove people
#rm_worker <-summaryResponse%>%
#  group_by(workerId,response) %>%
#  filter(response == "None", n >= 14)

#experiment <- experiment %>%filter(! workerId%in% rm_worker$workerId) #2
#length(unique(experiment$jatosId)) #98

#remove(nb_res, rm_worker,summaryResponse)
#table(experiment$type)

## questionnaire
# reading surveys
#survey<-read.table(file = 'surveyNZPF.tsv', sep = '\t', header = TRUE) #100

# two attitude questions -> maoriAttitude
#survey$maoriRespect<- as.numeric(survey$maoriRespect)
#survey$maoriCompulsory <- as.numeric(survey$maoriCompulsory)
#survey$maoriAttitude <- #apply(survey[,c("maoriRespect","maoriCompulsory")],1,sum)
#table(survey$maoriAttitude)

# get people in the experimental data
#survey <- survey %>%filter(jatosId%in% experiment$jatosId) 
#length(unique(survey$jatosId)) #98

# get IP addresses that occur more than once
#repeat_ips2 <- survey$ipaddr %>% table() %>% data.frame() %>% filter(Freq > 1)
# 1ip freq =2 jatos Id,724,715
#survey<-survey %>%  group_by(ipaddr) %>% 
#  filter(!anyDuplicated(ipaddr))
#length(unique(survey$workerId)) #96

#experiment <- experiment [experiment$workerId%in%survey$workerId,]
#length(unique(experiment$workerId)) #96

#remove(repeat_ips2)

# speaking and understanding of Māori
#speakMaoriIDs <- unique(survey[survey$speakMaori >= 3,]$workerId) #1 worker IDs indicated that they spoke Māori "fairly well" or greater

#compMaoriIDs <- unique(survey[survey$compMaori >= 3,]$workerId) #9 worker IDs indicated that they understood Māori "fairly well" or greater (one of them in the speakMaoriIDs)

#experiment <- experiment[!experiment$workerId %in% speakMaoriIDs,]
#experiment <- experiment[!experiment$workerId %in% compMaoriIDs,]

#remove(speakMaoriIDs, compMaoriIDs)
#length(unique(experiment$workerId)) #88

# studying linguistics
#linguisticIDs <- unique(survey[survey$linguistics == "ling",]$workerId) # 2 worker IDs indicated that have studied linguistics
#experiment <- experiment[!experiment$workerId %in% linguisticIDs,]
#remove(linguisticIDs)
#length(unique(experiment$workerId)) #86

#outsideNZIDs <- unique(survey[survey$livingwithoutGap == "Yes",]$workerId) # 16 worker IDs indicated that have lived outside of New Zealand for longer than a year since they were 7
#rmoutsideNZIDs <- unique(survey[survey$livingwithoutGap == "Yes"& #survey$regionMost == "OVS",]$workerId) #2
# region most:What country or state participants spent the largest amount of time since they were 7
#regionMostIDs <- unique(survey[survey$regionMost == "OVS",]$workerId) #2

#experiment <- experiment[!experiment$workerId %in% outsideNZIDs,]
#length(unique(experiment$workerId)) #71
#experiment <- experiment[!experiment$workerId %in% rmoutsideNZIDs,]
#remove(outsideNZIDs,rmoutsideNZIDs)
#length(unique(experiment$workerId)) #71

# living in Hawaii
#hawaiiIDs <- unique(survey[survey$hawaii == "Yes",])$workerId # 1 worker ID
#experiment <- experiment[!experiment$workerId %in% hawaiiIDs,]
#remove(hawaiiIDs)
#length(unique(experiment$workerId)) #71

# polynesian language
#polyIDs <- unique(survey[survey$anyPolynesian == "Yes",])$workerId # 1 worker IDs
#experiment <- experiment[!experiment$workerId %in% polyIDs,]
#remove(polyIDs)
#length(unique(experiment$workerId)) #70

# low accuracy participants (meanACC from accuracy (i.e., =correct))
#PIDacc <- experiment %>%  filter (task != "1") %>% group_by(workerId) %>% mutate(meanAcc = mean(correct)) %>% subset(select=c(workerId, meanAcc)) %>% unique()
#meanSd <- sd(PIDacc$meanAcc)

#summary(PIDacc)

#twoSdCutoff <- (mean(PIDacc$meanAcc) - meanSd) - meanSd  #0.502 (two participant)
#lowIDs <- PIDacc$workerId[PIDacc$meanAcc < twoSdCutoff] #0

#threeSdCutoff <- (mean(PIDacc$meanAcc) - meanSd) - 2*meanSd #0.3658
#lowIDs <- PIDacc$workerId[PIDacc$meanAcc < threeSdCutoff] #0

#twoSdCutoff <- (mean(PIDmedians$medianAcc) - medianSd) - medianSd #50.82 (two participant)
#threeSdCutoff <- (mean(PIDmedians$medianAcc) - medianSd) - 2*medianSd #36.71
#lowIDs <- PIDmedians$workerId[PIDmedians$medianAcc < threeSdCutoff] #0

# low accuracy participants (based on accuracy column from OSWeb)
#PIDmedians <- experiment %>%  filter (task != "1") %>% group_by(workerId) %>% mutate(medianAcc = median(accuracy)) %>% subset(select=c(workerId,medianAcc)) %>% unique()
#medianSd <- sd(PIDmedians$medianAcc)
#twoSdCutoff <- (mean(PIDmedians$medianAcc) - medianSd) - medianSd #50.82 (two participant)
#threeSdCutoff <- (mean(PIDmedians$medianAcc) - medianSd) - 2*medianSd #36.71
#lowIDs <- PIDmedians$workerId[PIDmedians$medianAcc < threeSdCutoff] #0

#experiment <- experiment[!experiment$workerId %in% lowIDs,]
#remove(PIDmedians, medianSd, twoSdCutoff, threeSdCutoff, lowIDs)

#length(unique(experiment$workerId)) #70

# remaining survey data
#experiment <- experiment[experiment$workerId %in% survey$workerId,] 
#length(unique(experiment$workerId)) #70

# survey data
#survey <- survey[survey$workerId %in% experiment$workerId,] # 70

#table(survey$age)
#table(survey$gender)

#table(experiment$subject_nr )# #34sets/36sets (no 19,21,28)
#table(experiment$type) #5040/5040

#a <- survey %>% ungroup() %>% dplyr::select (jatosId, age,gender,education,maoriProf,maoriExpo,maoListY,maoriAttitude) %>%  replace(is.na(.), 0 )%>% mutate(newCol = "NZ") %>% rename("group" = "newCol")

#WordLearningNZPF70<-right_join(experiment, a, by="jatosId") %>% dplyr::select (-group.y) %>% rename("group" = "group.x")

# save as a tsv.file 
#write.table(WordLearningNZPF70,row.names=FALSE, sep ="\t",file = "docs1/WordLearningNZPF70.tsv")
#write.table(a,row.names=FALSE, sep ="\t",file = "docs1/demographicNZ70.tsv") 

# id
#NzId<-survey %>% ungroup() %>% dplyr::select (workerId,jatosId) %>% distinct()
# save as a tsv.file 
#write.table(NzId,row.names=FALSE, sep ="\t",file = "docs1/NzId70.tsv")
```

### 3.4.2 Filtering Based on Survey Responses (US participants)

78 participants completed the three tasks and survey

- 1 participant was removed for indicating that the person did not
  pay his/her attention to the experiment (not pressing the response keys
  (z or m) more than 10% of times in the experiment): 77
- 3 participants were removed because their IP addresses were
  identical: 74
- 2 participants were removed for indicating that they had studied
  Linguistics: 72
- 5 participants were removed for indicating that they had lived
  outside USA for longer than a year since they were 7 and spending most
  of their life in overseas: 67

There are 67 participants remaining after this filtering process.

```
#experimentA<-read.table(file = 'dataUSA.tsv', sep = '\t', header = TRUE)
#length(unique(experimentA$jatosId)) #78

# Get the total number of responses per participant
#nb_res <- aggregate(experimentA$count_keyboard_response, by=list(experimentA$jatosId),  FUN=length)
#unique(nb_res$x) 
#names(nb_res)<-c("jstosId", "responses")

# count None
#table(experimentA$response)
#summaryResponse<-experimentA %>% group_by(jatosId,workerId,response) %>% dplyr::select(jatosId, response, workerId)%>% add_count() %>% unique()  %>% drop_na()

# remove people
#rm_worker <-summaryResponse%>% group_by(workerId,response) %>% filter(response == "None", n >= 14)

#experimentA <- experimentA %>%filter(! workerId%in% rm_worker$workerId) #1
#length(unique(experimentA$jatosId)) #77

#remove(nb_res,rm_worker,summaryResponse)

## questionnaire
# reading surveys
#survey2<-read.table(file = 'surveyUSA.tsv', sep = '\t', header = TRUE) #78

# get people in the experimental file
#survey2 <- survey2 %>%filter(jatosId%in% experimentA$jatosId) 
#length(unique(survey2$jatosId)) #77

# get IP addresses that occur more than once
#repeat_ips <- survey2$ipaddr %>% table() %>% data.frame() %>% filter(Freq > 1)
# 3 different Prolific IDs (all female sisters?) the same ips as the repeated ip in NZ
# 1ip freq =3 jatos Id, 601,724,715 all of them: Māori education 1
#survey2<- survey2%>%  group_by(ipaddr) %>%  filter(!anyDuplicated(ipaddr))
#length(unique(survey2$workerId)) #74

#experimentA <- experimentA %>%filter(workerId%in% survey2$workerId) 
#length(unique(experimentA$workerId)) #74

#remove(repeat_ips)

#length(unique(experimentA$workerId)) #74
#table(experimentA$type)

# speaking and understanding of Māori
#speakMaoriIDs <- unique(survey2[survey2$speakMaori >= 3,]$workerId) #0 worker IDs indicated that they spoke Māori "fairly well" or greater
#compMaoriIDs <- unique(survey2[survey2$compMaori >= 3,]$workerId) #0 worker IDs indicated that they understood Māori "fairly well" or greater

#remove(speakMaoriIDs, compMaoriIDs)
#length(unique(experimentA$workerId)) #74

# studying linguistics
#linguisticIDs <- unique(survey2[survey2$linguistics == "ling",]$workerId) # 2 worker IDs indicated that have studied linguistics
#experimentA <- experimentA[!experimentA$workerId %in% linguisticIDs,]
#remove(linguisticIDs)
#length(unique(experimentA$workerId)) #72

# lived outside of USA longer than one year
#outsideUSIDs <- unique(survey2[survey2$livingwithoutGap == "Yes",]$workerId) # 4 worker IDs indicated that have lived outside of USA for longer than a year since they were 7
#rmoutsideUSIDs <- unique(survey2[survey2$livingwithoutGap == "Yes"& survey2$regionMost == "Other",]$workerId) #2
# region most:What country or state participants spent the largest amount of time since they were 7
#regionMostIDs <- unique(survey2[survey2$regionMost == "Other",]$workerId) #3

#experimentA <- experimentA[!experimentA$workerId %in% outsideUSIDs,]
#length(unique(experimentA$workerId)) #68
#experimentA <- experimentA[!experimentA$workerId %in% regionMostIDs,]
#remove(outsideUSIDs,rmoutsideUSIDs, regionMostIDs)
#length(unique(experimentA$workerId)) #67

# living in Hawaii
#hawaiiIDs <- unique(survey2[survey2$hawaii == "Yes",])$workerId # 0 worker ID
#remove(hawaiiIDs)
#length(unique(experimentA$workerId)) #67

# Polynesian language
#polyIDs <- unique(survey2[survey2$anyPolynesian == "Yes",])$workerId # 0 worker IDs
#remove(polyIDs)

# low accuracy participants
#PIDmedians <- experimentA %>%  filter (task != "1") %>% group_by(workerId) %>% mutate(medianAcc = median(accuracy)) #%>% subset(select=c(workerId,medianAcc)) %>% unique()
#medianSd <- sd(PIDmedians$medianAcc)
#twoSdCutoff <- (mean(PIDmedians$medianAcc) - medianSd) - medianSd #46.73 two participants
#threeSdCutoff <- (mean(PIDmedians$medianAcc) - medianSd) - 2*medianSd #33.81
#lowIDs <- PIDmedians$workerId[PIDmedians$medianAcc < threeSdCutoff] #0

#remove(PIDmedians, medianSd, twoSdCutoff, threeSdCutoff, lowIDs)
#length(unique(experimentA$workerId)) #67
#table(experimentA$type)

# remaining survey data
#eperimentA <- experimentA[experimentA$workerId %in% survey2$workerId,] 
#length(unique(experimentA$workerId)) #67

# survey data
#survey2 <- survey2[survey2$workerId %in% experimentA$workerId,] # 67
#length(unique(experimentA$workerId)) #67

#table(survey2$gender)
#table(survey2$age)

#ageOver60 <- unique(survey2[survey2$age == "+60",])$jatosId # 4 worker ID
#[1] 357 350 285 282

#ageOver60 <- unique(survey2[survey2$age == "+60",])$workerId # 4 worker ID
##experimentA_under60 <- experimentA[!experimentA$workerId %in% ageOver60,]
##length(unique(experimentA_under60$workerId)) #65
#remove(ageOver60)

#survey2 <- survey2[survey2$workerId %in% experimentA$workerId,] 
#length(unique(experimentA$workerId)) #67

#b <- survey2 %>% ungroup() %>% dplyr::select (jatosId, #age,gender,education,maoriProf,maoriExpo,maoListY,otherLang, numLangs) %>%  replace(is.na(.), 0 )%>% #mutate(newCol = "US") %>% rename("group" = "newCol")

#WordLearningUSA67<-merge(experimentA, b, by="jatosId") %>% dplyr::select (-group.y) %>% rename("group" = "group.x")

# save as a tsv.file 
#write.table(WordLearningUSA67,row.names=FALSE, sep ="\t",file = "docs1/WordLearningUSA67.tsv")

# id
#UsId<- WordLearningUSA67 %>% dplyr::select (workerId,jatosId) %>% distinct()
# save as a tsv.file 
#write.table(UsId,row.names=FALSE, sep ="\t",file = "UsId67.tsv")

# bind NZ & US data
#data<-rbind(experiment,experimentA)
#data %>% dplyr::select(jatosId,group) %>% distinct_all () %>% group_by(group) %>% #summarise(n())  # okay
#length(unique(data$jatosId)) #137
# table(data$type) #nonword 9864   word 9864

# Demographic data from Survey
# NZ
#demographicNZ <- a %>%  dplyr::select (-maoriAttitude)

# USA
#demographicUSA <-b 

# merge two sets
#demographic<-rbind(demographicNZ,demographicUSA)
#write.table(demographic,row.names=FALSE, sep ="\t",file = "docs1/demographicNZUSPF137.tsv")

#names(data)
#Data<- data %>% dplyr::select (-1,-6,-17) %>% dplyr::rename("PID" = "jatosId")# change jatosId to PID

#remove(survey, survey2)
```

## 3.5 Demographic Data

Finally, we merge the datasets. The resulting datasets are the full
datasets used for statistical analysis. We have 70 NZ participants (36
female) and 67 US Participants (31 female) for a total of 137
participants. All of them are native speakers of English and 18 years or
older. They are not able to hold a basic conversation in te reo Māori.
Some results of our post experimental survey (see Section 5) for the participants are shown
below.

Participants’ exposure levels are based on participants’ responses to
the two questions “How often do you think you are exposed to the Māori
language in your daily life, by means of Māori radio, Māori TV, online
media?” & “How often do you think you are exposed to Māori language
in your daily life, in conversation at work, at home, in social
settings?”, using a scale ranging from 1 “Less than once a year” to 5
“Multiple times a day”. These options were coded from 1 to 5, for a
total Māori exposure score from 2 to 10. NZ participants (mean = 5.75)
were generally exposed to Māori at least once a month, whereas US
participants (mean = 2.01) were exposed to Māori less than once a
year.

Participants generally rated themselves poorly at understanding or
speaking Māori. “How well are you able to speak Māori?” & “How well
are you able to understand/read Māori” (rating from “Not at all” to
“very well”, with a scale of 6 options. These options were coded from 0
to 5 and were combined for a Māori proficiency score from 0 to 10. Most
participants stated that they are able to speak/understand “no more than
a few words for phrases” (scoring 2 or below) in Māori proficiency.
However, NZ participants did have some basic knowledge of Māori (such as
songs, basic numerals etc.)

Regarding other language experience, among NMS participants, 81.4%
were monolingual (reporting “none”), while 2.9% reported speaking two
additional languages and 15.7% one additional language. For US
participants, 82.1% were monolingual, 4.5% reported speaking two
additional languages, and 13.4% one additional language. Examples of
additional languages include Japanese, French, and Spanish.

```
data<-read.table('exp2/demographicNZUSPF137.tsv', sep = '\t', header = TRUE) %>% dplyr::rename("basic_knowledge" = "maoListY", "edu" = "education", "exposure" = "maoriExpo","proficiency" = "maoriProf", "PID" = "jatosId")

# Population and Set Bar Graph
#table(data$group)
fig.all<-ggplot(data, aes(x=factor(group), fill=factor(group)))+
  geom_bar(aes(),size=.8,position="dodge",show.legend=FALSE) + 
   geom_text(aes(label = after_stat(count)),
    stat = "count", vjust=.9, size=3) +
  scale_fill_hue("group", direction = -1)+
   xlab("Group") +
  ylab('No. of People') #+ ggtitle("Population")
#fig.all

# Age and Set Bar Graph
#table(data$age,data$group)
# change levels
data$age<- factor(data$age, levels=c("18-29","30-39","40-49","50-59","+60"))

fig.age<-ggplot(data, aes(x=factor(age), fill=factor(group)))+
  geom_bar(aes(),size=.8,position="dodge",show.legend=F) + 
    geom_text(stat='count', aes(label = after_stat(count)), vjust=.3, size=3, position = position_dodge(.9)
  )+
    scale_fill_hue("group", direction = -1)+
  xlab("Age") +
  ylab('No. of People')
#fig.age

# Gender and Set 
#table(data$gender, data$group)
fig.gender<-ggplot(data, aes(x=factor(gender), fill=factor(group)))+
  geom_bar(aes(),size=.8,position="dodge",show.legend=F) + 
  geom_text(stat='count', aes(label = after_stat(count)), vjust=.3, size=3,position = position_dodge(.9)
  )+
    scale_fill_hue("group", direction = -1)+
  xlab("Gender")+
  ylab('No. of People')
#fig.gender

# Highest education
#table(data$edu, data$group)
data<-data %>%
  mutate(education = dplyr::recode(edu, '2' = 'high school', '3' = 'cert./diploma', '4' = 'undergrad', '5' = 'graduate')) 

# change levels
data$education<- factor(data$education, levels=c("high school","cert./diploma","undergrad","graduate"))

fig.edu<-ggplot(data, aes(x=factor(education), fill=factor(group)))+
  geom_bar(aes(),size=.8,position="dodge",show.legend=F) + 
  geom_text(stat='count', aes(label = after_stat(count)), vjust=.3, size=3,position = position_dodge(.9)
  )+
    scale_fill_hue("group", direction = -1)+
  xlab("Highest education")+
  ylab('No. of People')
#fig.edu

# Māori proficiency
#table(data$proficiency, data$group)
fig.prof<-ggplot(data, aes(x=factor(proficiency), fill=factor(group)))+
  geom_bar(aes(),size=.8,position="dodge",show.legend=F) + 
  geom_text(stat='count', aes(label = after_stat(count)), vjust=.3, size=3,position = position_dodge(.9)
  )+
    scale_fill_hue("group", direction = -1)+
  xlab("Māori proficiency")+
  ylab('No. of People')
#fig.prof

# Basic Knowledge of Māori
#table(data$basic_knowledge, data$group)
fig.knowledge<-ggplot(data, aes(x=factor(basic_knowledge), fill=factor(group)))+
  geom_bar(aes(),size=.8,position="dodge",show.legend=F) + 
  geom_text(stat='count', aes(label = after_stat(count)), vjust=.3, size=3,position = position_dodge(.9)
  )+
    scale_fill_hue("group", direction = -1)+
  xlab("Basic knowledge of Māori")+
  ylab('No. of People')
#fig.knowledge

# Māori exposure
#table(data$exposure, data$group)
fig.expo<-ggplot(data, aes(x=factor(exposure), fill=factor(group)))+
  geom_bar(aes(),size=.8,position="dodge",show.legend=F) + 
  geom_text(stat='count', aes(label = after_stat(count)), vjust=.3, size=3,position = position_dodge(.9)
  )+
    scale_fill_hue("group", direction = -1)+
  xlab("Māori exposure")+
  ylab('No. of People')
#fig.expo

# mean_maoriExpo<-data %>% dplyr::select(exposure,group) %>% group_by (group) %>% dplyr::summarize(Mean = mean(exposure),  sd=sd(exposure))1
#NZ 5.75 US 2.01

# Other languages
# Convert numLanguages to a factor and make sure 'None' is included
data$numLangs <- factor(data$numLangs, levels = c("None", "1", "2"))

fig.otherLang <-ggplot(data, aes(x=factor(numLangs), fill=factor(group)))+
  geom_bar(aes(),size=.8,position="dodge",show.legend=F) + 
  geom_text(stat='count', aes(label = after_stat(count)), vjust=.3, size=3,position = position_dodge(.9)
  )+
    scale_fill_hue("group", direction = -1)+
  xlab("Other languages")+
  ylab('No. of People')
#fig.otherLang

ggarrange(fig.all, fig.age, fig.gender,fig.edu, fig.expo,fig.prof,fig.knowledge, fig.otherLang)
remove(fig.all, fig.age, fig.gender,fig.edu, fig.expo,fig.prof,fig.knowledge,fig.otherLang)
```

Overview of participants’ demographics in Experiment 2

## 3.6 Statistical Analysis

We analysed the data using mixed-effects regression (logistic), with
an alpha level of .05 for all statistical tests. Predictors included
linguistic and non-linguistic factors, described below in the analysis
of each model. Categorical predictors were treatment-, sum- or reverse
Helmert- coded as appropriate, and numerical predictors were centered.
All models included random intercepts for participant and stimulus, with
by-participant random slopes for linguistic predictors and by-stimulus
random slopes for non-linguistic predictors, if models are
converged.

In the fixed-effects setting, we remove interactions one at a time if
they do not make a significant contribution to the model’s explanatory
power. We then remove main effects if they are not included in any
remaining interactions and if they are not statistically significant (p
> .05)

Once there are no more candidate terms for removal, we re-fit the
model in a mixed-effects setting, adding random intercepts by
participant and item and random slopes by participant for each remaining
term. We remove interaction terms from random slopes as required to
circumvent problems in running the models. We identify candidate terms
for removal if their coefficient is not significant in the model; after
performing each removal, we confirm that it was justified via model
comparison, using a log-likelihood ratio test.

We conducted post-hoc estimated marginal means (EMM) tests using the
R package emmeans (Lenth et al., 2022) for the interpretation of
significant interactions.

## 3.7 Experimental Results

The experiment dataset is structured as follows:

Dependent Variable

- *accuracy* is binary, either the participants’ response is
  correct (TRUE) or incorrect (FALSE).

Independent Variables

- *group* (Sum-coded) is the nationality group for each
  participant. US [1] NZ [-1]
- *type* (treatment-coded) is the type of stimulus (word
  vs. nonword [reference]).
- *task* (Helmert-coded) is the type of task. 1vs2-3: Task 1
  (learning phase) vs. Task 1 and Task 2 and Task 3 (two test phases).
  2vs3: Task 2 (the first test phase) vs. Task 3 (the second test
  phase).
- *education* (treatment-coded) is each participant’s highest
  level of education: “high school” [reference], “cert./diploma”,
  “undergrad”, “graduate”.

Random Effects

- *PID* is the unique ID for each participant.
- *word* is the stimulus used for the rating.

When the interactions were found, we tested post-hoc estimated
marginal means (EMM) tests.

### 3.7.1 Summary of raw data 1

Participants learnt well in task 2 and 3 regardless the type of
stimuli (i.e., word or nonword).

```
Data<-read.table('exp2/WordLearningNZUSPF137.tsv', sep = '\t', header = TRUE)

Data<-left_join(Data, data)

#length(unique(Data$PID)) #137 (PID = original jatosId)
#nb_res <- aggregate(Data$count_keyboard_response, by=list(Data$PID),  FUN=length) #144 per participant
#table(Data$type)
#table(Data$subject_nr) #all
#table(Data$response)

# summary Type, Task and Group

group.summary <- Data %>%
  group_by(type, task, group) %>%
  summarise(
    mean = mean(correct, na.rm = TRUE),
    sd = sd(correct, na.rm = TRUE),
    n = n(),
    .groups = "drop"
  )

# summary Type, Task and Group
#participant.summary<-Data %>% group_by(type,task, group,PID) %>% dplyr::select(type, task, PID,group,accuracy=correct) %>% summarise(mean=mean(accuracy), sd=sd(accuracy))

# summary Type, Task and Group
#item.summary<-Data %>%   group_by(stimuli,type,task, group) %>% dplyr::select(stimuli,type, task,group, accuracy=correct)%>% summarise(mean=mean(accuracy), sd=sd(accuracy)) %>%   mutate(Diff = mean- lag(mean)) %>% mutate(across(mean:Diff) * 100) %>% mutate(across(where(is.numeric), round, 1))

#item.summary2<-Data %>% group_by(stimuli,type,task, group) %>% dplyr::select(stimuli,type, task,group, accuracy=correct)%>% filter(task%in% c("2","3")) %>%summarise(mean=mean(accuracy), sd=sd(accuracy)) %>%    mutate(Diff = mean- lag(mean)) %>% mutate(across(mean:Diff) * 100) %>% mutate(across(where(is.numeric), round, 1))

#item.summary.Task2<-Data %>% group_by(stimuli,type,task, group) %>% dplyr::select(stimuli,type, task,group, accuracy=correct)%>% filter(task%in% c("2")) %>%summarise(mean=mean(accuracy), sd=sd(accuracy)) %>%  mutate(Diff = mean- lag(mean)) %>% mutate(across(mean:Diff) * 100) %>% mutate(across(where(is.numeric), round, 1))

#Get the total number of responses per item
#item<-Data %>% count(group,stimuli)
#summary(item) # min 81 (9 participants per item), max 135(15 participants per item)
remove(data, group.summary)
```

```
Data$word = as.character(Data$word)
Encoding(Data$word) = "UTF-8"

Data$task <- as.factor(Data$task)
Data$type <- as.factor(Data$type)
Data$group<- as.factor(Data$group)

#length(unique(Data$PID)) #137

#Get mean ratings for each stimulus per bin
allTasks <- list()
task_names <- c("1", "2","3")
for(i in 1:length(task_names)){
  Task<- Data[Data$task==task_names[i],]
  TaskMeanAcc <- aggregate(as.numeric(Task$correct), by=list(Task$word, Task$type, Task$group), mean)
  names(TaskMeanAcc) <- c("item","type","group","meanAcc")
  TaskMeanAcc$task <- task_names[i]
  allTasks[[i]] <-TaskMeanAcc
}
remove(i, Task, TaskMeanAcc)

allTasks <- do.call(rbind,allTasks)
allTasks$type = factor(allTasks$type)
levels(allTasks$type) = c("Nonword", "Word")
allTasks$task <- factor(allTasks$task, levels=c("1","2","3"))

# Get grand mean ratings for each stimulus type per bin
taskMeans <- allTasks %>% 
  group_by(type, task, group) %>% 
  dplyr::summarize(grand_mean = mean(meanAcc))
```

```
ggplot(allTasks, aes(x=group, y=meanAcc, color=type)) +
  geom_point(aes(shape=type), alpha=0.3, size=3) +
  geom_smooth(aes(group=type, fill=type), method="lm", formula="y~x", alpha=0.6, size=1)+
  facet_grid(. ~ task) +
  xlab("group") +
  ylab("Mean accuracy (per stimulus)") +
  scale_shape_manual(name="Stimulus type",
                    values = c("Nonword" = 1, "Word" = 2),
                    guide = guide_legend(reverse = TRUE)) +   
  scale_color_manual(name="Stimulus type",
                     values = c("Nonword"="black", "Word" = "blue"),
                     guide = guide_legend(reverse = TRUE)) +
  scale_fill_manual(name="Stimulus type",
                   values = c("Nonword"="grey70", "Word" = "dodgerblue1"),
                   guide = guide_legend(reverse = TRUE)) +  
  theme_bw() + 
  theme(
    panel.grid = element_blank()
  )

remove(allTasks, taskMeans)
```

Mean accuracy of each group for each stimulus for real words and
non-words across tasks.

The plots show the accuracy rate (proportion correct reposes) for
each stimulus across the groups by education, phonotactic score,
neighborhood occupancy rate across, respectively in Task1, Task2 and
task3. It is clear that participants’ responses were not influenced by
these three variables.

```
#length(unique(Data$PID)) #137 (PID = original jatosId)
#nb_res <- aggregate(Data$count_keyboard_response, by=list(Data$PID),  FUN=length) #144 per participant
#table(Data$type)
#table(Data$subject_nr) #all
#table(Data$response)

## summary Type, Task and Group
#group.summary<-Data %>% group_by(type,task,group) %>% dplyr::select(type, task, group, accuracy=correct) %>% summarise(mean=mean(accuracy), sd=sd(accuracy))

# summary Type, Task and Group
#participant.summary<-Data %>% group_by(type,task, group,PID) %>% dplyr::select(type, task, PID,group,accuracy=correct) %>% summarise(mean=mean(accuracy), sd=sd(accuracy))

# summary Type, Task and Group
#item.summary<-Data %>%   group_by(stimuli,type,task, group) %>% dplyr::select(stimuli,type, task,group, accuracy=correct)%>% summarise(mean=mean(accuracy), sd=sd(accuracy)) %>%   mutate(Diff = mean- lag(mean)) %>% mutate(across(mean:Diff) * 100) %>% mutate(across(where(is.numeric), round, 1))

#Get the total number of responses per item
#item<-Data %>% count(group,stimuli)
#summary(item) # min 81 (9 participants per item), max 135(15 participants per item)

# education
accuracy<-Data %>% dplyr::select(correct, task, group, education) %>% 
  #filter(task %in% c("2","3")) %>% 
  group_by(task, group, education) #%>% summarise_each(funs(mean, sd))

edu_plot <-accuracy %>% group_by(group, education) %>% 
  dplyr::summarize(Accuracy = mean(correct)) %>% 
  ggplot(aes(x = education, y = Accuracy, fill = group)) + 
  geom_col() +
  facet_wrap(.~group, nrow = 1, ncol = 4, scales = "free") +
  theme_bw() +
   scale_fill_hue("group", direction = -1)+
  theme(axis.text.x = element_text(angle = 45, hjust = 1),
    plot.title = element_text(size = 10, face = "bold"))  +    
    scale_y_continuous(labels = scales::percent) 
edu_plot
```

Mean accuracy of each group by education.

```
# the effect of norm.neighbors: none
nor_plot = Data %>% dplyr::select(correct, type, task, group, norm.neighbors)%>%
  #filter(task%in% c("2","3")) %>% 
  group_by(group,norm.neighbors, type, task) %>% dplyr::summarize(
    mean_correct = mean(as.numeric(correct)),
    n = n()
  ) %>% mutate(group = fct_relevel(group, c("US","NZ")))%>%
  ungroup() %>%
   ggplot(., aes(x=norm.neighbors, y=mean_correct, color=group)) +
    geom_point(aes(shape=group), alpha=0.3, size=4) +
    geom_smooth(aes(fill=group), method="lm", formula="y~x", alpha=0.6, size=1)+
    facet_grid(type~task, labeller="label_both", switch="y")+ 
  scale_x_continuous(breaks=seq(0,0.25,by=0.10)) +
     xlab("Neighborhood occupancy rate") +
  ylab("Accuracy rate")
       
nor_plot
```

Mean accuracy of each group for each stimulus for real words and
non-words across tasks and their relation to neighborhood occupancy
rate.

```
# the effect of phonotactic.scores: none
p.scr_plot = Data %>% dplyr::select(correct, type, task, group, score.shortv) %>% 
  #filter(task%in% c("2","3")) %>%
  group_by(group,score.shortv, type, task) %>% 
  dplyr::summarize(
    mean_correct = mean(as.numeric(correct)),
    n = n()
  )  %>% mutate(group = fct_relevel(group, c("US","NZ")))%>%
  ungroup() %>%
   ggplot(., aes(x=score.shortv, y=mean_correct, color=group)) +
    geom_point(aes(shape=group), alpha=0.3, size=4) +
    geom_smooth(aes(fill=group), method="lm", formula="y~x", alpha=0.6, size=1)+
    facet_grid(type~task, labeller="label_both", switch="y")+ 
  scale_x_continuous(breaks=seq(-0.85,-0.65,by=0.10)) +
  xlab("Phonotactic score") +
  ylab("Accuracy rate") 

p.scr_plot
#ggarrange(nor_plot, p.scr_plot, edu_plot, cols = 2)

remove(accuracy, edu_plot, nor_plot, p.scr_plot)
```

Mean accuracy of each group for each stimulus for real words and
non-words across tasks and their relation to phonotactic score.

### 3.7.2 Logistic Regression Model with a variable stimulus type

For the current study, the datasets of two groups were merged. We
analysed the data using logistic regression in order to test the
hypotheses. We set out a model with a three-way interaction between
`group` (US, NZ), `task` (1,2,3) and
`type` (words, non-words). We added `education` as
a control predictor.

Hypotheses

- 1. Non-Māori-speaking New Zealanders (NMS) will learn the meanings of
     the real words more accurately than United States participants
- 2. Non-Māori-speaking New Zealanders will learn the meanings of the
     real words more accurately than the meanings of the non-words.
- 3. The United States participants will show no differences in the way
     they learn the meanings of real words and non-words

The summary of the model is shown below, together with partial effect
plots. Contrary to our prediction, we did not observe an interaction
between stimulus type and group. That is, although NMS generally learned
new meanings more accurately than non-New Zealanders, this advantage was
not focused on their learning of meanings for words that were assumed to
already exist in their proto-lexicon. Rather, it appears that each group
learned the meanings for nonwords as well as they did for words, and NMS
were stably better than non-NZers at learning the meanings for both.
Consequently, our prediction that NMS would learn the meanings of real
words easier than nonwords was not supported by the experimental
evidence.

```
#str(Data)

## Convert  1 and 0 TRUE/FALSE to
Data$accuracy <- as.logical(as.integer(Data$correct))
#Data$correct<-as.factor(Data$correct)
Data$word<-as.factor(Data$word)
Data$PID<-as.factor(Data$PID) 
Data$group<-as.factor(Data$group)
Data$task<-as.factor(Data$task)
Data$type<-as.factor(Data$type)
Data$education<-as.factor(Data$education)

##  task * group * type

## The random effects structure was simplified as necessary by pruning random slopes one at a time until the model converged.
## all tasks

## Sum coding for the group
Data$group<- factor(Data$group, levels=c("US","NZ"))

Data$group.S. <- as.factor(Data$group)
#levels(Data$group.S.) #[1] "US" "NZ"
sumC<- contr.sum(2)
colnames(sumC) <- c("US") # 1 (US), -1(NZ)

contrasts(Data$group.S.) = sumC
#contrasts(Data$group.S.)

remove(sumC)

## Helmert coding-CC
## first comparison training (1) vs. test (2&3), 
## second comparison between tests (task 2 vs.task3)

Data$task.HC.<- factor(Data$task, levels=c("1","2","3"))
my.HC = matrix(c(2/3, -1/3, -1/3, 0, .5, -.5), ncol = 2)
#my.HC

colnames(my.HC) <- c("1vs2-3", "2vs3")
contrasts(Data$task.HC.) = my.HC
#contrasts(Data$task.HC.)

#str(Data)

## fixed-effects setting,intercepts only

#m1type <-  glmer(accuracy ~ group.S.*type*task.HC.+education +(1|PID)+(1|word), family= binomial, data=Data,control=glmerControl(optimizer="bobyqa", optCtrl=list(maxfun=2e+05)))
#summary(m1type) # Removal candidate: group.S.:type:task.HC.
#m2type <- update(m1type, . ~ . - group.S.:type:task.HC.)
#anova(m1type, m2type, test="LRT") # Removal justified Pr(>Chisq 0.7434
#summary(m2type) # Removal candidate: group.S.:type 
#m3type <- update(m2type, . ~ . - group.S.:type)
#anova(m2type, m3type, test="LRT") # Removal justified Pr(>Chisq 0.7493
#summary(m3type) # Removal candidate: type:task.HC.
#m4type <- update(m3type, . ~ . - type:task.HC.)
#anova(m3type, m4type, test="LRT") # Removal justified Pr(>Chisq  0.7401
#summary(m4type) # 
#m5type <- update(m4type, . ~ . - education)
#anova(m4type, m5type, test="LRT") # Removal justified Pr(>Chisq 0.6892
#summary(m5type) # no removal candidate

## mixed-effects setting: add by-participant and by-word random slopes
#m6type<- glmer(accuracy ~ group.S.*task.HC.+type+(1+type|PID)+(1+group.S.+task.HC.|word), family= binomial, data=Data,control=glmerControl(optimizer="bobyqa", optCtrl=list(maxfun=2e+05)))
#summary(m6type)
#saveRDS(m6type, file = "docs_exp2/m6type.rds") 

#m7type<- glmer(accuracy ~ group.S.*task.HC.+(1|PID)+(1+group.S.+task.HC.|word), family= binomial, data=Data,control=glmerControl(optimizer="bobyqa", optCtrl=list(maxfun=2e+05)))
#summary(m7type)
#saveRDS(m7type, file = "docs_exp2/m7type.rds") 

m6type<- readRDS("exp2/m6type.rds")
logistic_table(m6type)

remove(my.HC)
```

| Parameter | Estimate | Std. Error | z | p |  |
| --- | --- | --- | --- | --- | --- |
| (Intercept) | 0.932 | 0.074 | 12.673 | <0.001 | \*\*\* |
| group.S. US | -0.138 | 0.053 | -2.576 | 0.010 | \*\* |
| task.HC. 1vs2-3 | -0.854 | 0.065 | -13.038 | <0.001 | \*\*\* |
| task.HC. 2vs3 | -0.177 | 0.097 | -1.832 | 0.067 | . |
| type word | 0.109 | 0.067 | 1.634 | 0.102 |  |
| group.S.US : task.HC. 1vs2-3 | 0.170 | 0.034 | 4.952 | <0.001 | \*\*\* |
| group.S.US : task.HC. 2vs3 | -0.075 | 0.043 | -1.724 | 0.085 | . |

```
m6type<- readRDS("exp2/m6type.rds")
emm_g.t<-emmeans(m6type, revpairwise ~ group.S.|task.HC., infer =TRUE)

summary(emm_g.t$emmeans) %>%  mutate(across(where(is.numeric), round, 3)) %>% mutate(
    significance = case_when(
      p.value < 0.001 ~ "***",
      p.value < 0.01 ~ "**",
      p.value < 0.05 ~ "\\*",
      p.value < 0.1 ~ ".",
     TRUE ~ ""),
    p.value = ifelse(p.value<0.001, "<0.001", format(round(p.value, 3), nsmall=3))) %>% dplyr::select(-df) %>%
    kable(digits = 3,col.names=c("Group","Task","Emmean","Std. Error","lower CL", "upper CL", "$z$", "$p$", ""), align="llrrrr",
        caption = "Estimated marginal means for group by task") %>% kable_styling()

emm_results <- emmeans(m6type, ~ task.HC. * group.S., type="response") # "Same results as above. This emmeans model is used only for plotting (no 'revpairwise')."
emm_df <- as.data.frame(emm_results)

# plot
emplot<-ggplot(emm_df, aes(x = task.HC., y = prob, color = group.S., group = group.S.)) +
  geom_line(size = 1.5) +
  geom_point(size = 3) +
  geom_ribbon(aes(ymin = asymp.LCL, ymax = asymp.UCL, fill = group.S.), alpha = 0.2) +
  labs(
   y = "Accuracy for each task",
  #  x = "Task",
    title = "Model-predicted accuracy using emmeans."
  ) +
  theme_minimal(base_size = 14) +
  theme(
    axis.text = element_text(color = "black"),
    legend.title = element_blank()
  )

summary(emm_g.t$contrasts)  %>% mutate(
    significance = case_when(
      p.value < 0.001 ~ "***",
      p.value < 0.01 ~ "**",
      p.value < 0.05 ~ "\\*",
      p.value < 0.1 ~ ".",
      TRUE ~ ""),
    p.value = ifelse(p.value<0.001, "<0.001", format(round(p.value, 3), nsmall=3))) %>% 
    dplyr::select(-df) %>% 
    kable(digits = 3,col.names=c("Contrast","Task","Estimate","Std. Error","lower CL", "upper CL","$z$", "$p$", ""), align="llrrrrrrl",
        caption = "Estimated marginal means contrasts for group by task") %>% kable_styling()

remove(emm_g.t, emm_df, emm_results)
```

Estimated marginal means for group by task

| Group | Task | Emmean | Std. Error | lower CL | upper CL | \(z\) | \(p\) |  |
| --- | --- | --- | --- | --- | --- | --- | --- | --- |
| US | 1 | 0.393 | 0.081 | 0.234 | 0.552 | 4.844 | <0.001 | \*\*\* |
| NZ | 1 | 0.442 | 0.081 | 0.284 | 0.600 | 5.475 | <0.001 | \*\*\* |
| US | 2 | 0.951 | 0.089 | 0.777 | 1.125 | 10.697 | <0.001 | \*\*\* |
| NZ | 2 | 1.415 | 0.093 | 1.232 | 1.597 | 15.197 | <0.001 | \*\*\* |
| US | 3 | 1.203 | 0.126 | 0.956 | 1.450 | 9.549 | <0.001 | \*\*\* |
| NZ | 3 | 1.517 | 0.128 | 1.266 | 1.768 | 11.854 | <0.001 | \*\*\* |

Estimated marginal means contrasts for group by task

| Contrast | Task | Estimate | Std. Error | lower CL | upper CL | \(z\) | \(p\) |  |
| --- | --- | --- | --- | --- | --- | --- | --- | --- |
| NZ - US | 1 | 0.049 | 0.114 | -0.175 | 0.272 | 0.428 | 0.669 |  |
| NZ - US | 2 | 0.464 | 0.118 | 0.233 | 0.694 | 3.938 | <0.001 | \*\*\* |
| NZ - US | 3 | 0.314 | 0.120 | 0.079 | 0.549 | 2.619 | 0.009 | \*\* |

```
m6type<- readRDS("exp2/m6type.rds")
#p<-emmip(m6type, task.HC.~group.S., (CIs=TRUE))
#p

pp1<-plot_model(m6type, type = "eff", terms =c("task.HC.", "group.S."), show.legend = T, line.size = 1, show.data =F, ci.lvl=0.95, bias_correction  =T) 
pp1 + labs(y = "Accuracy for each task", x = "Task", title = "Predicted accuracy from the model") + 
  theme(text = element_text(size = 14),
        axis.text = element_text(colour = "black"))+
 theme(legend.title = element_blank()) 

##  figure for the paper
#ggsave(file="exp2/Figure 2-WordLearningR.png", width=8, height=6, dpi=300)
#ggsave(file="exp2/Figure 2-WordLearningR.tiff", device = "tiff",width=8, height=6, dpi=600)
remove(pp1)
```

Predicted accuracy from the best model for each group across tasks.
Interaction plot between group and phase from the final model. Estimated
95% confidence intervals are shown for the predictors. Bias correction
was applied for this plot. The x-axis shows each phase in the
experiment, and the y-axis shows the predicted proportion of accurate
responses

Variance inflation factor (VIF) measures how much the variance of a
regression coefficient is inflated due to multicollinearity in the
model. All VIFs for the final model are below 2, which indicates low
multicollinearity.

```
vif.mer(m6type) %>%
  data.frame() %>%
  rownames_to_column(var = "Factor") %>%
 setNames(c("Factor", "VIF")) %>%
  mutate(
    VIF = round(VIF, 2)
  ) %>% 
  mutate(Factor = Factor %>%
       str_replace_all(.,
          "(?<=^|\\:)(?:c\\.\\()([^:]+)(?:\\))(?=$|\\:)",
          "\\1 (centered)") %>%
        str_replace_all("p.scr", "phonotactic score")%>%
        str_replace_all("nm.nbr", "neighborhood.occupancy.rate")) %>%
 kable(caption="Variance Inflation Factor (VIF) for the Word Learning Task model.", label=NA) %>% kable_styling()

remove(m6type)
```

Variance Inflation Factor (VIF) for the Word Learning Task model.

| Factor | VIF |
| --- | --- |
| group.S.US | 1.01 |
| task.HC.1vs2-3 | 1.53 |
| task.HC.2vs3 | 1.52 |
| typeword | 1.00 |
| group.S.US:task.HC.1vs2-3 | 1.01 |
| group.S.US:task.HC.2vs3 | 1.00 |

### 3.7.3 Post hoc Analysis

#### 3.7.3.1 The relationship between the stimuli and those ratings in Panther et al.’s identification task

Thus, it is questionable whether these words are in the proto-lexicon
for these NMS. We therefore returned our real word stimuli and compared
it with Panther et al.’s (2023) identification task. The plot below
shows the proportion of correct definitions for each word and their
relation to NMS participants’ mean wordhood confidence ratings using a
1-to-5 scale in the Panther et al.’s (2023) identification task. The
higher the wordhood confidence rating is, the more NMS in Panther et
al. (2023) were confident that the stimulus was a real word. The words
that were selected as stimuli for Experiment 2 are highlighted in light
blue. These words were accurately defined between 40 to 60% of the times
in the definition task. While we selected words as stimuli for
Experiment 2 in a narrow band of accuracy rate, these words spread
across ratings in the Panther et al.’s data. At the same time, some
words were not defined with greater than chance accuracy and were rated
lower than three. Although we assumed these words are in the
proto-lexicon of NMS, this may not be the case.

```
## extract IDT ratings
#identTaskOriginal<-read.table(exp2/"IDTOriginal.FP.tsv", sep ="\t", header = TRUE) #%>% dplyr::select (c(1:30))
#length(unique(identTaskOriginal$workerId)) #183
#length(unique(identTaskOriginal$word)) #544

#names(identTaskOriginal)

#Exp1item <-identTaskOriginal  %>% filter(stimulus %in% item.summary_55$stimulus) %>% dplyr::select(stimulus, pair.number, type)  %>% distinct_all()

## assign pair.numbers to DT item summary
#Exp1item <-identTaskOriginal  %>% filter(stimulus %in% item.summary_55$stimulus) %>% dplyr::select(stimulus, pair.number, type)  %>% distinct_all()

## get stimuli used in DF from the original IDT data
#identTaskExp1 <-identTaskOriginal  %>% filter(pair.number %in% Exp1item$pair.number) 

#length(unique(identTaskExp1$workerId)) #183
#length(unique(identTaskExp1$word)) #292 (146 pairs)

#identTaskExp1$enteredResponse<-as.numeric(identTaskExp1$enteredResponse)
#ratingWord_Nonword_Exp1<-aggregate(enteredResponse~type+word+pair.number, identTaskExp1, mean)
#write.table(ratingWord_Nonword_exp1,row.names=FALSE, sep ="\t",file = "FP.IDT.rating-Exp1.tsv")

## Exp2-stimuli only
#identTaskExp2<-identTaskMH  %>% filter(word %in% Exp2_stimuli$word)
#length(unique(identTaskExp2$workerId)) #183
#length(unique(identTaskExp2$word)) #96

#identTaskExp2$enteredResponse<-as.numeric(identTaskExp2$enteredResponse)
#ratingWord_Nonword<-aggregate(enteredResponse~type+word+pair.number, identTaskExp2, mean)

#write.table(ratingWord_Nonword,row.names=FALSE, sep ="\t",file = "FP.IDT.rating-Exp2.tsv")
```

```
IDTaskRatingExp1 <- read.delim("exp2/FP.IDT.rating-Exp1.tsv", sep ="\t", header = TRUE, encoding="UTF-8")
item.summary_def_idtOriginal<-left_join (item.summary_55,IDTaskRatingExp1) 

#nb_res<- item.summary_def_idtOriginal%>% dplyr::select (Exp2, word) %>% distinct_all() %>% group_by(Exp2)%>%  summarise (count = n()) # False 98, True46

# proto-lexical effect (word rating)
idtRate_plot = ggplot(item.summary_def_idtOriginal, aes(x=word_avg, y=item_accuracy_mean, label = stimulus, colour )) + 
  geom_point(size=4, alpha=0.7, shape=17, aes(color = Exp2)) + 
  scale_color_manual(values = c("FALSE" = "#FFA500", "TRUE" = "#4682B4"))+
  geom_text(hjust=0.2, vjust=-0.7, check_overlap=TRUE, size=4.5, color="black") + 
  #geom_smooth(method="lm", color = "blue", fill = "red") +
   theme_minimal() +
  scale_x_continuous(expand=expansion(mult=0.1)) +
  #ylim(-1, 1.05)+
  theme_bw()  +
   theme(text = element_text(size = 12)) +
   ylab('Proportion of correct definitions') + xlab('') + 
  theme(axis.text.x=element_text(hjust = 1, face="bold",size = 12))+
  theme(axis.text.y=element_text(face="bold",size = 12))+
  theme(panel.grid.major = element_line(color = "grey"))+
  xlab("Mean wordhood confidence rating from Panther et al.'s identification task") +
  ylab("Proportion of correct definitions") 

idtRate_plot
#ggsave("./Figure 3-wordList.tiff",  width=8, height=6, device = "tiff",dpi=600)
#ggsave(file="Figure 3-Word List.png", width=10, height=6, dpi=600)

remove(item.summary,item.summary_def_idtOriginal,item.summary, idtRate_plot, IDTaskRatingExp1)
```

The relationship between proportion of correct definitions for each word
and their mean wordhood confidence ratings from Panther et al.’s (2023)
identification task.

#### 3.7.3.2 Word Learning Results in Relation to the Ratings in Panther’s Identification Task

We hypothesized that the IDT rating (mean wordhood confidence ratings
of each word) in Panther et al. s’ (2023) data would predict learning.
NMS would learn words that receive higher wordhood confidence ratings in
the identification task in Panther et al. (2023) than words with lower
ratings.

The plots below show the accuracy rate (proportion of correct
definitions) for each stimulus by IDT rating. It seems that across three
tasks, NMS’ accuracy increases with IDT rating. However, this tendency
is less likely to relate to the stimulus type (i.e., word/nonword).

```
## read an original IDT rating file (Panther et al., 2023)
IDTaskRatingExp2<- read.delim("exp2/FP.IDT.rating-Exp2.tsv", sep ="\t", header = TRUE, encoding="UTF-8") %>% dplyr::rename(IDTrating = enteredResponse)

## convert single characters to digraphs
IDTaskRatingExp2$stimuli <- IDTaskRatingExp2$word %>% 
  str_replace_all("N", "ng") %>% 
  str_replace_all("f", "wh") 

## merge two data files
Data <-left_join (Data,IDTaskRatingExp2) 

## the effect of IDT rating
plot1_IDTrating = Data %>% dplyr::select(correct, type, task, group, IDTrating, word) %>% 
  #filter(task%in% c("2","3")) %>%
  group_by(group,IDTrating, type, task, word) %>% 
  dplyr::summarize(
    mean_correct = mean(as.numeric(correct)),
    n = n()
  ) %>%
  ungroup() %>%
   ggplot(., aes(x=IDTrating, y=mean_correct, color=group)) +
    geom_point(aes(shape=group), alpha=0.3, size=4) +
    geom_smooth(aes(fill=group), method="lm", formula="y~x", alpha=0.6, size=1)+
    facet_grid(~task, labeller="label_both", switch="y")+ 
  xlab("IDT rating") +
  ylab("Accuracy rate") 

plot2_IDTrating = Data %>% dplyr::select(correct, type, task, group, IDTrating, word) %>% ungroup %>%
  #filter(task%in% c("2","3")) %>%
  group_by(group,IDTrating, type, task, word) %>% 
  dplyr::summarize(
    mean_correct = mean(as.numeric(correct)),
    n = n()
  ) %>%
  ungroup() %>%
   ggplot(., aes(x=IDTrating, y=mean_correct, color=type)) +
    geom_point(aes(shape=type), alpha=0.3, size=4) +
    geom_smooth(aes(fill=type), method="lm", formula="y~x", alpha=0.6, size=1)+
    facet_grid(~group, labeller="label_both", switch="y")+ 
  xlab("IDT rating") +
  ylab("Accuracy rate") 

ggarrange (plot1_IDTrating, plot2_IDTrating)
remove(plot1_IDTrating, plot2_IDTrating, IDTaskRatingExp2)
```

left: Mean accuracy of each group for each stimulus across tasks and
their relation to mean wordhood confidence rating (IDT rating) from
Panther et al. (2023). right: Mean accuracy of each group for each
stimulus for real words and non-words and their relation to mean
wordhood confidence rating (IDT rating) from Panther et al. (2023).

**Logistic Regression Model with a variable IDT Rating (continuous
variable)**

We analysed the data using logistic regression in order to test the
hypothesis. We set out a model with a three-way interaction between
`group` (US, NZ), `task` (1,2,3) and
`IDT rating` (mean wordhood confidence rating for each
stimulus from Panther et al.’s (2023) participants).

The model results show that in general, the accuracy rate (proportion
of correct definitions) for each stimulus increases by IDT rating for
NMS. However, we detected only a marginal 3-way interaction between
group, task and IDT rating.

```
#str(Data)
Data$accuracy <- as.logical(as.integer(Data$correct))
#Data$correct<-as.factor(Data$correct)
Data$word<-as.factor(Data$word)
Data$PID<-as.factor(Data$PID) 
Data$group<-as.factor(Data$group)
Data$task<-as.factor(Data$task)
Data$type<-as.factor(Data$type)
Data$education<-as.factor(Data$education)

#contrasts(Data$task.HC.)

## fixed-effects setting,intercepts only
#m1IDTrating <-  glmer(accuracy ~ group.S.*c.(IDTrating)*task.HC.+education +(1|PID)+(1|word), family= binomial, data=Data,control=glmerControl(optimizer="bobyqa", optCtrl=list(maxfun=2e+05)))
#summary(m1IDTrating) 
# Removal candidate: education, marginal interaction in  group.S.:c.(IDTrating):task.HC.2vs3

#m2IDTrating<- update(m1IDTrating, . ~ . - education)
#anova(m1IDTrating, m2IDTrating, test="LRT") # Removal justified Pr(>Chisq  0.6879
#summary(m2IDTrating) # no removal candidate

## mixed-effects setting: add by-participant and by-word random slopes
#m3IDTrating<- glmer(accuracy ~ group.S.*c.(IDTrating)*task.HC.+(1+c.(IDTrating)|PID)+(1+group.S.+task.HC.|word), family= binomial, data=Data,control=glmerControl(optimizer="bobyqa", optCtrl=list(maxfun=2e+05)))
#summary(m3IDTrating)
#saveRDS(m3IDTrating, file = "docs_exp2/m3IDTrating.rds")
#vif.mer(m3IDTrating) #<3

## read the model
m3IDTrating<- readRDS("exp2/m3IDTrating.rds")

logistic_table(m3IDTrating)
```

| Parameter | Estimate | Std. Error | z | p |  |
| --- | --- | --- | --- | --- | --- |
| (Intercept) | 0.983 | 0.065 | 15.014 | <0.001 | \*\*\* |
| group.S. US | -0.139 | 0.053 | -2.612 | 0.009 | \*\* |
| IDTrating (centered) | 0.180 | 0.084 | 2.143 | 0.032 |  |
| task.HC. 1vs2-3 | -0.851 | 0.065 | -13.077 | <0.001 | \*\*\* |
| task.HC. 2vs3 | -0.179 | 0.096 | -1.856 | 0.063 | . |
| group.S. US : IDTrating (centered) | -0.080 | 0.048 | -1.679 | 0.093 | . |
| group.S.US : task.HC. 1vs2-3 | 0.171 | 0.034 | 4.990 | <0.001 | \*\*\* |
| group.S.US : task.HC. 2vs3 | -0.070 | 0.043 | -1.611 | 0.107 |  |
| IDTrating (centered) : task.HC. 1vs2-3 | -0.101 | 0.117 | -0.861 | 0.389 |  |
| IDTrating (centered) : task.HC. 2vs3 | -0.024 | 0.173 | -0.137 | 0.891 |  |
| group.S.US : IDTrating (centered) : task.HC. 1vs2-3 | 0.006 | 0.062 | 0.101 | 0.919 |  |
| group.S.US : IDTrating (centered) : task.HC. 2vs3 | 0.141 | 0.079 | 1.793 | 0.073 | . |

```
## post hoc test
m3IDTrating<- readRDS("exp2/m3IDTrating.rds")

p1 <- emmip(
  m3IDTrating,
  group.S. ~ IDTrating | task.HC.,
  at = list(IDTrating = c(2,3,4,5)),
  CIs = TRUE,
  type = "response"
)

p1$data$task.HC. <- factor(p1$data$task.HC., levels = c("1","2","3"))
p1$data$group.S. <- factor(p1$data$group.S., levels = c("US","NZ"))
p1 <-p1 +
  facet_wrap(~ task.HC., labeller = label_value) +
  scale_color_manual(values = c("US" = "#F8766D", "NZ" = "#00BFC4")) +
  theme_minimal() +
  labs(
    x = "IDTrating",
    y = "Predicted value",
    color = "Group"
  )

emtrends(m3IDTrating,~ group.S.|task.HC., var="IDTrating",infer =TRUE) %>%  emm_tidy() %>%
  kable(digits = 3,col.names=c("Group","Task","Estimate","Std. Error","lower CL","upper CL","$z$", "$p$", ""),align="lrrrrrrl",
        caption = "Estimated marginal means of linear trends by group") %>%  kable_styling()
```

Estimated marginal means of linear trends by group

| Group | Task | Estimate | Std. Error | lower CL | upper CL | \(z\) | \(p\) |  |
| --- | --- | --- | --- | --- | --- | --- | --- | --- |
| US | 1 | 0.037 | 0.086 | -0.131 | 0.204 | 0.430 | 0.668 |  |
| NZ | 1 | 0.188 | 0.086 | 0.020 | 0.356 | 2.197 | 0.028 | \* |
| US | 2 | 0.190 | 0.105 | -0.015 | 0.396 | 1.814 | 0.070 | . |
| NZ | 2 | 0.213 | 0.119 | -0.021 | 0.447 | 1.783 | 0.075 | . |
| US | 3 | 0.073 | 0.191 | -0.302 | 0.447 | 0.381 | 0.703 |  |
| NZ | 3 | 0.378 | 0.197 | -0.009 | 0.764 | 1.913 | 0.056 | . |

```
pp<-plot_model(m3IDTrating, type = "eff", terms =c("IDTrating","task.HC.","group.S."),ci.lvl=0.95, show.legend = T, line.size = 2, show.data =F) 
p2<-pp+ labs(y = "Word definition accuracy", title = "Predicted word learning task")

ggarrange(p1, p2)
remove(p1, pp, p2)
```

Interaction plots from the model showing the interaction among group,
task, and mean wordhood confidence ratings (IDT rating) from Panther et
al. (2023).

As shown below, the VIF test confirms that all fixed effects have a
VIF lower than 5.0, which indicates that all fixed effects are within an
acceptable range.

```
vif.mer(m3IDTrating) %>%
  data.frame() %>%
  rownames_to_column(var = "Factor") %>%
 setNames(c("Factor", "VIF")) %>%
  mutate(
    VIF = round(VIF, 2)
  ) %>% 
  mutate(Factor = Factor %>%
       str_replace_all(.,
          "(?<=^|\\:)(?:c\\.\\()([^:]+)(?:\\))(?=$|\\:)",
          "\\1 (centered)") %>%
        str_replace_all("p.scr", "phonotactic score")%>%
        str_replace_all("nm.nbr", "neighborhood.occupancy.rate")) %>%
 kable(caption="Variance Inflation Factor (VIF) for the Word Learning Task model.", label=NA) %>% kable_styling()
remove(m3IDTrating)
```

Variance Inflation Factor (VIF) for the Word Learning Task model.

| Factor | VIF |
| --- | --- |
| group.S.US | 1.01 |
| IDTrating (centered) | 2.23 |
| task.HC.1vs2-3 | 1.53 |
| task.HC.2vs3 | 1.53 |
| group.S.US:IDTrating (centered) | 1.04 |
| group.S.US:task.HC.1vs2-3 | 1.01 |
| group.S.US:task.HC.2vs3 | 1.01 |
| IDTrating (centered):task.HC.1vs2-3 | 2.25 |
| IDTrating (centered):task.HC.2vs3 | 1.68 |
| group.S.US:IDTrating (centered):task.HC.1vs2-3 | 1.01 |
| group.S.US:IDTrating (centered):task.HC.2vs3 | 1.01 |

# 4 Experiment 3: Wellformedness Rating Task and Word Identification Task

## 4.1 Data Preparation and Method

### 4.1.1 Material

- `wellForm` 20 nonword stimuli were sampled from each
  of the high, medium, and low phonotactic score bins, for a total of 60
  stimuli.
- `wordIdent` 91 real words and 91 matched nonwords were
  sampled from the high and mid frequency bins, for a total of 182
  stimuli. The stimulus set contains 96 items that are used in
  Experiment1.

For both tasks, each participant in the experiment received the same
sample but the sample was shuffled into a random order for each
participant. No stimuli were shared between the WRT and the IDT.

See Panther et al. (2023) for detailed material.

## 4.2 Task Procedure

- `Wellformedness Task` Participants were presented with
  each stimulus one at a time and rated it on a 5-point Likert scale based
  on how Māori-like they perceived it to be. A rating of 1 was labeled as
  “*non Māori-like nonword*”, while a rating of 5 was labeled as
  “*highly Māori-like nonword*”.
- `Identification Task` Participants were presented with
  each stimulus one at a time and rated it on a 5-point Likert scale based
  on their confidence that it was a Māori word. A rating of 1 was labeled
  as “*confident that this is NOT a Māori word*”, while a rating of
  5 was labeled as “*confident that this IS a Māori
  word*”.

## 4.3 Data Cleaning Process and Filtering

65 participants completed the entire experiment. We applied the same
measure used in Experiment 1 to evaluate potential external aid usage,
given the absence of time constraints in this task. Two participants
exceeded the threshold for median reaction time. We compared their
median reaction times between real-word and nonword trials. For one
participant, the difference was negligible (0.023 seconds), and for the
other, it was modest (0.509 seconds). Given the minimal differences and
the robustness of the median to outliers, there is no evidence of
systematic aid usage.

One participant was excluded whose response variability (i.e., the
standard deviation [SD] of 1–5 ratings) was more than two standard
deviations below the group mean. Thus, the data consisted of 64
participants.

## 4.4 Wellformedness Rating Task

The analysis of the Wellformedness rating task focuses on
participants’ phonotactic knowledge. Models test whether trends of
participants’ sensitivity to Māori phonotactics.

**Data Structure**  The dataset is structured as follows:

- *PID* is the unique ID for each participant.
- *enteredResponse* is the wellformedness ratings for each
  stimulus.
- *word* is the stimulus used for the rating.
- *phonotactic score* is the phonotactic score for each
  word.
- *neighborhood.occupancy.rate* is the proportion of potential
  phonological neighbors of a stimulus (i.e., phonotactically legal items
  with an edit distance of one from the stimulus) that correspond to a
  real Māori word.

```
# read a file and rename columns
wellFormTask<- read.delim("exp3/wellFormTask.tsv", sep ="\t", header = TRUE, encoding = "UTF-8") #%>% rename("p.scr" = "score.shortv", "freq.c" = "freq.category",  "nm.nbr"= "norm.neighbors")

#length(unique(wellFormTask$PID)) #64
```

### 4.4.1 Mean Rating and Phonotactic Score for Each Stimulus

The plot shows the mean rating of stimuli in the Wellformedness
Rating Task by the phonotactic score of the stimuli. The results show a
positive correlation between the rating of stimuli and their phonotactic
score (\(r = 0.64\)).

```
#str(wellFormTask)
#length(unique(wellFormTask$PID)) #64
wellFormTask$PID= as.factor(wellFormTask$PID)
wellFormTask$enteredResponse = as.factor(wellFormTask$enteredResponse)
wellFormTask$word = as.factor(wellFormTask$word)

# summary for stimulus characteristics
summary_table_wellForm <- wellFormTask%>%
  distinct(word, .keep_all = TRUE) %>%   
  summarise(
    Mean_Length = mean(n.phonemes, na.rm = TRUE),
    SD_Length = sd(n.phonemes, na.rm = TRUE),
    Mean_Phonotactic_Score = mean(p.scr, na.rm = TRUE),
    SD_Phonotactic_Score = sd(p.scr, na.rm = TRUE),
    Mean_Neighborhood_Density = mean(nm.nbr, na.rm = TRUE),
    SD_Neighborhood_Density = sd(nm.nbr, na.rm = TRUE)
  ) %>%
  mutate(across(where(is.numeric), ~ round(.x, 2)))

#print(summary_table_wellForm )
remove(summary_table_wellForm)

# get mean ratings for each stimulus per bin
allBinsScored <- wellFormTask %>%
  group_by(word, bin=tert_count, p.scr) %>% summarise(meanRating = mean(as.numeric(enteredResponse)))
    
# get boundaries between bins
boundaries <- c(max(allBinsScored$p.scr[allBinsScored$bin == "low"]), max(allBinsScored$p.scr[allBinsScored$bin == "medium"]))

rating_by_phonotactics <- ggplot(allBinsScored, aes(x=p.scr, y=meanRating)) +
  geom_point(alpha=0.1, size=4) +
  #geom_smooth(method="lm", formula="y~x", alpha=0.7, size=1, color="black") +
  geom_smooth(method="lm",aes(x=p.scr, y=meanRating)) +
  geom_vline(xintercept=boundaries, linetype="dotted") +
  xlab("Phonotactic score") +
  ylab("Mean rating (per stimulus)") +
  ylim(1, 5) +
  theme_bw() + 
  theme(
    panel.grid = element_blank()
  )
rating_by_phonotactics + stat_cor(method="spearman") 

remove(allBinsScored, rating_by_phonotactics, boundaries)
```

Mean Wellformedness Rating by Phonotactic Score. Dotted lines separate
items into bins of equal size by phonotactic score.

```
#str(wellFormTask)
# begin with Fixed Effects Model

# CLM 
#Wellform00 <- clm(enteredResponse ~ c.(p.scr)* c.(nm.nbr), data=wellFormTask)
#summary(Wellform00) 
# removal candidate; c.(p.scr):c.(nm.nbr)

#Wellform01 <- update(Wellform00, . ~ . - c.(p.scr):c.(nm.nbr)) 
#summary(Wellform01) 
# no more removal candidates

#CLMM

#Wellform02<- clmm(enteredResponse ~ c.(p.scr) + c.(nm.nbr) + (1 + c.(p.scr) + c.(nm.nbr)| PID) + (1| word), data=wellFormTask)
#summary(Wellform02)
#saveRDS(Wellform02, file = "docs2/Wellform02.rds")
# only phonotactic score is significant
```

### 4.4.2 Summary of Model

```
Wellform02<- readRDS("exp3/Wellform02.rds")

# obtain 95% CI
#ci(Wellform02)
#model_parameters(Wellform02 effects = "all")
names(Wellform02$coefficients) <- names(Wellform02$coefficients)%>% str_replace_all("p.scr", "phonotactic score") %>% str_replace_all("nm.nbr", "neighborhood.occupancy.rate")

clm_table(Wellform02, caption = "Best-Fitting Model for the Word Wellformedness Task",label=NA)
```

Best-Fitting Model for the Word Wellformedness Task

|  | Parameter | Estimate | Std. Error | z | p |  |
| --- | --- | --- | --- | --- | --- | --- |
| Effects | phonotactic score (centered) | 5.560 | 0.925 | 6.012 | <0.001 | \*\*\* |
|  | neighborhood.occupancy.rate (centered) | -2.213 | 2.678 | -0.826 | 0.409 |  |
| Thresholds | 1¦2 | -2.684 | 0.180 |  |  |  |
|  | 2¦3 | -1.138 | 0.175 |  |  |  |
|  | 3¦4 | 0.014 | 0.174 |  |  |  |
|  | 4¦5 | 1.776 | 0.177 |  |  |  |

**Variance Inflation Factors (VIFs) for the Word Identification Task
Model**

We report Variance Inflation Factors (VIFs) for the model. All fixed
effects have a VIF < 5.0, meaning that all fixed effects are within
an acceptable range.

```
Wellform02<- readRDS("exp3/Wellform02.rds")

#method="logistic", Hess=TRUE
# Fit an ordinal logistic regression model
p_Wellform02 <- polr(Wellform02)

# Calculate VIF for each predictor variable
Wellform02_vif<-vif(p_Wellform02)

Wellform02_vif%>%
  data.frame() %>%
  rownames_to_column(var = "Factor") %>%
 setNames(c("Factor", "VIF")) %>%
  mutate(
    VIF = round(VIF, 2)
  ) %>% 
  mutate(Factor = Factor %>%
       str_replace_all(.,
          "(?<=^|\\:)(?:c\\.\\()([^:]+)(?:\\))(?=$|\\:)",
          "\\1 (centered)") %>%
        str_replace_all("p.scr", "phonotactic score")%>%
        str_replace_all("nm.nbr", "neighborhood.occupancy.rate") %>%
        str_replace_all(., fixed(":"), " x "),)%>%
 kable(caption="Variance Inflation Factor (VIF) for the Wellformedness Rating Task model.", label=NA) %>% kable_styling()

remove(Wellform02, Wellform02_vif, p_Wellform02)
```

Variance Inflation Factor (VIF) for the Wellformedness Rating Task
model.

| Factor | VIF |
| --- | --- |
| phonotactic score (centered) | 1.13 |
| neighborhood.occupancy.rate (centered) | 1.13 |

## 4.5 Word Identification Task

### 4.5.1 Data Structure

```
identTask<-read.table("exp3/identTask2.tsv", sep ="\t", header = TRUE, encoding = "UTF-8") 

#length(unique(identTask$word)) ; length(unique(identTask$PID)) #182 # 64
```

The dataset is structured as follows:

- *PID* is the unique ID for each participant.
- *enteredResponse* is the wellformedness ratings for each
  stimulus.
- *word* is the stimulus used for the rating.
- *type* is the classification of each stimulus: word (‘real’)
  or non-word (‘pseudo’).
- *phonotactic.sore* is the phonotactic score for each
  word.
- *n.phoneme* is the phoneme length of each stimulus.
- *freq.category* is the classification of each stimulus: high
  or mid.

### 4.5.2 Mean Rating and Phonotactic Score for Each Stimulus for Stimulus Types by Frequency Bin

```
## Code for Figure: Plot mean ratings per bin, per stimulus type
## Get mean ratings for each stimulus per bin

#str(identTask)

identTask$PID= as.factor(identTask$PID)
identTask$enteredResponse = as.factor(identTask$enteredResponse)
identTask$type = as.factor(identTask$type)
identTask$freq.c = as.factor(identTask$freq.c)
identTask$word = as.factor(identTask$word)

# summary for stimulus characteristics
#names(identTask)
summary_table_ident<- identTask %>%
  distinct(word, type,.keep_all = TRUE) %>% group_by(type) %>%
  summarise(
    Mean_Length = mean(n.phon, na.rm = TRUE),
    SD_Length = sd(n.phon, na.rm = TRUE),
    Mean_Phonotactic_Score = mean(p.scr, na.rm = TRUE),
    SD_Phonotactic_Score = sd(p.scr, na.rm = TRUE),
    Mean_Neighborhood_Density = mean(norm.neighbors, na.rm = TRUE),
    SD_Neighborhood_Density = sd(norm.neighbors, na.rm = TRUE)
  ) %>%
  mutate(across(where(is.numeric), ~ round(.x, 2)))

#print(summary_table_ident)

#Get mean ratings for each stimulus per bin
allBins <- list()
bin_names <- c("mid", "high")
for(i in 1:length(bin_names)){
  Bin <- identTask[identTask$freq.c==bin_names[i],]
  BinMeanRatings <- aggregate(as.numeric(Bin$enteredResponse), by=list(Bin$word, Bin$type, Bin$Exp), mean)
  names(BinMeanRatings) <- c("word","type","Exp","meanRating")
  BinMeanRatings$bin <- bin_names[i]
  allBins[[i]] <- BinMeanRatings
}
remove(i, Bin, BinMeanRatings)

allBins <- do.call(rbind,allBins)
allBins$type = factor(allBins$type)
allBins$Exp2 = factor(allBins$Exp)
levels(allBins$type) = c("Nonword", "Word")
allBins$bin <- factor(allBins$bin, levels=c("mid","high"))
allBins$Exp2<-factor(allBins$Exp2, levels = c("FALSE","TRUE"))

# Get grand mean ratings for each stimulus type per bin
binMeans <- allBins %>% 
  group_by(type, bin, Exp) %>% 
  dplyr::summarize(grand_mean = mean(meanRating))

 ggplot(allBins,aes(x = bin, y = meanRating, fill = type)) +
    geom_violin(alpha=0.4, position=position_identity()) + 
    geom_point(data=binMeans, aes(y=grand_mean, shape=type), size=4) +
    geom_line(data=binMeans, aes(x=as.numeric(as.factor(bin)), y=grand_mean, color=type, linetype=type), size=1) + 
    scale_fill_manual(name="Stimulus type",
                      values = c("Nonword" = "black", "Word" = "blue"),
                      guide = guide_legend(reverse = TRUE)) +  
    scale_shape_manual(name="Stimulus type",
                      values = c("Nonword" = 21, "Word" = 24),
                      guide = guide_legend(reverse = TRUE)) +   
    scale_color_manual(name="Effect estimate",
                       values = c("Nonword"="black", "Word" = "blue"),
                       guide="none") +
    scale_linetype_manual(name="Effect estimate",
                          values = c("Nonword" = "solid", "Word" = "dotted"),
                          guide="none") + 
    labs(y = "Mean rating (by stimulus)",x = "Frequency bin") +
    ylim(1, 5) +
    theme(axis.text=element_text(size=28),
         axis.title=element_text(size=24,face="bold"),
    legend.title=element_text(size=22),
    legend.text=element_text(size=22)) +
    theme_classic() +
    facet_grid(. ~ Exp) +

remove(binMeans)
 
#ggsave("exp2/Figure 4-IdentTask.tiff",  width=8, height=6, device = "tiff",dpi=600)
#ggsave(file="exp2/Figure 4-IdentTask.png", width=8, height=4, dpi=600)
```

Mean wordhood confidence ratings for each stimulus per frequency
category bin across datasets. Points represent mean ratings across all
real words and nonwords within each bin.

### 4.5.3 Mean Rating and Phonotactic Score for Each Stimulus per Frequency Bin

The plot below expands on the visualization of the plot above by
adding a dimension for the phonotactic score of the words and non-words
within each frequency bin. Participants gave higher ratings to words
than non-words. Participants also gave higher ratings to both words and
non-words with higher phonotactic scores. The effect of phonotactic
score was weak in the high-frequency bin (right) compared to the
mid-frequency bin (left).

```
scores <- unique(identTask[,c("word","p.scr")])
allBinsScored <- merge(allBins, scores, by="word")
remove(scores)

discrim_bins_scatter <- ggplot(allBinsScored, aes(x=p.scr, y=meanRating, color=type)) +
  geom_point(aes(shape=type), alpha=0.3, size=3) +
  geom_smooth(aes(fill=type), method="lm", formula="y~x", alpha=0.6, size=1) +
  facet_grid(~ bin, labeller="label_both", switch="y") +
  xlab("Phonotactic score") +
  ylab("Mean rating (per stimulus)") +
  scale_shape_manual(name="Stimulus type",
                    values = c("Nonword" = 1, "Word" = 2),
                    guide = guide_legend(reverse = TRUE)) +   
  scale_color_manual(name="Stimulus type",
                     values = c("Nonword"="black", "Word" = "blue"),
                     guide = guide_legend(reverse = TRUE)) +
  scale_fill_manual(name="Stimulus type",
                     values = c("Nonword"="gray70", "Word" = "dodgerblue1"),
                     guide = guide_legend(reverse = TRUE)) +  
  ylim(1, 5) +
  theme_bw() + 
  theme(
    panel.grid = element_blank()
  ) 

discrim_bins_scatter

# ggsave("plots/discrim_bins_scatter.jpg", plot = discrim_bins_scatter , width = 7, height = 4, dpi = 200)
remove(allBins, allBinsScored, bin_names, discrim_bins_scatter)

#ggsave("docs2/Figure4.png")
```

Mean wordhood confidence rating vs. phonotactic score for each stimulus
for real words and nonwords by frequency category bin across two groups.
Lines show correlations within each bin, for each stimulus type.

### 4.5.4 Regression Models: General Identification Performance (all stimuli)

We analyse whether participants are able to discriminate real words
from nonwords by two three-way interactions: c.(p.scr) x type x freq.c
as in the previous analysis, testing the current participants have
knowledge of Māori like participants in our previous experiments,
c.(p.scr) x type x Exp based on the results of Exp2.

```
#str(identTask)
#tableType<-identTask %>% count(enteredResponse, type, sort = TRUE)
identTask$PID <- as.factor(identTask$PID)
identTask$enteredResponse <- as.factor(identTask$enteredResponse)
identTask$word <- as.factor(identTask$word)
identTask$type <- as.factor(identTask$type)
#identTask <- identTask %>% mutate(
#    Exp2_stimuli = ifelse(Exp2 == FALSE, "No",
#      "Yes"), Exp2_stimuli= factor(Exp2_stimuli))
identTask$Exp<- as.factor(identTask$Exp)

## change the factor levels in freq.c
identTask<-identTask%>% mutate(freq.c=fct_relevel(freq.c, c("mid","high")))

#nb_res<- identTask %>% dplyr::select (Exp, word) %>% distinct_all() %>% group_by(Exp)%>%  summarise (count = n()) # 64,96,22

## phonotactic.score*type*frequency, c.(p.scr)*Exp*type
## c.(p.scr) * type * freq.c :  as in the previous analysis, testing the current participants have knowledge of Māori like participants in our previous experiments. 
## c.(p.scr) * type * Exp : based on the results of Exp2.

## CLM
#Exp3Ident01 <- clm(enteredResponse ~ c.(p.scr)*type*(freq.c + Exp) + c.(n.phon), data=identTask)
#summary(Exp3Ident01)
# removal candidate: c.(p.scr):type:freq.c

#Exp3Ident02<- update(Exp3Ident01,. ~ . -c.(p.scr):type:freq.c)
#summary(Exp3Ident02)
# removal candidate: no removal candidate

## CLMM : Exp included by a word-slope
#Exp3Ident03a <- clmm(enteredResponse ~ c.(p.scr) * type * Exp  + (c.(p.scr) + type) * freq.c + c.(n.phon) + (1+Exp|word) + (1+c.(p.scr) + type + freq.c + c.(n.phon)|PID),  data=identTask)
#summary(Exp3Ident03a)
#saveRDS(Exp3Ident03a, file = "exp3/Exp3Ident03a.rds")
# convergence error: Variance-covariance matrix of the parameters is not defined

## CLMM : Exp included by a PID-slope
#Exp3Ident03 <- clmm(enteredResponse ~ c.(p.scr) * type * Exp  + (c.(p.scr) + type) * freq.c + c.(n.phon) + (1|word) + (1+c.(p.scr) + type + freq.c + c.(n.phon) + Exp |PID),  data=identTask)
#summary(Exp3Ident03)
#saveRDS(Exp3Ident03, file = "exp3/Exp3Ident03.rds")
# removal candidate: c.(p.scr):typeword:Exp

#Exp3Ident04　<- update(Exp3Ident03, . ~ . -c.(p.scr):type:Exp)
#anova(Exp3Ident03, Exp3Ident04) # Pr(>Chisq)       0.1778
#summary(Exp3Ident04)
#saveRDS(Exp3Ident04, file = "exp3/Exp3Ident04.rds")
## removal candidate: Exp:freq.c

#Exp3Ident05　<- update(Exp3Ident04, . ~ . -c.(p.scr):type)
#anova(Exp3Ident04,Exp3Ident05) # Pr(>Chisq)  1
#summary(Exp3Ident05)
#saveRDS(Exp3Ident05, file = "exp3/Exp3Ident05.rds")
## removal candidate: c.(p.scr):Exp

#Exp3Ident06　<- update(Exp3Ident05, . ~ . -c.(p.scr):Exp)
#anova(Exp3Ident05,Exp3Ident06) # Pr(>Chisq)   0.2032
#summary(Exp3Ident06)
#saveRDS(Exp3Ident06, file = "exp3/Exp3Ident06.rds")
## removal candidate: type:freq.c

#Exp3Ident07　<- update(Exp3Ident06, . ~ . -type:freq.c)
#anova(Exp3Ident06,Exp3Ident04) # Pr(>Chisq)  
#summary(Exp3Ident07)
#saveRDS(Exp3Ident07, file = "exp3/Exp3Ident07.rds")
```

### 4.5.5 Summary of Model

```
Exp3Ident07<- readRDS("exp3/Exp3Ident07.rds")
names(Exp3Ident07$coefficients) <- names(Exp3Ident07$coefficients)%>% str_replace_all("p.scr", "phonotactic score") %>% str_replace_all("n.phon", "number of phonemes")

clm_table(Exp3Ident07, caption = "Best-Fitting Model for the Word Identification Task")
```

Best-Fitting Model for the Word Identification Task

|  | Parameter | Estimate | Std. Error | z | p |  |
| --- | --- | --- | --- | --- | --- | --- |
| Effects | phonotactic score (centered) | 6.821 | 1.542 | 4.423 | <0.001 | \*\*\* |
|  | type = word | 1.087 | 0.266 | 4.080 | <0.001 | \*\*\* |
|  | Exp = Exp2 | 0.043 | 0.257 | 0.168 | 0.867 |  |
|  | Exp = Exp3 | -0.004 | 0.380 | -0.010 | 0.992 |  |
|  | freq.c = high | 0.190 | 0.168 | 1.127 | 0.260 |  |
|  | number of phonemes (centered) | 0.248 | 0.119 | 2.089 | 0.037 |  |
|  | type = word : Exp = Exp2 | -0.784 | 0.337 | -2.329 | 0.020 |  |
|  | type = word : Exp = Exp3 | 1.220 | 0.519 | 2.352 | 0.019 |  |
|  | phonotactic score (centered) : freq.c = high | -3.500 | 1.828 | -1.915 | 0.056 |  |
| Thresholds | 1¦2 | -2.137 | 0.249 |  |  |  |
|  | 2¦3 | -0.345 | 0.248 |  |  |  |
|  | 3¦4 | 1.277 | 0.248 |  |  |  |
|  | 4¦5 | 2.816 | 0.249 |  |  |  |


```
# post hoc emmeans test
Exp3Ident07<- readRDS("exp3/Exp3Ident07.rds")

emtrends(Exp3Ident07, ~ freq.c, var="p.scr",infer =TRUE) %>% emm_tidy() %>% 
  kable(digits = 3,col.names=c("Frequency","Estimate","Std. Error","lower CL","upper CL","$z$", "$p$",""),align="lrrrrrrl",
        caption = "Estimated marginal means of linear trends by frequency.") %>% kable_styling()

emm_t.e<-emmeans(Exp3Ident07, revpairwise ~ type|Exp, infer =TRUE)

emm_tidy(emm_t.e$emmeans)  %>%
    kable(digits = 3,col.names=c("Contrast","Stimuli in Experiment","Estimate","Std. Error","lower CL", "upper CL", "$z$", "$p$", ""), align="llrrrr",
        caption = "Estimated marginal means for words/nonwords by experiments.") %>% kable_styling()

emm_tidy(emm_t.e$contrasts) %>%
    kable(digits = 3,col.names=c("Contrast","Stimuli in Experiment","Estimate","Std. Error","lower CL", "upper CL","$z$", "$p$",""), align="llrrrrl",
        caption = "Estimated marginal means contrasts for words/nonwords by experiments.") %>% kable_styling()

remove(emm_t.e)
```

Estimated marginal means of linear trends by frequency.

| Frequency | Estimate | Std. Error | lower CL | upper CL | \(z\) | \(p\) |  |
| --- | --- | --- | --- | --- | --- | --- | --- |
| mid | 6.821 | 1.542 | 3.798 | 9.844 | 4.423 | <0.001 | \*\*\* |
| high | 3.321 | 1.585 | 0.213 | 6.428 | 2.095 | 0.036 | \* |

Estimated marginal means for words/nonwords by experiments.

| Contrast | Stimuli in Experiment | Estimate | Std. Error | lower CL | upper CL | \(z\) | \(p\) |  |
| --- | --- | --- | --- | --- | --- | --- | --- | --- |
| nonword | Exp1 | -0.308 | 0.225 | -0.749 | 0.133 | -1.368 | 0.171 |  |
| word | Exp1 | 0.779 | 0.223 | 0.342 | 1.216 | 3.494 | <0.001 | \*\*\* |
| nonword | Exp2 | -0.265 | 0.192 | -0.641 | 0.111 | -1.379 | 0.168 |  |
| word | Exp2 | 0.038 | 0.185 | -0.325 | 0.401 | 0.205 | 0.838 |  |
| nonword | Exp3 | -0.312 | 0.339 | -0.977 | 0.354 | -0.918 | 0.359 |  |
| word | Exp3 | 1.996 | 0.348 | 1.314 | 2.677 | 5.737 | <0.001 | \*\*\* |

Estimated marginal means contrasts for words/nonwords by experiments.

| Contrast | Stimuli in Experiment | Estimate | Std. Error | lower CL | upper CL | \(z\) | \(p\) |  |
| --- | --- | --- | --- | --- | --- | --- | --- | --- |
| word - nonword | Exp1 | 1.087 | 0.266 | 0.565 | 1.609 | 4.080 | <0.001 | \*\*\* |
| word - nonword | Exp2 | 0.303 | 0.219 | -0.126 | 0.731 | 1.385 | 0.166 |  |
| word - nonword | Exp3 | 2.307 | 0.452 | 1.422 | 3.192 | 5.109 | <0.001 | \*\*\* |

```
plot1<-emmip(Exp3Ident07,type~Exp, CIs=TRUE)
plot2<-emmip(Exp3Ident07,freq.c~p.scr,at=list(p.scr= c(-1.2,-1.0,-0.8,-0.6)), CIs=TRUE)

ggarrange(plot1, plot2)
#ggarrange(plot1, plot2)
remove(plot1, plot2)
```

Two-Way Interactions in the Word Identification Task Model

We report Generalized Variance Inflation Factors (GVIFs) for this
model. Same as other models, we used the function polr () within the
package MASS and the function vif () within the package car. This
produces GVIF, DF (degrees of freedom), and ’GVIF^(1/(2\*DF))’ (GVIF is
normalized by the number of DF of GVIF) for each factor. We use the
square-root of GVIF (Fox and Monette, 1992)

We retrieve the Converted GVIF for each fixed effect in this model.
As shown in the table below, all fixed effects have a VIF < 5,
meaning that all fixed effects are within an acceptable range.

```
Exp3Ident07<- readRDS("exp3/Exp3Ident07.rds")
p_Exp3Ident07<- polr(Exp3Ident07)
Exp3Ident07_vif<-vif(p_Exp3Ident07)

Exp3Ident07_vif %>%
  data.frame() %>%
   setNames(c("GVIF", "DF", "Converted_GVIF"))  %>%
  rownames_to_column(var = "Factor") %>%
 mutate(GVIF = round(GVIF, 2))  %>%
     mutate(
      Factor = Factor %>%
        str_replace_all(.,
          "(?<=^|\\:)(?:c\\.\\()([^:]+)(?:\\))(?=$|\\:)",
          "\\1 (centered)"
        ) %>%
        str_replace_all("p.scr", "phonotactic score"), 
      Converted_GVIF = Converted_GVIF ^ 2
    ) %>%
   mutate(Converted_GVIF= round(Converted_GVIF, 2))  %>%
  kable(caption="Generalised Variance Inflation Factor (GVIF) for the Word Identification Task Model", label=NA)  %>%
  kable_styling()
remove(Exp3Ident07,Exp3Ident07_vif, p_Exp3Ident07)
```

Generalised Variance Inflation Factor (GVIF) for the Word Identification
Task Model

| Factor | GVIF | DF | Converted\_GVIF |
| --- | --- | --- | --- |
| phonotactic score (centered) | 2.46 | 1 | 2.46 |
| type | 2.95 | 1 | 2.95 |
| Exp | 4.12 | 2 | 2.03 |
| freq.c | 1.11 | 1 | 1.11 |
| n.phon (centered) | 1.44 | 1 | 1.44 |
| type:Exp | 6.98 | 2 | 2.64 |
| phonotactic score (centered):freq.c | 1.86 | 1 | 1.86 |

### 4.5.6 Post Hoc Tests for the Word Learning

We also explored whether any differences between participants (top
figures) or between words (bottom figure) in this experiment could
predict differences in a potential lexical effect in experiment 2, but
found no evidence to support this. This is likely due to the fact that
the variability across words and participants is not particularly high,
nor is the total number of words investigated in experiment 2.

Between participants: We used individuals’ wellformedness rating task
performance and word identification task scores.

`Wellformedness Rating Task Performance (WRTP) Score = mean(high-phonotactic score nonword) - mean(low-phonotactic-score nonword)`

The WRTP score is a measure of phonotactic sensitivity.

`Word Identification Task Performance (IDTP) Score = mean(word rating) - mean(nonword rating)`

The IDTP score is a direct measure of the proto-lexicon, assessing
knowledge of the particular words in our test.

In the top left figure, it seems like that participants with higher
phonotactic sensitivity have learned words better than nonwords in task
2 but the effect of WRTP score was not found in statistical models.

```
# Ident task with Experiment2 stimuli
IdentExp2 <-identTask %>% dplyr::select (word, PID, type, pair.number, enteredResponse,Exp, IDTPS, WRTPS) %>% filter (Exp == "Exp2") 
#count<-IdentExp2 %>% count (word, sort = TRUE) #96

# get Experiment2 data = Data
# 70 NZ participants in Exp1 but only 64 in Exp2.
exp2_64 <- Data %>% filter (group == "NZ") %>% filter(PID %in% IdentExp2$PID) %>% rename("p.scr" = "score.shortv", "freq.c" = "freq.category",  "n.phon"= "n.phonemes")%>% mutate(across(c(PID),factor))
#length(unique(exp2_64$PID)) #64
#table(exp2_64$type)
#str(exp2_64)
#names(exp2_64)

# merge two files (exp2 and exp3)
IdentWLexp<-left_join(exp2_64, IdentExp2, by = c("PID", "word","type", "pair.number"),as.dataframe()) 
#8659 #dplyr::select(-c(1,27:29)) 
#str(IdentWLexp)
remove(exp2_64)

WRTPS_exp2<- IdentWLexp%>% dplyr::select(correct, type, task, WRTPS) %>% 
  filter (task %in% c("2", "3"))  %>% group_by(WRTPS, type, task) %>% 
   dplyr::summarize(
    mean_correct = mean(as.numeric(correct)),
    n = n()
  )  %>% 
      ungroup() %>% 
   ggplot(., aes(x=WRTPS, y=mean_correct, color=type)) +
    geom_point(aes(shape=type), alpha=0.3, size=4) +
    geom_smooth(aes(fill=type), method="lm", formula="y~x", alpha=0.6, size=1)+
    facet_grid(~task, labeller="label_both", switch="y")+ 
   xlab("Participants' WRTP Score") +
  ylab("Accuracy rate") 

#WRTPS_exp2 + stat_cor(method="spearman") 

IDTPS_exp2<- IdentWLexp%>% dplyr::select(correct, type, task, IDTPS) %>% 
  filter (task %in% c("2", "3"))  %>% group_by(IDTPS, type, task) %>% 
   dplyr::summarize(
    mean_correct = mean(as.numeric(correct)),
    n = n()
  )  %>% 
      ungroup() %>%
   ggplot(., aes(x=IDTPS, y=mean_correct, color=type)) +
    geom_point(aes(shape=type), alpha=0.3, size=4) +
    geom_smooth(aes(fill=type), method="lm", formula="y~x", alpha=0.6, size=1)+
    facet_grid(~task, labeller="label_both", switch="y")+ 
   xlab("Participants' IDTP score") +
  ylab("Accuracy rate")

#IDTPS_exp2 + stat_cor(method="spearman") 

ggarrange(WRTPS_exp2, IDTPS_exp2)


## Convert  1 and 0 TRUE/FALSE to
#IdentWLexp$accuracy <- as.logical(as.integer(IdentWLexp$correct))
#IdentWLexp$word<-as.factor(IdentWLexp$word)
#IdentWLexp$PID<-as.factor(IdentWLexp$PID) 
#IdentWLexp$group<-as.factor(IdentWLexp$group)
#IdentWLexp$task<-as.factor(IdentWLexp$task)
#IdentWLexp$type<-as.factor(IdentWLexp$type)

##  task * type * WRTPS
## The random effects structure was simplified as necessary by pruning random slopes one at a time until the model converged.

#IdentWLexp$task.HC.<- factor(IdentWLexp$task, levels=c("1","2","3"))
#my.HC = matrix(c(2/3, -1/3, -1/3, 0, .5, -.5), ncol = 2)
#my.HC

#colnames(my.HC) <- c("1vs2-3", "2vs3")
#contrasts(IdentWLexp$task.HC.) = my.HC
#contrasts(IdentWLexp$task.HC.)

#str(IdentWLexp)

## three way interaction, a model does not include a factor "education"
## predict accuracy using group, type, task
## correct (int or factor): results are the same
## accuracy = logit

## fixed-effects setting,intercepts only
#m1_exp2WPS<-  glmer(accuracy ~ type*task.HC.*c.(WRTPS) +(1|PID)+(1|word), family= binomial, data=IdentWLexp,control=glmerControl(optimizer="bobyqa", optCtrl=list(maxfun=2e+05)))
#summary(m1_exp2WPS) # Removal candidate: type:task.HC.:WRTPS
#m2_exp2WPS <- update(m1_exp2WPS, . ~ . - type:task.HC.:c.(WRTPS))
#anova(m1_exp2WPS, m2_exp2WPS, test="LRT") # Removal justified Pr(>Chisq 0.4213)
#summary(m2_exp2WPS) # Removal candidate: group.S.:type 
#m3_exp2WPS<- update(m2_exp2WPS, . ~ . - type:task.HC.)
#anova(m2_exp2WPS, m3_exp2WPS, test="LRT") # Removal justified Pr(>Chisq  0.8703)
#summary(m3_exp2WPS) # Removal candidate: type:task.HC.
# no removal candidate

## mixed-effects setting: add by-participant and by-word random slopes
#m4_exp2WPS<- glmer(accuracy ~ type*WRTPS + task.HC.*c.(WRTPS) +(1+type|PID)+(1+c.(WRTPS)+task.HC.|word), family= binomial, data=IdentWLexp,control=glmerControl(optimizer="bobyqa", optCtrl=list(maxfun=2e+05)))
#summary(m4_exp2WPS)
#saveRDS(m4_exp2WPS, file = "exp3/m4_exp2WPS.rds")
## the effect of WRTPS was not detected
```

The relationship between word learning accuracy and individual task
performance scores

Between words: We used the IDT rating (mean wordhood confidence
ratings of each word) in the Experiment 3 data to predict learning. The
plots below show the accuracy rate (proportion correct definitions) for
each stimulus by IDT rating. It seems that NMS’ accuracy increases with
IDT rating in task1, but not in task 2 and 3. The IDT rating is less
likely to relate to word type (i.e., word/nonword). The effect of IDT
rating was not detected in statistical models.

```
## the effect of IDT rating
IdentExp2$enteredResponse<-as.numeric(IdentExp2$enteredResponse)
ratingWord_Nonword_Exp3<-aggregate(enteredResponse~type+word+pair.number, IdentExp2, mean)
ratingWord_Nonword_Exp3<-ratingWord_Nonword_Exp3%>% dplyr::rename(IDTratingExp3 = enteredResponse)

# merge two files 
IdentWLexp<-left_join(IdentWLexp, ratingWord_Nonword_Exp3, by = c("word","type","pair.number"),as.dataframe())

## the effect of IDT rating
plot1_IDTratingExp3 = IdentWLexp %>% dplyr::select(correct, type, task, group, IDTratingExp3, word) %>% 
  #filter(task%in% c("2","3")) %>%
  group_by(group,IDTratingExp3, type, task, word) %>% 
  dplyr::summarize(
    mean_correct = mean(as.numeric(correct)),
    n = n()
  ) %>%
  ungroup() %>%
   ggplot(., aes(x=IDTratingExp3, y=mean_correct, color=type)) +
    geom_point(aes(shape=type), alpha=0.3, size=4) +
    geom_smooth(aes(fill=type), method="lm", formula="y~x", alpha=0.6, size=1)+
    facet_grid(~task, labeller="label_both", switch="y")+ 
  xlab("IDT rating") +
  ylab("Accuracy rate") 

ggarrange (plot1_IDTratingExp3)
remove(plot1_IDTratingExp3)

## Convert  1 and 0 TRUE/FALSE to
#IdentWLexp$accuracy <- as.logical(as.integer(IdentWLexp$correct))
#IdentWLexp$word<-as.factor(IdentWLexp$word)
#IdentWLexp$PID<-as.factor(IdentWLexp$PID) 
#IdentWLexp$group<-as.factor(IdentWLexp$group)
#IdentWLexp$task<-as.factor(IdentWLexp$task)
#IdentWLexp$type<-as.factor(IdentWLexp$type)

##  task * type * WRTPS
## The random effects structure was simplified as necessary by pruning random slopes one at a time until the model converged.
#IdentWLexp$task.HC.<- factor(IdentWLexp$task, levels=c("1","2","3"))
#my.HC = matrix(c(2/3, -1/3, -1/3, 0, .5, -.5), ncol = 2)
#my.HC

#colnames(my.HC) <- c("1vs2-3", "2vs3")
#contrasts(IdentWLexp$task.HC.) = my.HC
#contrasts(IdentWLexp$task.HC.)

#str(IdentWLexp)

## fixed-effects setting,intercepts only
#m1_exp2IDTrating<-  glmer(accuracy ~ type*task.HC.*c.(IDTratingExp3) +(1|PID)+(1|word), family= binomial, data=IdentWLexp,control=glmerControl(optimizer="bobyqa", optCtrl=list(maxfun=2e+05)))
#summary(m1_exp2IDTrating) # Removal candidate: type:task.HC.:c.(IDTratingExp3)
#m2_exp2IDTrating <- update(m1_exp2IDTrating, . ~ . - type:task.HC.:c.(IDTratingExp3))
#anova(m1_exp2IDTrating, m2_exp2IDTrating , test="LRT") # Removal justified Pr(>Chisq 0.8317)
#summary(m2_exp2IDTrating) # Removal candidate: group.S.:type 
#m3_exp2IDTrating<- update(m2_exp2IDTrating, . ~ . - type:task.HC.)
#anova(m2_exp2IDTrating, m3_exp2IDTrating, test="LRT") # Removal justified Pr(>Chisq   0.8064)
#summary(m3_exp2IDTrating) # Removal candidate: task.HC.:c.(IDTratingExp3)
#m4_exp2IDTrating<- update(m3_exp2IDTrating, . ~ . - task.HC.:c.(IDTratingExp3))
#anova(m3_exp2IDTrating, m4_exp2IDTrating, test="LRT") # Removal justified Pr(>Chisq   0.5304)
#summary(m4_exp2IDTrating) # Removal candidate: type:c.(IDTratingExp3)

## mixed-effects setting: add by-participant and by-word random slopes
#m5_exp2IDTrating<- glmer(accuracy ~ type + task.HC. + c.(IDTratingExp3) +(1+type|PID)+(1+c.(IDTratingExp3)+task.HC.|word), family= binomial, data=IdentWLexp,control=glmerControl(optimizer="bobyqa", optCtrl=list(maxfun=2e+05)))
#summary(m5_exp2IDTrating)
#saveRDS(m5_exp2IDTrating, file = "exp3/m5_exp2IDTrating.rds")
# the effect of the identification task raing was not detected 

remove(ratingWord_Nonword_Exp3, IdentWLexp)
remove(WRTPS_exp2, IDTPS_exp2, IdentExp2)
```

The relationship between word learning accuracy and IDT rating

# 5 Questionnaires

## 5.1 Questionnaire for New Zealanders

The post experiment questionnaire consisted of 26 questions. There
were four question types: (i) yes/no questions; (ii) single answer out
of multiple options; (iii) multiple answers out of multiple options;
(iv) text input. For the purpose of coding answers, options in Questions
1, 2, 13, 14 were assigned numerical values, based on the number of
answers the participant gave.

1. How well are you able to speak Māori?

   - Not at all (0)
   - No more than a few words for phrases (1)
   - Not very well (2)
   - Fairly well (3)
   - Well (4)
   - Very well (5)
2. How well are you able to understand/read Māori?

   - Not at all (0)
   - No more than a few words or phrases (1)
   - Not very well (2)
   - Fairly well (3)
   - Well (4)
   - Very well (5)
3. What is the highest level of education you have studied Te Reo
   Māori?

   - At primary/intermediate school
   - At high school
   - At a polytechnic or in an adult education course
   - At undergraduate level in university
   - At postgraduate level in university
   - Never studied it
4. Have you ever taken a university-level course in linguistics?

   - Yes, I have taken a linguistics course
   - No, but I have studied a language at university
   - No, I have no university-level study in linguistics or
     languages
5. Which age group do you belong to?

   - 18-29
   - 30-39
   - 40-49
   - 50-59
   - +60
6. Please state your gender. *(free response)*
7. Please state your ethnicity. *(free response)*
8. Were you born in New Zealand?

   - Yes
   - No
9. Which island have you spent more time on?

   - North Island
   - South Island
   - Equal measure on both
10. In the time since you were 7, have you ever lived outside of NZ
    for a period of more than a year?

    - Yes
    - No
11. In which region have you spent the largest amount of time since
    you were 7?

    - Northland
    - Auckland
    - Waikato
    - Bay of Plenty
    - Gisborne
    - Hawke’s Bay
    - Taranaki
    - Wanganui
    - Manawatu
    - Wairarapa
    - Wellington
    - Nelson Bays
    - Marlborough
    - West Coast
    - Canterbury
    - Timaru
    - Oamaru
    - Otago
    - Southland
    - Overseas
12. Your highest education is:

    - High School
    - Certificate or Diploma
    - Undergraduate Degree
    - Graduate Degree
13. How often do you think you are exposed to Māori language in your
    daily life, by means of Māori radio, Māori TV, online media?

    - Less than once a year (1)
    - Less than once a month (2)
    - Less than once a week (3)
    - Less than once a day (4)
    - Multiple times a day (5)
14. How often do you think you are exposed to Māori language in your
    daily life, in conversation at work, at home, in social settings?

    - Less than once a year (1)
    - Less than once a month (2)
    - Less than once a week (3)
    - Less than once a day (4)
    - Multiple times a day (5)
15. In the past five years, have you had any children living with you
    who have attended preschool or primary school in New Zealand?

    - Yes
    - No
16. Please tick all boxes that apply.

    - I can give a mihi in Māori
    - I can sing a few songs in Māori
    - I can sing a NZ national anthem in Māori
    - I know how to say some basic phrases (e.g. My name is…, I’m from…)
      in Māori
    - I know how to say some commands (e.g. Sit down / Come here) in
      Māori
    - I know how to say some greetings in Māori
    - I know how to say some numbers in Māori
    - I know how to say some body parts in Māori
    - I know how to say some colors in Māori
17. What region of New Zealand do you live in currently? (Please
    choose “overseas” if you are living outside of New Zealand).

    - Northland
    - Auckland
    - Waikato
    - Bay of Plenty
    - Gisborne
    - Hawke’s Bay
    - Taranaki
    - Wanganui
    - Manawatu
    - Wairarapa
    - Wellington
    - Nelson Bays
    - Marlborough
    - West Coast
    - Canterbury
    - Timaru
    - Oamaru
    - Otago
    - Southland
    - Overseas
18. How long have you been living there?

    - less than six months
    - 1 year
    - 2 years
    - 3 years
    - 4 years
    - 5 years
    - 6 years
    - 7 years
    - 8 years
    - 9 years
    - 10+ years
    - 20+ years
    - 30+ years
    - 40+ years
    - 50+ years
19. Please state your first language (the language you speak/use most
    of your time). *(free response)*
20. Please list any other languages that you can speak well.
    *(free response)*
21. What country were you living in when you first learned this
    language? *(free response)*
22. Have you lived in Hawaii?

    - Yes
    - No
23. Do you speak/understand any Polynesian languages such as
    Hawaiian, Tahitian, Sāmoan, or Tongan?

    - Yes
    - No
24. If you replied yes to the Polynesian language question, please
    state the language you know. *(free response)*
25. How do you feel about the following statement: “I have a lot of
    respect for people who can speak Māori fluently.”

    - Strongly disagree
    - Somewhat disagree
    - Neither agree nor disagree
    - Somewhat agree
    - Strongly agree
26. How do you feel about the following statement: “Some Māori
    language education should be compulsory in school for all children.”

    - Strongly disagree
    - Somewhat disagree
    - Neither agree nor disagree
    - Somewhat agree
    - Strongly agree
27. Did you participate in an online Māori word rating experiment or
    word splitting experiment earlier this year? (only appeared in the Word
    Definition Task)

    - Yes, rating one
    - Yes, splitting one
    - Both of them
    - Not sure
    - No

## 5.2 Questionnaire for Americans

The post experiment questionnaire consisted of 23 questions. For the
purpose of coding answers, options in Questions 1, 2, 12, 13 were
assigned numerical values, based on the number of answers the
participant gave.

1. How well are you able to speak Māori?

   - Not at all (0)
   - No more than a few words for phrases (1)
   - Not very well (2)
   - Fairly well (3)
   - Well (4)
   - Very well (5)
2. How well are you able to understand/read Māori?

   - Not at all (0)
   - No more than a few words or phrases (1)
   - Not very well (2)
   - Fairly well (3)
   - Well (4)
   - Very well (5)
3. What is the highest level of education you have studied Te Reo
   Māori?

   - At primary/intermediate school
   - At high school
   - At a polytechnic or in an adult education course
   - At undergraduate level in university
   - At postgraduate level in university
   - Never studied it
4. Have you ever taken a university-level course in linguistics?

   - Yes, I have taken a linguistics course
   - No, but I have studied a language at university
   - No, I have no university-level study in linguistics or
     languages
5. Which age group do you belong to?

   - 18-29
   - 30-39
   - 40-49
   - 50-59
   - +60
6. Please state your gender. *(free response)*
7. Please state your ethnicity. *(free response)*
8. Were you born in the USA?

   - Yes
   - No
9. In the time since you were 7, have you ever lived outside of the
   USA for a period of more than a year?

   - Yes
   - No
10. What country or state have you spent the largest amount of time
    since you were 7? *(free response)*
11. Your highest education is:

    - High School
    - Certificate or Diploma
    - Undergraduate Degree
    - Graduate Degree
12. How often do you think you are exposed to Māori language in your
    daily life, by means of Māori radio, Māori TV, online media?

    - Less than once a year (1)
    - Less than once a month (2)
    - Less than once a week (3)
    - Less than once a day (4)
    - Multiple times a day (5)
13. How often do you think you are exposed to Māori language in your
    daily life, in conversation at work, at home, in social settings?

    - Less than once a year (1)
    - Less than once a month (2)
    - Less than once a week (3)
    - Less than once a day (4)
    - Multiple times a day (5)
14. In the past five years, have you had any children living with you
    who have attended preschool or primary school in New Zealand?

    - Yes
    - No
15. Please tick all boxes that apply.

    - I can give a mihi in Māori
    - I can sing a few songs in Māori
    - I can sing a NZ national anthem in Māori
    - I know how to say some basic phrases (e.g. My name is…, I’m from…)
      in Māori
    - I know how to say some commands (e.g. Sit down / Come here) in
      Māori
    - I know how to say some greetings in Māori
    - I know how to say some numbers in Māori
    - I know how to say some body parts in Māori
    - I know how to say some colors in Māori
16. What country do you live in currently? *(free
    response)*
17. How long have you been living there?

    - less than six months
    - 1 year
    - 2 years
    - 3 years
    - 4 years
    - 5 years
    - 6 years
    - 7 years
    - 8 years
    - 9 years
    - 10+ years
    - 20+ years
    - 30+ years
    - 40+ years
    - 50+ years
18. Please state your first language (the language you speak/use most
    of your time). *(free response)*
19. What country were you living in when you first learned this
    language? *(free response)*
20. Please list any other languages that you can speak well.
    *(free response)*
21. Have you lived in Hawaii?

    - Yes
    - No
22. Do you speak/understand any Polynesian languages such as
    Hawaiian, Tahitian, Sāmoan, or Tongan?

    - Yes
    - No
23. If you replied yes to the Polynesian language question, please
    state the language you know. *(free response)*

*Debriefing Statement* (which appeared after the
questionnaire)

Thank you for taking part in our research project.

This experiment was designed to see whether the unconscious knowledge
that people have about the Māori language can help people to learn the
meanings of Māori words. In order to fully test the possibilities, some
of the words you learnt were not real Māori words. In addition, the
meanings you learnt for the real Māori words were not the real
meanings.

To protect the integrity of this research, we could not fully divulge
all the details of this study at the start of the procedure. As you
know, your participation in this study is voluntary. If you so wish, you
may withdraw after reading this debriefing page without any penalty, at
which point all records of your participation will be destroyed. If you
would like to withdraw, please contact the researcher (email address)
within two days.

# 6 Stimulus Materials

## 6.1 Word Definition Task

```
defShortWordList<- defShort%>% ungroup %>%  dplyr::select(stimulus) %>% distinct() %>% arrange(stimulus)
listword <- paste(defShortWordList$stimulus, collapse=", ")
kable(listword, caption="List of Stimuli for Word Definition Task (146 words)",col.names = NULL)
remove(defShortWordList)
```

List of Stimuli for Word Definition Task (146 words)


|  |
| --- |
| aho, ako, amo, anake, anei, arero, ariki, atua, aua, aute, awanga, haere, hape, hine, hinu, hiwi, hoa, hoatu, hoko, hongi, hono, horoi, huaki, huna, hune, ihu, ika, ingoa, inoi, ipu, iri, iwa, kahika, kanawa, kani, kawiti, kehu, kino, kiore, kiwa, korou, kotahi, kotiti, kuaka, kuhu, kume, kuru, mahara, maire, makau, makawe, mangu, mano, marara, mariu, maroke, maunga, mohi, momi, mura, muri, nawe, nehe, ngaki, ngaro, ngau, ngeru, ngote, nguru, ngutu, niao, noa, noho, nui, nuku, oho, ora, pahure, pakake, pakari, pango, piro, pito, puare, puea, puhi, puni, punua, rahi, raki, rango, raru, rawa, rawe, raweke, reira, rere, rewa, ruaki, rui, runga, tahaki, takao, takoto, tanguru, taniwha, tanu, tapahi, tapepe, taringa, tawhito, tihe, tinana, tino, tio, tohu, tongi, tono, tonu, toromi, tuku, tumeke, tunu, uhi, uira, uma, umere, umu, waha, waia, waro, wawe, wehe, wehi, wete, weu, whaene, whanga, whano, whare, whati, whatu, whenua, whero, whiore, whiri |

## 6.2 Word Learning Task

```
WordLearningWordList<- Data%>% ungroup %>%  dplyr::select(stimuli) %>% distinct() %>% arrange(stimuli)
listword <- paste(WordLearningWordList$stimuli, collapse=", ")
kable(listword, caption="List of Stimuli for Word Learning Task (96 words)",col.names = NULL)
remove(Data, WordLearningWordList)
```

List of Stimuli for Word Learning Task (96 words)


|  |
| --- |
| anake, apako, arero, awanga, awete, awiri, hape, hehi, hene, hini, hinu, hohi, hohu, hono, hoti, huna, kangi, kapepe, kawiti, keihi, kinu, kiwa, koene, koho, korou, kuaka, kuaki, kure, kuro, kuru, kuto, makau, manao, mangu, mariu, maroke, mawa, mehe, mohi, momi, moreke, moru, nawe, nehe, ngaro, ngiri, nguru, niki, nongi, noto, nuku, patau, pito, pokua, porei, puhu, punua, rahi, raia, raki, ramu, raru, rawa, raweke, rawera, reira, rere, rete, rewa, rina, riwi, tahaki, takao, tapahi, tapati, tapepe, tariki, tariu, tawharo, tawhito, tawiki, tongi, tuku, umene, umere, waia, wanga, wehe, wete, whaene, whake, whano, whari, whato, whatu, whiri |

## 6.3 Wellformedness Rating Task

```
wellFormStimuli<-wellFormTask%>% dplyr::select(stimulus) %>% distinct(stimulus) %>% arrange(stimulus)
listword <- paste(wellFormStimuli$stimulus, collapse=", ")
kable(listword, caption="List of Stimuli for Wellformedness Rating Task (60 nonwords)",col.names = NULL)
remove(wellFormStimuli, wellFormTask)
```

List of Stimuli for Wellformedness Rating Task (60
nonwords)


|  |
| --- |
| angu, apao, awara, eko, hano, haraka, hiaki, hoke, ieke, iroa, karara, karora, kengi, kohiki, kohini, korei, koreo, kotoa, manai, marao, matuke, meki, minga, moio, munu, nai, ngaoro, noe, ongi, pakao, pangi, parana, pawe, poara, porohi, puhe, pukiri, rahe, riei, roui, takonu, tanawe, tapera, tapire, taure, tehu, tenana, teu, tokine, touru, tuhe, uoro, uriki, wetua, whangi, wheri, whetere, wheto, whiao, wihu |

## 6.4 Word Identification Task

```
identStimuli<-identTask%>% dplyr::select(stimulus) %>% distinct(stimulus) %>% arrange(stimulus) 
listword <- paste(identStimuli$stimulus, collapse=", ")
kable(listword, caption="List of stimuli for Word Identification Task (91 real words/ 91 nonwords)",col.names = NULL)
remove(identStimuli, identTask)
```

List of stimuli for Word Identification Task (91 real words/ 91
nonwords)


|  |
| --- |
| ako, anake, anei, apako, arero, arou, aua, aui, auku, aute, awanga, awete, awiri, eri, haere, hape, hehi, hene, hingo, hini, hinu, hoatu, hohi, hohu, hongi, honiwha, hono, hoti, hui, huku, huna, ika, iko, kaini, kaiti, kangi, kapepe, kauri, kawiti, keihi, kino, kinu, kiwa, koene, koho, korou, kotu, kuaka, kuaki, kuhu, kuma, kume, kure, kuro, kuru, kuto, maire, makau, manao, mangu, marae, maringa, mariu, maroke, mauma, maunga, mawa, mehe, moatu, moha, mohi, moko, momi, moreke, moru, nawe, nea, nehe, ngaki, ngaro, ngiri, ngui, nguru, niki, noa, noho, nongi, noto, nui, nuku, ohe, oho, oto, patau, pauri, peka, pihana, pito, poho, poku, pokua, ponu, porei, potu, puhi, puhu, puki, puku, pungi, puni, punua, pupe, rahi, raia, rakaro, raki, ramu, raru, rawa, raweke, rawera, reira, reki, rere, rete, rewa, rina, riwi, rumu, taea, tahaki, takao, takoto, tangeke, tani, taniwha, taoa, tapahi, tapati, tapepe, tarae, tariki, taringa, tariu, tawharo, tawhito, tawiki, tiaki, tinana, tinu, tipi, tohu, tongi, tonu, tuahe, tue, tuku, tumeke, uhi, uira, uire, uma, umene, umere, une, waha, waia, wanga, waru, wehe, wete, whaene, whake, whano, whari, whato, whatu, whekua, whenga, whenua, whiri, wihi |

**## References**


Fox, J., & Monette, G. (1992). Generalized collinearity
diagnostics. Journal of the American Statistical Association, 87(417),
178-183.

Mattingley, W., Hay, J., Todd, S., Panther, F., King, J., &
Keegan, Peter J. (2024). Ongoing exposure to an ambient language
continues to build implicit knowledge across the lifespan. Linguistic
Vanguard. 10(1), 345-355.

Panther, F. A., Mattingley, W., Todd, S., Hay, J. & King,
J.(2023). Proto-Lexicon Size and Phonotactic Knowledge are Linked in
Non-Māori Speaking New Zealand Adults. Laboratory Phonology 14(1).
